# Supplementary material for: In vitro Cas9-assisted editing of modular polyketide synthase genes to produce desired natural product derivatives
Source: Nat Commun. 2020 Aug 11;11:4022. doi: 10.1038/s41467-020-17769-2 (PMC7419507; doi:10.1038/s41467-020-17769-2)
Supplement: Supplementary file 1 — Supplementary Information [file 41467_2020_17769_MOESM1_ESM.pdf]

In vitro Cas9-assisted Editing of Modular Polyketide Synthase Genes to Produce Desired Natural Product Derivatives

Supplementary information

Kudo et al.

Supplementary Figures 1-11

Supplementary Tables 1-3

Supplementary Data (NMR data) 1-93

Reference

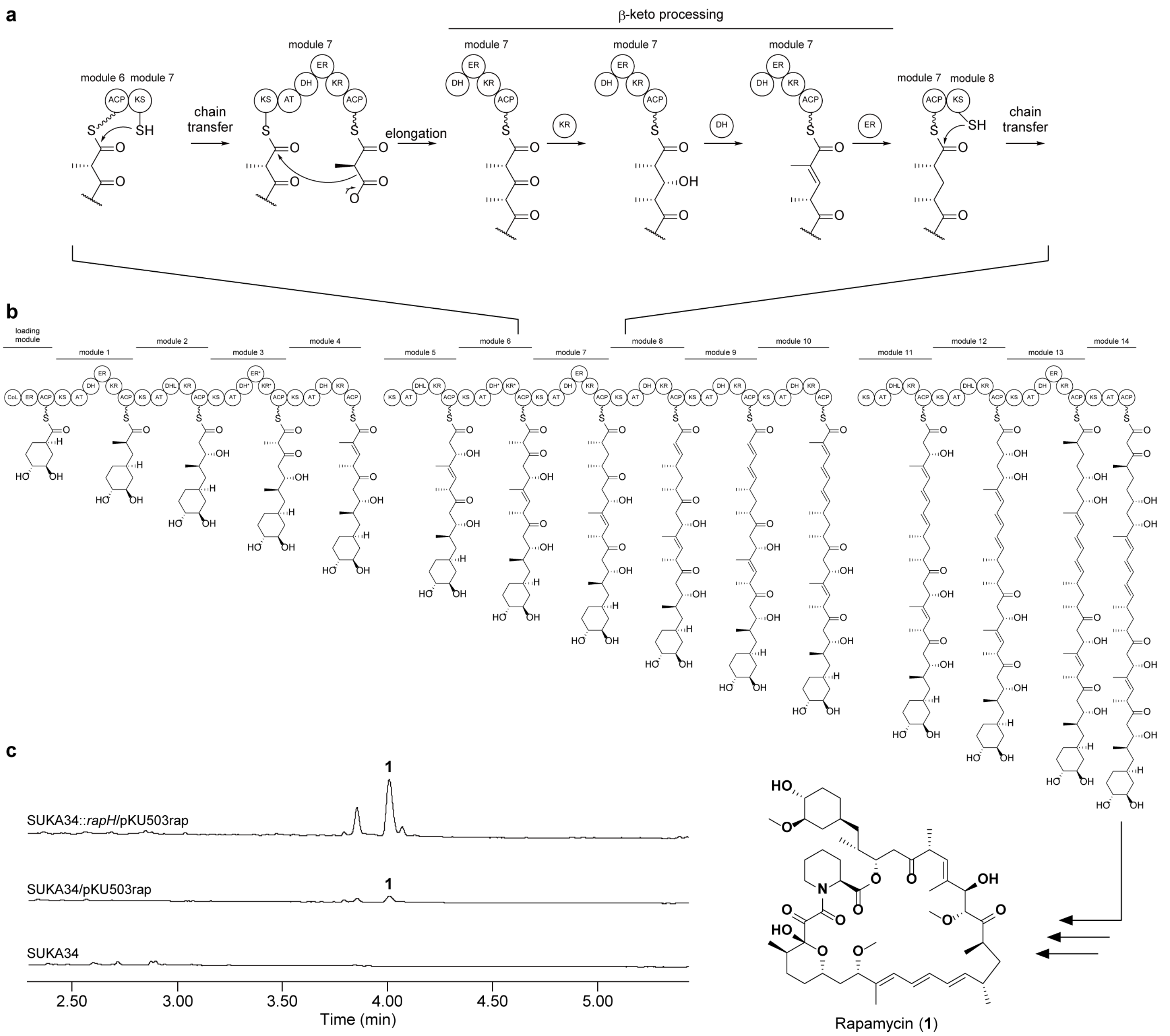

Supplementary Figure 1. Heterologous expression of the biosynthetic gene cluster of rapamycin. **a**, Schematic representation of the reactions catalysed by modular PKS. The nascent polyketide chain of the upstream module (module 6, for example) is transferred to the next module (module 7). The ACP-bound extender unit is used for the chain elongation reaction. The β-keto group is processed by a reductive loop. **b**, Domain organization of the rapamycin PKS. **c**, The BAC clone containing the entire gene cluster for rapamycin, pKU503rap, was expressed in *S. avermitilis* SUKA. The culture extract was analysed by UPLC-TOF-MS. UV chromatograms at 280 nm are shown. The yield of rapamycin (**1**) estimated from the peak areas was 8.6 mg L^{-1}. Each chromatogram shown is a representative of at least three biological duplicates.

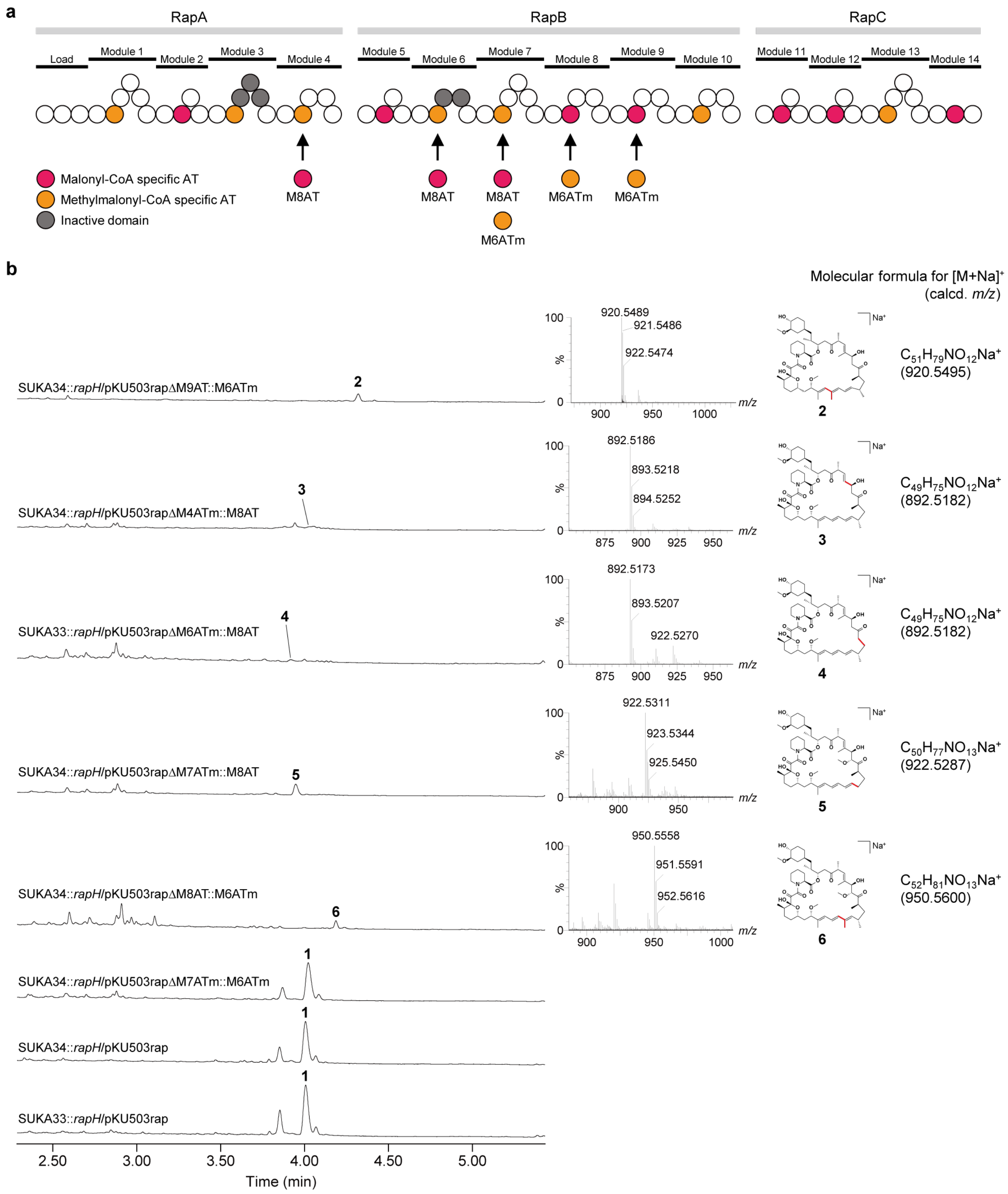

Supplementary Figure 2. AT-swapping via "in vitro module editing". **a**, Illustration of target ATs and exchanged ATs. AT domains specific for malonyl-CoA were filled with magenta, and those for methylmalonyl-CoA were filled in yellow. Circles filled with grey indicated inactive domains. **b**, Comparative HPLC analysis of the metabolites. UV chromatograms at 280 nm are shown. The HR-ESI-MS data, the structures of the corresponding derivative and the molecular formula for $[M+Na]^+$ are indicated in

the column to the right of each chromatogram. Each chromatogram shown is a representative of at least three biological duplicates.

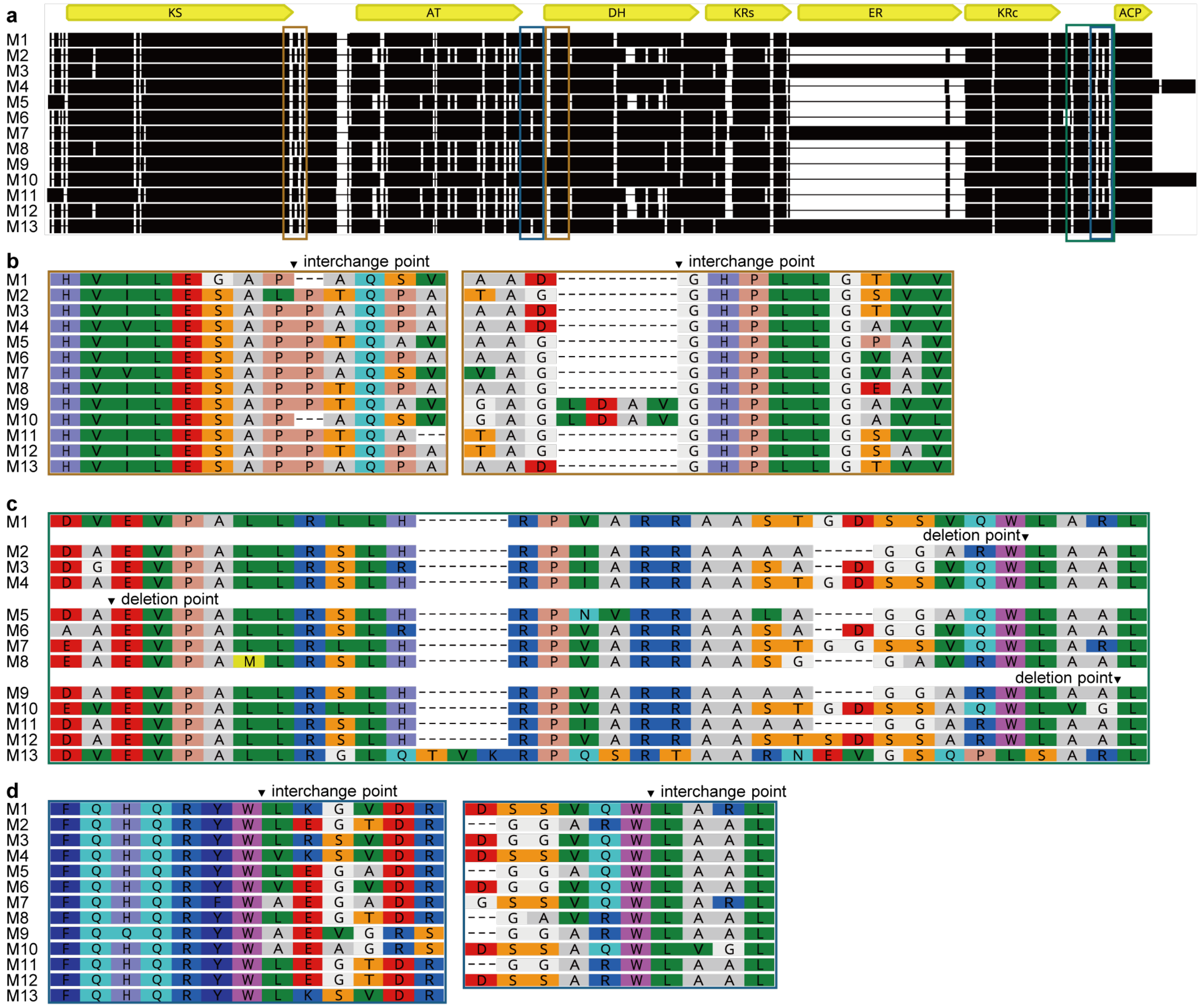

Supplementary Figure 3. Editing point used in this study. **a**, Overview of sequence homology. The black bars indicate identical bases. The colour of the box corresponds to the following panels. **b**, Interchange points for AT-swapping. The upstream boundary for the exchange of the AT domains was set at the C-terminal of the KS domain, LESAP/PT in the amino acid sequence, and the downstream boundary was set at the N-terminal of the DH domain, LDAV/GHPLLG in the amino acid sequence (the slash ("/") indicates the interchange points). **c**, Deletion points for one-module deletions. The module to be deleted was cut off of the residue indicated by the triangle to the same position as in the next module. **d**, Interchange points for reductive loop manipulations. The upstream boundary for the exchange of reductive loops was set after the conserved tryptophan residue located in the N-terminal flanking region of the DH domain. The downstream boundary was set after the conserved tryptophan residue located in the N-terminal flanking region of the ACP domain.

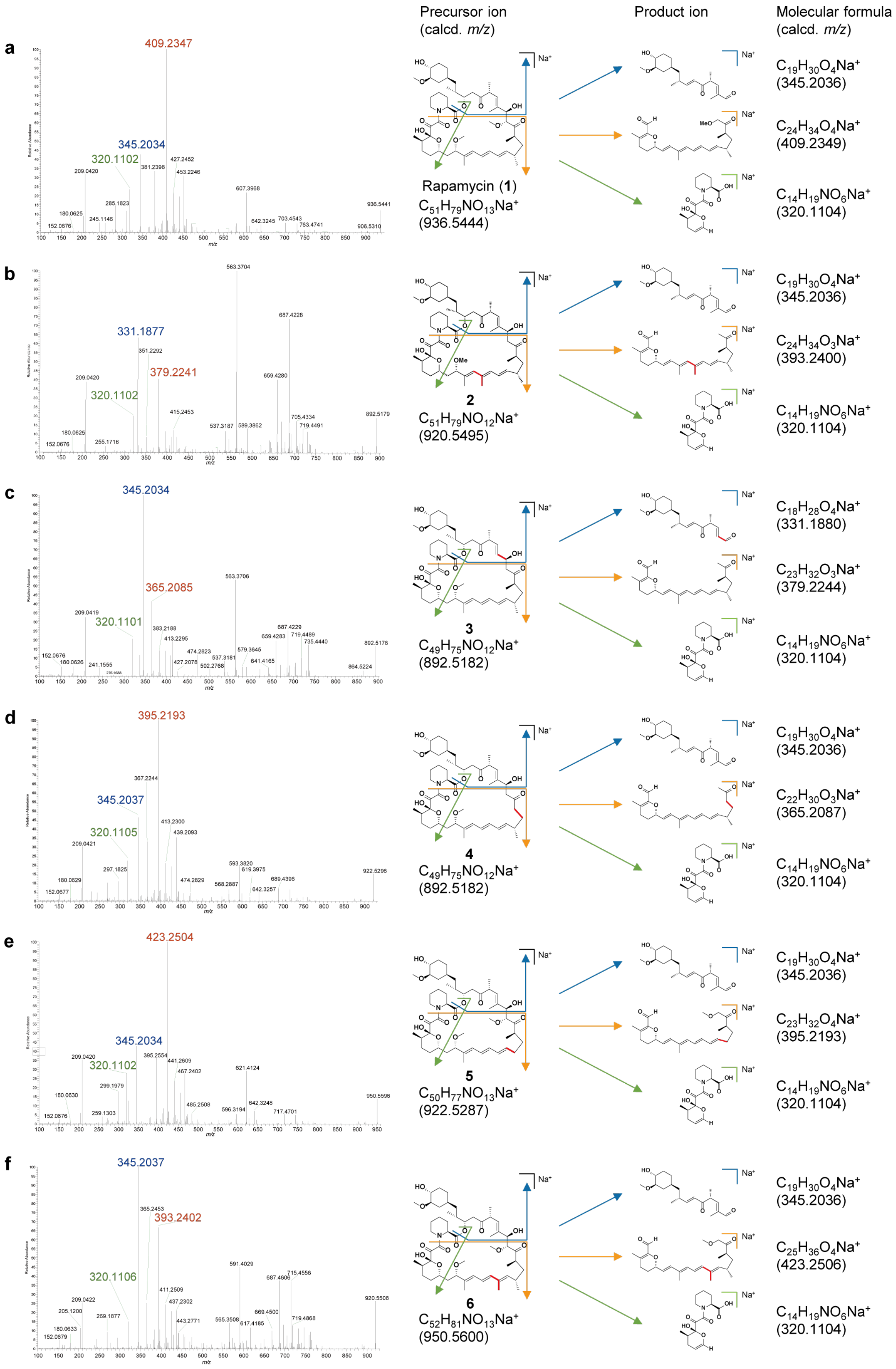

Supplementary Figure 4. MS/MS analysis of rapamycin derivatives produced by AT substitutions. **a**, The fragmentation pattern of rapamycin (**1**)[1]. **b**, **c**, **d**, **e** and **f** show the fragmentation patterns of AT-substituted rapamycin derivatives **2**, **3**, **4**, **5**, and **6**, respectively. Each spectrum shown is a representative of at least three biological duplicates.

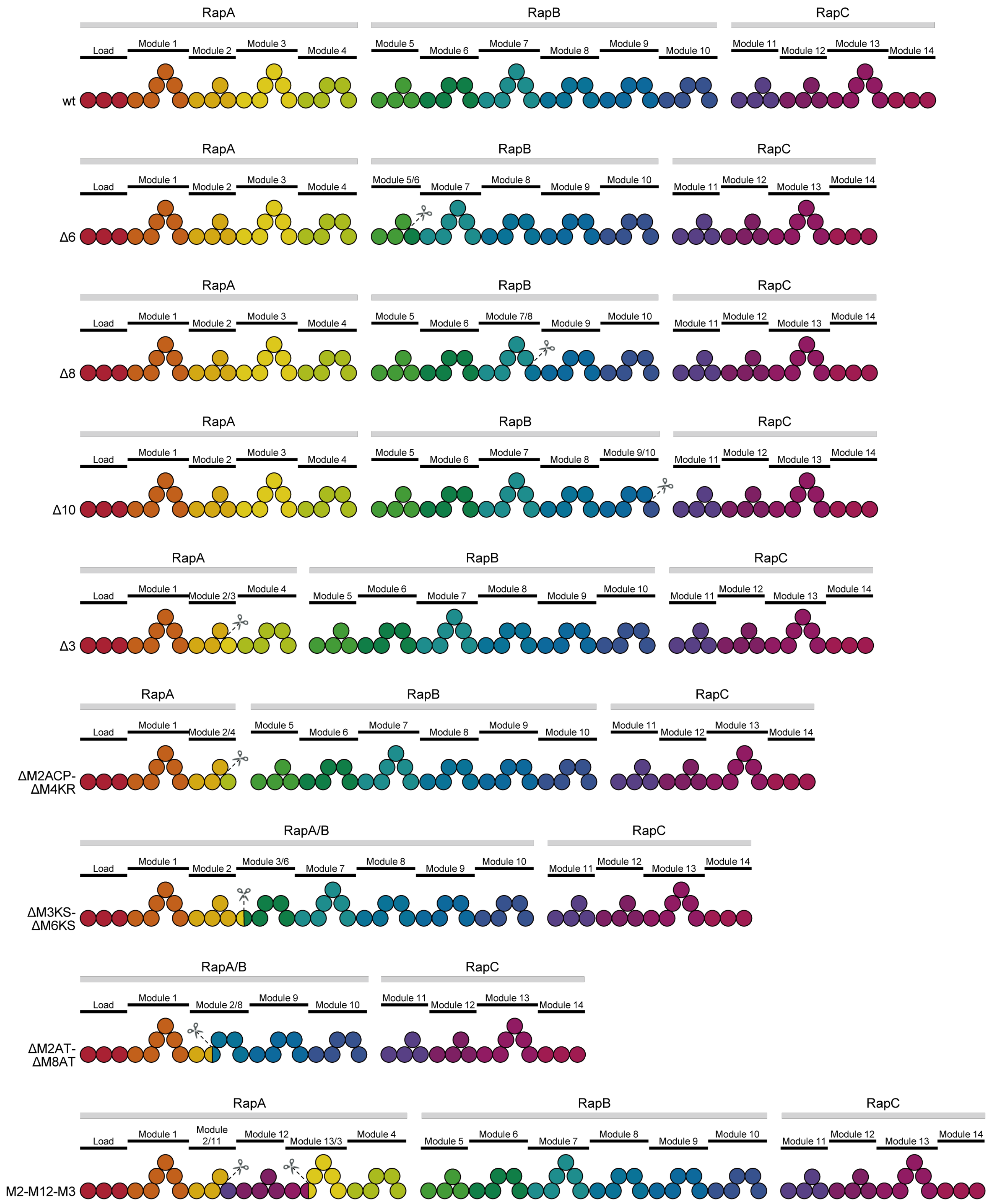

Supplementary Figure 5. Modular alignment of module-deleted and module-inserted rapamycin PKSs. Each domain (KS, AT, DH, ER, KR and ACP) is indicated as a circle, and the circles are coloured by module. Scissors indicate the editing point.

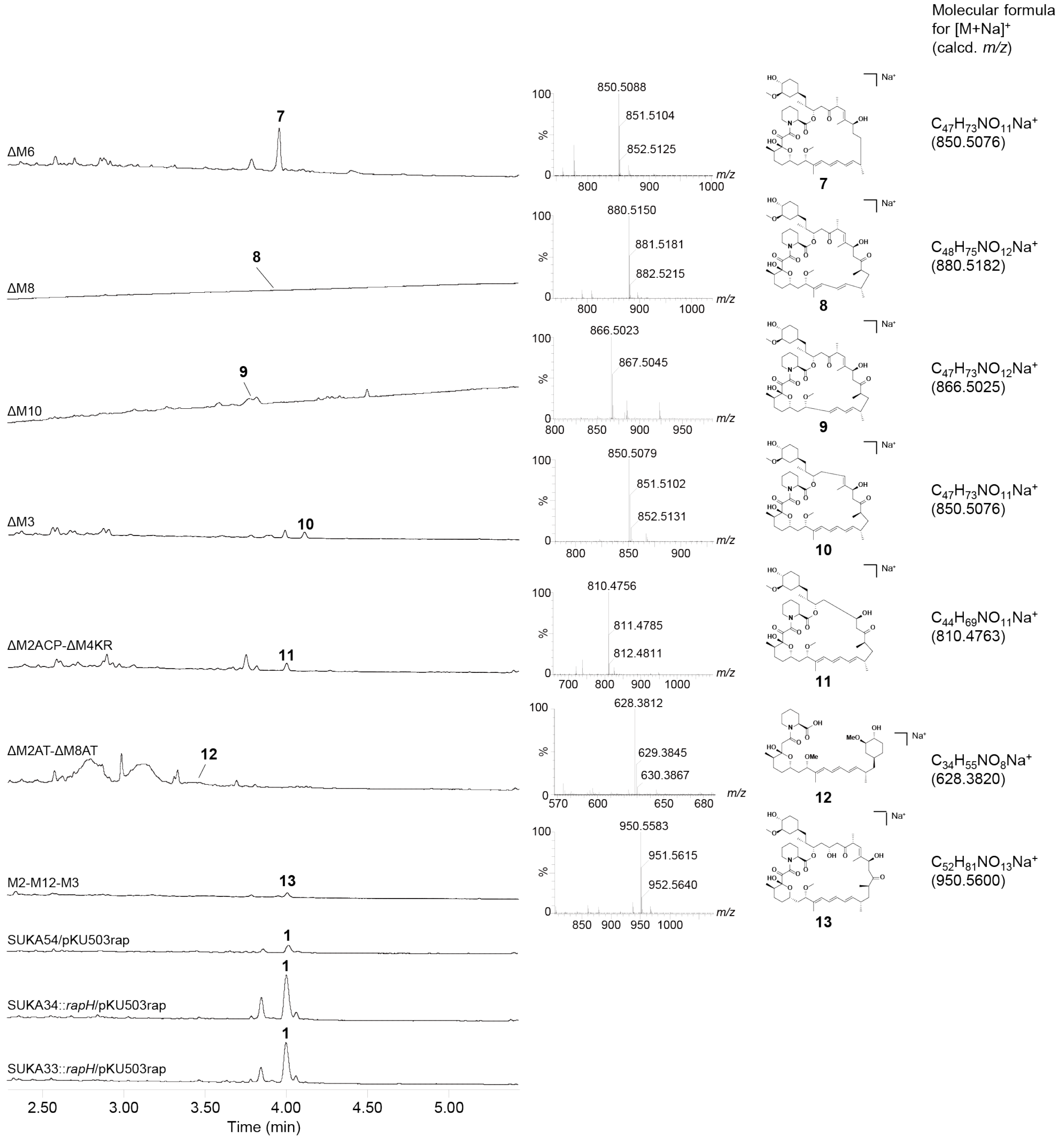

Supplementary Figure 6. Production of module-deleted and module-inserted rapamycin derivatives. Comparative UPLC analysis of the metabolites. UV chromatograms of SUKA33::*rapH*/pKU503rapΔM6, SUKA33::*rapH* /pKU503rapΔM3, SUKA33::*rapH* /pKU503rapΔM2ACP-ΔM4KR, SUKA54/pKU503rapΔM2AT-M8AT, SUKA33::*rapH* /pKU503rap_M2-M12-M3, SUKA54/pKU503rap, SUKA34::*rapH*/pKU503rap and SUKA33::*rapH*/pKU503rap at 280 nm and of SUKA34::*rapH*/pKU503rapΔM8 and SUKA33::*rapH*/pKU503rapΔM10 at 220 nm are shown. The HR-ESI-MS data, the structure of each corresponding derivative, the molecular formulas and the calculated *m/z* values for $[M+Na]^+$ are indicated in the column to the right of each chromatogram. Each chromatogram shown is a representative of at least three biological duplicates.

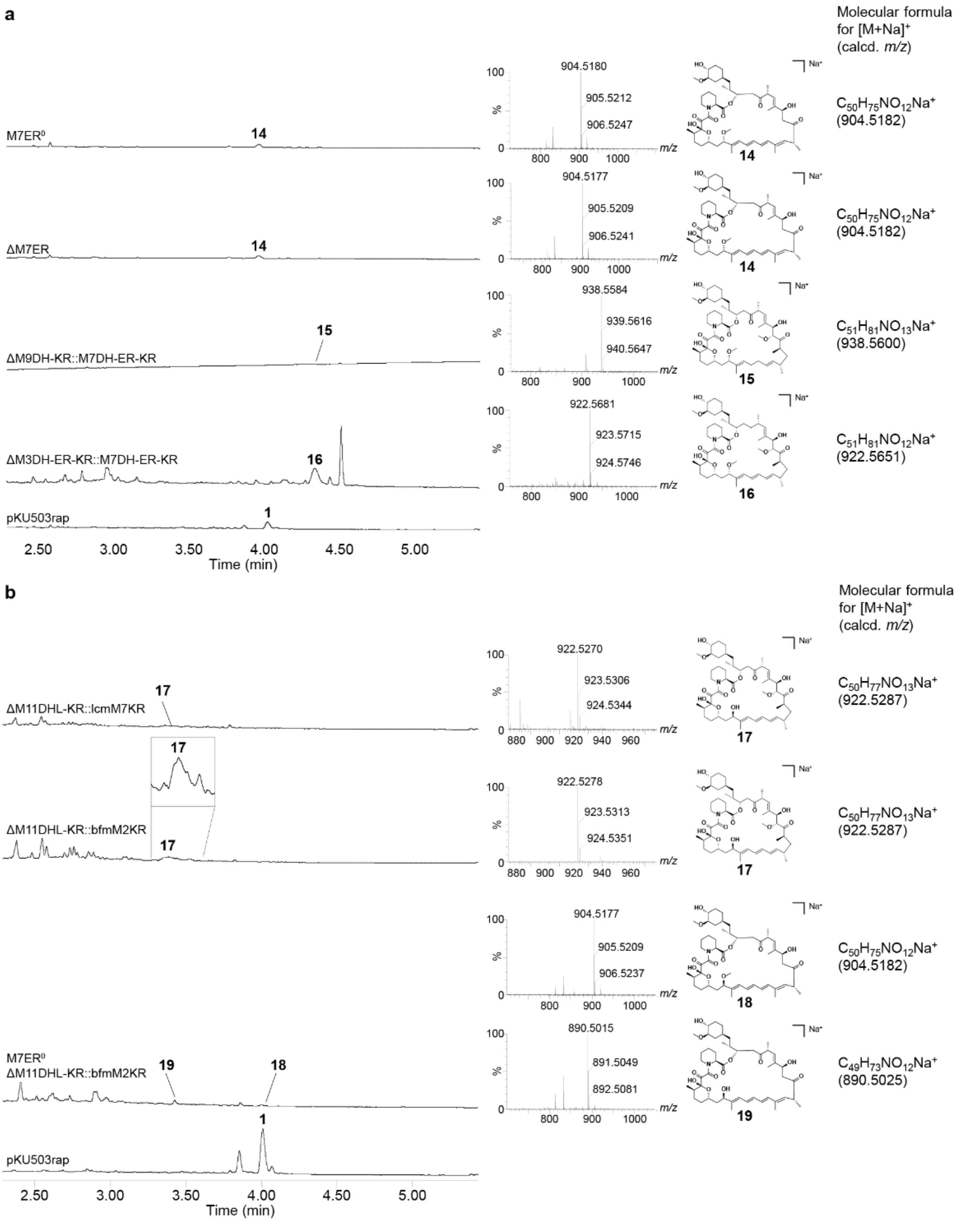

Supplementary Figure 7. Production of reductive loop-manipulated rapamycin derivatives. Comparative UPLC analysis of the metabolites. **a**, Manipulation of reductive loops. **b**, Stereoconversion of the hydroxy group at C-16. UV chromatograms of SUKA54/pKU503rapM7ER^0 and pKU503rapΔM7ER at 310 nm; of SUKA34::*rapH*/pKU503rapΔM9DH-KR::M7DH-ER-KR at 220 nm; and of SUKA34::*rapH*/pKU503rapΔM3DH-ER-KR::M7DH-ER-KR, SUKA54/pKU503rap, SUKA34::*rapH*/pKU503rapΔM11DHL-KR::lcmM7KR, SUKA34::*rapH*/pKU503rapΔM11DHL-

KR::bfmM2KR, SUKA34::*rapH*/pKU503rapM7ER^0_ΔM11DHL-KR::bfmM2KR, and SUKA34::*rapH*/pKU503rap at 280 nm are shown. The HR-ESI-MS data, the structures of each corresponding derivative, the molecular formulas and the calculated *m/z* values for $[M+Na]^+$ are shown in the column to the right of each chromatogram. Each chromatogram shown is a representative of at least three biological duplicates.

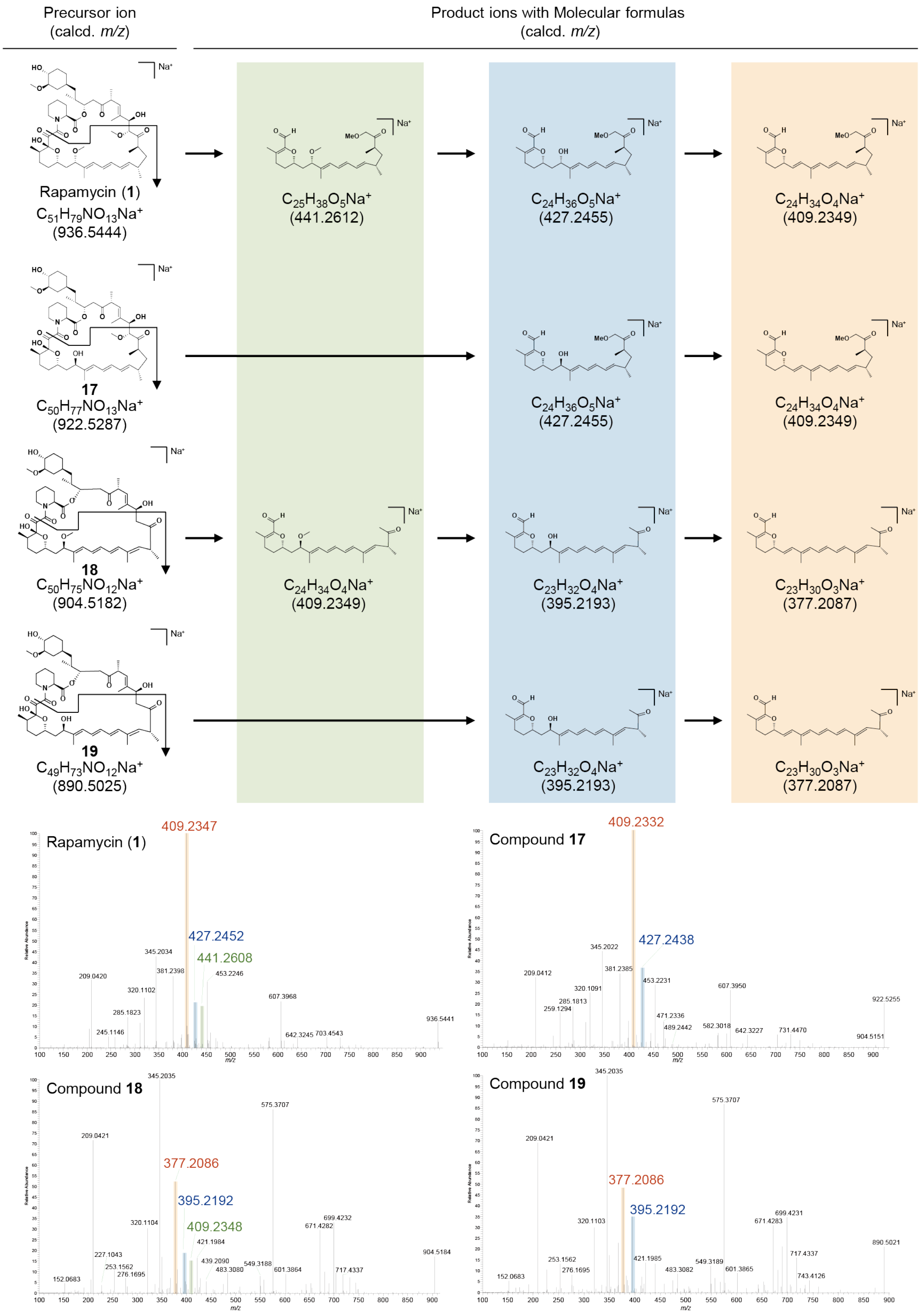

Supplementary Figure 8. MS/MS analysis of stereochemistry-manipulated rapamycin derivatives. Fragmentation patterns of rapamycin (**1**), **17**, **18** and **19**[1], and observed MS/MS spectra of **1**, **17**, **18** and **19** are shown, respectively.

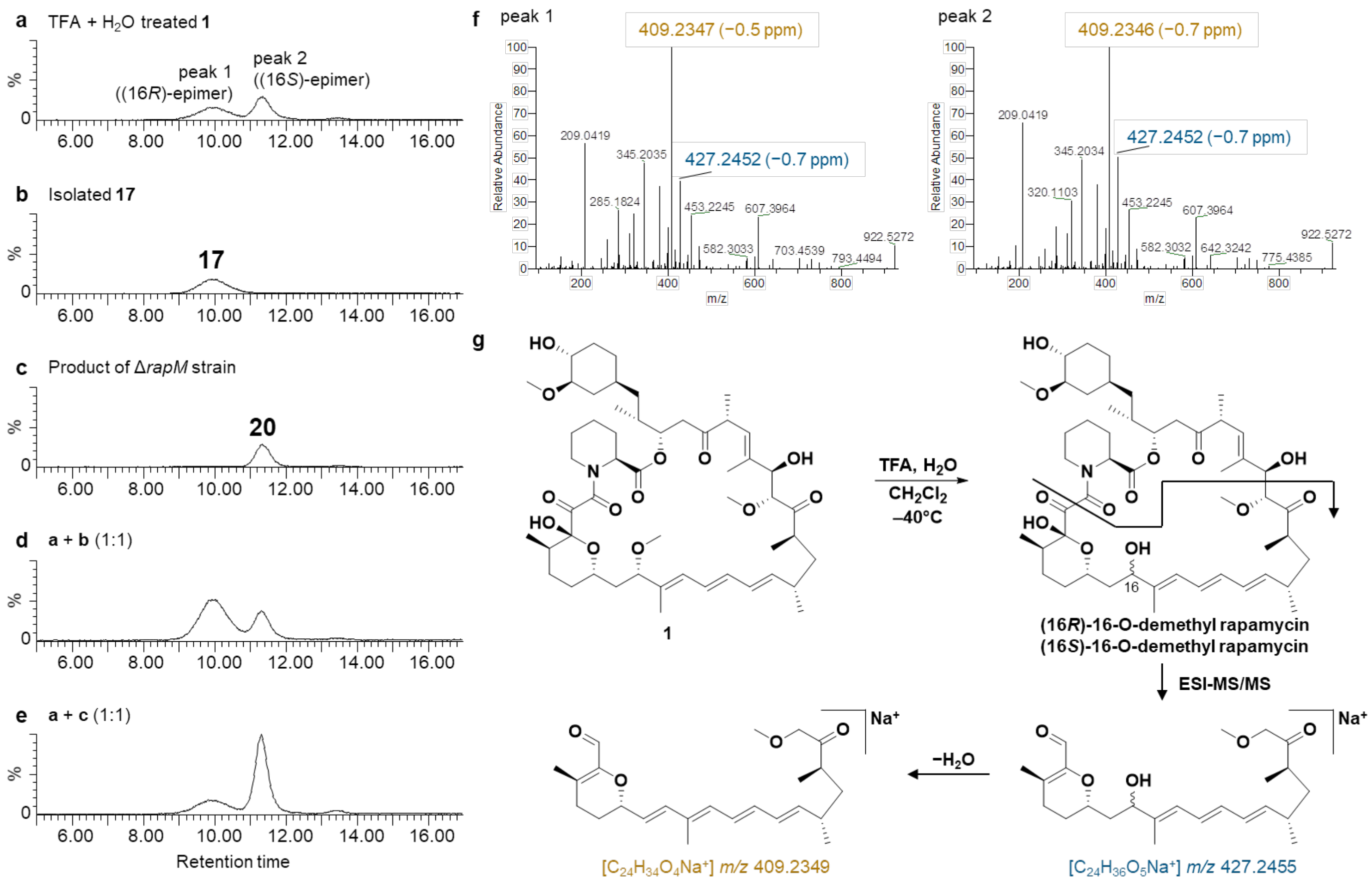

Supplementary Figure 9. Identification of **17** as (16*R*)-16-*O*-demethyl rapamycin. A diastereomeric mixture of 16-*O*-demethyl rapamycins (**a**), **17** (**b**), and **20** (**c**) were analysed by isocratic UPLC (40% acetonitrile/H_2O + 0.1% formic acid) equipped with ESI-MS as well as MS/MS fragmentation analyses. The mass chromatograms of the 1:1 mixture (**d** and **e**) indicate that **17** and **20** are identical to the corresponding compounds of the peaks 1 and 2, respectively. Furthermore, the MS/MS fragmentation patterns of both peak 1 ((*R*)-epimer) and peak 2 ((*S*)-epimer; reported to be less polar than (*R*)-epimer) were same (**f**) and the key fragments, *m/z* 427 and 409, were observed (**g**), which indicate that both diastereomers were simultaneously synthesised as reported[2]. Since it is reported that (*S*)-epimer of the C16 position is less polar than (*R*)-epimer[2], we concluded that **17** is (16*R*)-16-*O*-demethyl rapamycin and **20** is (16*S*)-16-*O*-demethyl rapamycin. Each chromatogram shown is a representative of at least three biological duplicates.

a b c

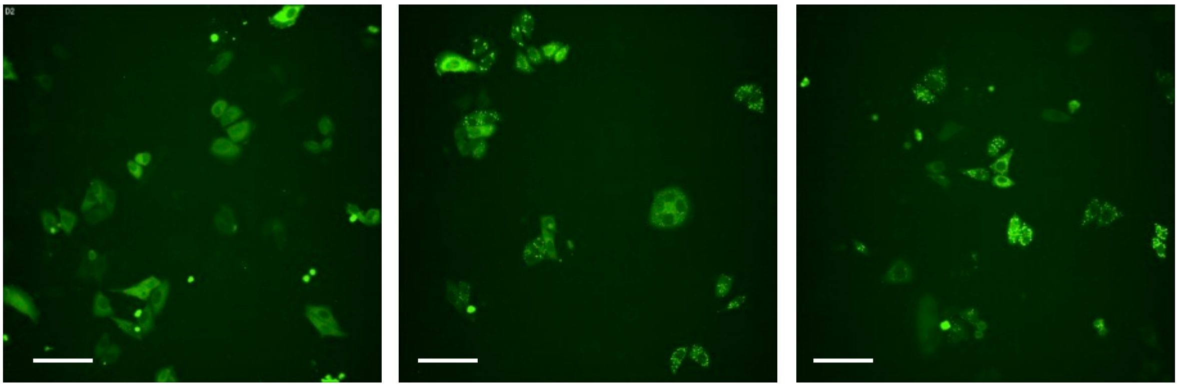

Supplementary Figure 10. Rapamycin and 15 showed potent PPI-inductive activities between mTOR and FKBP. **a**, control. **b**, rapamycin (12 nM). **c**, **15** (12 nM). The experiments were performed six times independently with similar results. Scale bar: 100 µm.

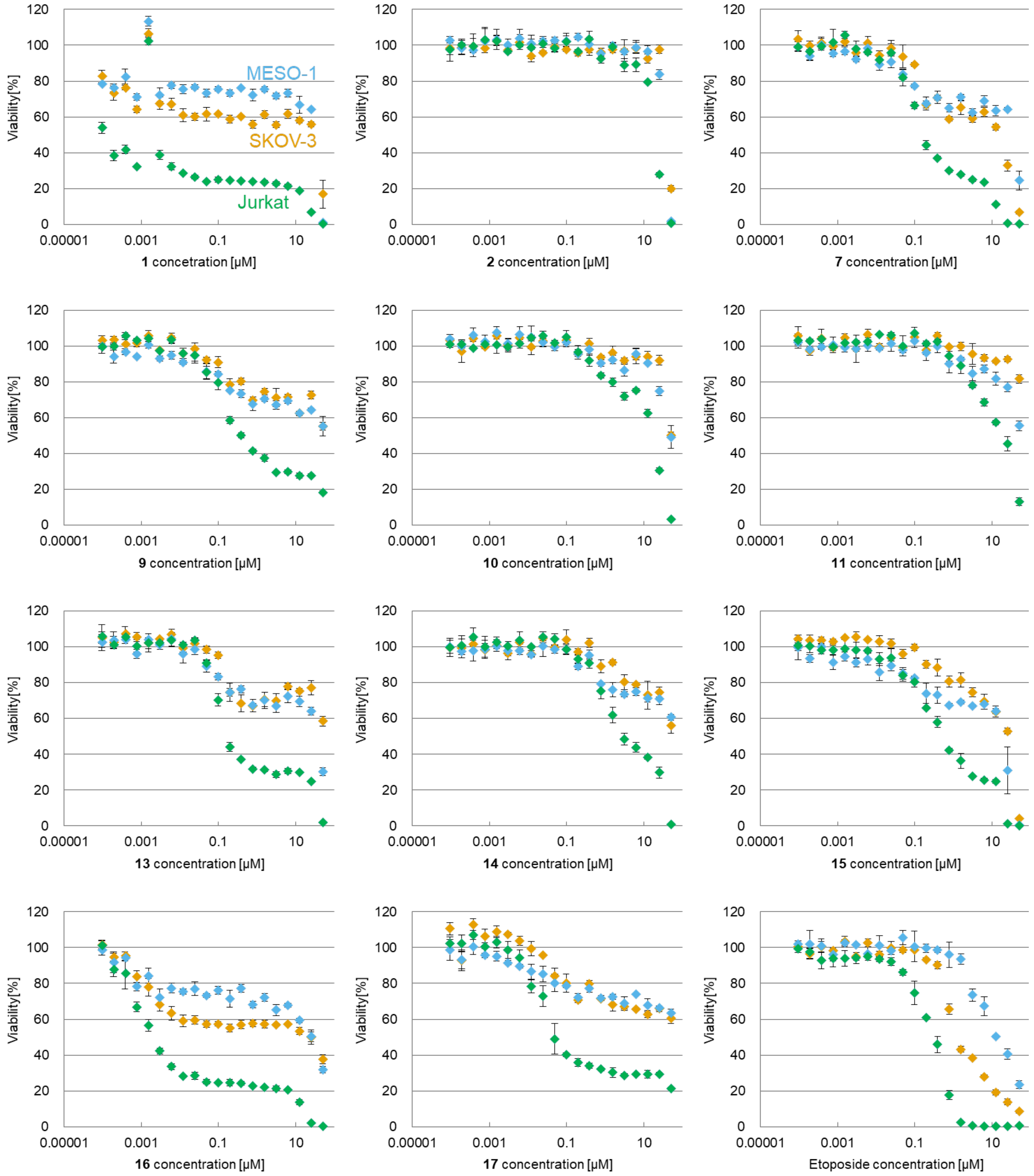

Supplementary Figure 11. Viability curves on the treatment of rapamycin derivatives. Rapamycin (**1**) or its derivatives did not show significant cytotoxic activity but cytostatic effect. Light blue, MESO-1; orange, SKOV-3; green, Jurkat. Each plot with error bars indicates average ± s.d. calculated from three independent biological replicates. Source data are provided as a Source Data file.

Supplementary Table 1. List of oligonucleotides used for "in vitro module editing".

	sgRNA*		Fr. 1†		Fr. 2†		Fr. 3†		Fr. 4†		Fr. 5†		Screening Primers‡	
Name	5’	3’	F	R	F	R	F	R	F	R	F	R	F	R
ΔM9AT::M6ATm	1	2	3	4	5	6							7	8
ΔM4ATm::M8AT	9	10	11	12	13	14							15	16
ΔM6ATm::M8AT	17	18	19	20	21	22							23	16
ΔM7ATm::M8AT	24	25	26	27	28	29							30	16
ΔM8AT::M6ATm	31	32	33	34	35	36	37	38					7	39
ΔM7ATm::M6ATm	24	25	40	41	28	29							30	42
ΔM6	43	44	45	46									47	48
ΔM8	49	50	51	52	53	54							55	56
ΔM10	57	58	59	60									61	62
ΔM3<br>(ISOM-4280)	63	64	65	66	67	68							69	70
ΔM2ACP-ΔM4KR<br>(ISOM-4144)	63	71	65	72	73	74							69	75
ΔM3KS-ΔM6KS<br>(ISOM-4185)	76	77	78	79	80	81							82	83
ΔM2AT-ΔM8AT<br>(ISOM-4193)	63	32	65	84	85	86							69	87
M2-M12-M3<br>(ISOM-4309)	63	88	65	89	90	91	92	93	94	95	96	97	98	99
ΔM7ER	100	101	102	103									104	105
ΔM7ER^{0}	25	106	107	108	109	110							111	112
ΔM9DH-KR::M7DH-ER-KR	113	114	115	116	117	118	119	120					121	122
ΔM11DHL-KR::bfmM2KR	123	124	125	126	127	128	129	130					131	132
ΔM11DHL-KR::lcmM7KR	123	124	125	133	134	135	136	130					137	138
ΔrapM§	139	140											141	142

The oligo nucleotide sequences of the corresponding numbers are listed in Supplementary Table 2. *pKU503rap was digested by Cas9 with the corresponding pair of sgRNAs of which target sequences are listed. To obtain full length oligonucleotide sequence for in vitro sgRNA synthesis, add TTCTAATACGACTCACTATAG to the 5’-end and GTTTTAGAGCTAGA to the 3’-end. †The donor DNA fragments were divided into several small fragments, if necessary, and amplified by overlap PCR. ‡Screening primers were used to select the desired clone after Gibson assembly. §The donor DNA fragment was prepared as a synthetic DNA purchased from GENEWIZ Japan Corp. (Saitama, Japan)

Supplementary Table 2. Sequences of oligonucleotides used in this study.

	Sequence (5’ to 3’)
1	AACCAGTCCTGGCCCGAAGC
2	GACCGGCGGTGTGCAGGTGT
3	GCTGGTGACGGAGAACCAGTCCTGGCCCGAAGCCGGTCGGCCGCGCCGGGCAGGCGTGTC GTCCTTCGGAGTCAGTGGCACTAATGCCCACGTCATCCTGGAGAGCGCACCCCCCGCTCA GCCCGCGGAGG
4	CACCACCGCACCCAGCAACGGATGCCCACCCGCAGCCGAGCGATCCACACCCTCGAC
5	GGGCATCCGTTGCTGGGTGCGGTGGTGGCGTTGCCG
6	GTGTCCGGACTCGTCAGCCTCACCA
7	GTACTGGGTCGAGGGTGTGGATCGCT
8	CCTGCAAACCCTGGAACGCGGGACCGTACTCGTACCCTTCTGCCATCAGCCGGTCGTAGA AGCCCGT
9	ATAACCTTCGCGATCGAACC
10	GCACGTTTCCGCCACCGTCA
11	GCCCTGGCCCGGCACCGACCGGCCCCGCCGGGCAGGCGTGTCGTCCTTCGGGATCAGTGG CACCAACGCCCACGTCATCCTGGAAAGCGCACCCCCCACTCAGCCTGCGGACAACGCGGT GATC
12	ACCCAGCAACGGATGACCGCCCGCGGCCATCCGGTCCGTGCCCTCGAG
13	GGTCATCCGTTGCTGGGTGCGGTGGTGGAGTTGCCTGAAT
14	ACGGTGGGACAGTCGTGTCGGAAACC
15	TTGGCTACGGCGCCGGGTTC
16	CTGGGTCCTGGCCGAAATCACCAT
17	GCCGAACGGCCCCTCCCAGC
18	ATTGGAGTCCGGCAACTCCA
19	ACGGCCTGTCCGCGCCGAACGGCCCCTCCC
20	CGACGGGGCCGGTCGGTGC
21	GCACCGACCGGCCCCGTCGGGCAGGCGTGTCGTCCTTCGGGATCAGTGGCACCAACGCCC ACGTCATCCTGGAAAGCGCACCCCCCACTCAGCCTGCGGACAACGCGGTGATC
22	TCAGCACCACACCATTGGAGTCCGGCAACTCCACGGCCACACCCAGCAACGGATGACCGC CCGCGGCCATCCGGTCCGTGCCCTCGAG
23	GGTATGCCCTGCGGCAGGGG
24	CTGCGCGTTGTTCACAGACT
25	AGGTGCACGCTAGCGGACGA
26	TCGTCCTTGAGAGCGCACCACCCGCCCAGCCTGCGGACAACGCGGTGATC
27	ACCCAGCAACGGATGACCGCCCGCGGCCATCCGGTCCGTGCCCTCGAG
28	GGTCATCCGTTGCTGGGTGTGGCGGTGGAGTTGCCTGAG
29	GACCGACACGGACAGGTGCACGCTAGCGGA
30	GCGCGTCCAACGGCTTCACG
31	CATCCACCTCGTGTGCCGCA
32	ATCGGACTCGGCGACCGCGA
33	CAGGCTGCCCTCAGCAACGCCGGCCTTGCGG
34	GCTCTCCAGGATGACGTGGGCGTTGGTGCCACTGATCCCGAAGGACGACACGCCTGCCCG GCGCGCT
35	GGCACCAACGCCCACGTCATCCTGGAGAGCGCACCCCCCGCTCAGCCCGCGGAGG
36	CCGCCTCACCCAGCAGCGGATGGCCACCCGCAGCCGAGCGATCCACACCCTCGAC

37	GGCCATCCGCTGCTGGGTGAGGCGGTGGCGG
38	GTCACCGCCCGGCGTCCGGAATCATCGGACTCGGCGACCGCG
39	GCCTGGGCAGGTGGCCACGCACTG
40	TCGTCCTTGAGAGCGCACCACCCGCCCAGCCCGCGGAGG
41	ACCCAGCAACGGATGACCACCCGCAGCCGAGCGATCCACACCCTCGAC
42	GTGTCACCGCGAGCGTTGATGCA
43	AGCGGCTGGAGACCGTATTC
44	CAGCAACGCCGGAACCTCCG
45	TGTCGTTGAGTCCCTGAGCGCGCAGCGGCTGGAGACCGTATTCC
46	ACCGGGCGACGCAACGAACGCAGCAACGCCGGAACCTCCGCGTCCCGTACCGGCTCCATC GGCGCGGCCACCAGAACCGGTTCACTGTGGCGTGACGCGT
47	GATCGTGCAGAGCTGGCGCGAGTGT
48	GGCGTCGGCGTGCCCCAGGACCACG
49	CGTTGGCGTCGAGTTGCTGA
50	ACCGGGCGGCCAGGGCACCT
51	GTGCCCGCCACATCGGCAAGATCGTCC
52	GCGCCAGAGCGGCCAGCCACTGCACCGACGAGCCG
53	GTGGCTGGCCGCTCTGGCGCCGGAGGAGCGGGCGAAGGCACTGG
54	CTCGCCGGTGAACAGCTCCTCCAACCGGGCGGCCAGGGCAC
55	GACGCCAGCGGCCTGACCGCCGAAA
56	CTTCCCGGCCGCATCCGGATCCGAG
57	CCGCTGGTGTGCTCGGCAGT
58	GGCGCAGTGGCTGGTCGGCT
59	GTTCGTCATGTATTCCTCCGCCGCT
60	ACCACCTTCAGCAGTGCCTTCGCCCGCTCCTCCGGCGCCAGGGCGGCCAGCCACCGCGCT CCACCGGCGGC
61	CTGGGTCCGGCACGTTTCCGCCGT
62	GAACCCTCGGGTTCATGAGGTCGCC
63	GCACTCCCCACACAGCCTGC
64	CGACGCACGCAGCATTCCGG
65	ACGCCCACGTCATCCTGGAAAGCGCACTCCCCACACAGCCTGCGGGCAACACA
66	GCCTTCTCCCGCTCGGCCGGTGCCAGGGCGGCCAGCCACCGCGCTCCACCGGCGGC
67	GTGGCTGGCCGCCCTGGCACCGGCCGAGCGGGAGAAGGCG
68	AGCGAATCCACACCCAGATCCTTGAACGCGCCAGCCGCCGGAATGCTGCGT
69	CTGTTGGTGGAGCGGCTTTCCGATGCCCGT
70	AATACGACTTCCCCGCCGCCTCCGGATCAGA
71	AACGGGCGAAGGCGTTGTTG
72	TGCCGGGACTTCCGCGTCCCGTACCGGCTCCATCGCCGCGGCCACCAGAACCGGTTCACTG
73	GCGGCGATGGAGCCGGTACGGGACGCGGAAGTCCCGGCGCTGCTC
74	GCGGCGCTGTCACACACCACCCTCAA
75	CCGCGGTCACGGGAATGCTGTCGAT
76	ATCCCTGAACACGGCGGCTA

77	GTCCACGAACTCGCGCAACG
78	ACGGTTCTGGGACACGCCGACACCAGCACCGTCTCCGTAG
79	CTGCTGGGAGGGGCCGTTCGGCGCGGTCAGACCGTTG
80	CCGCGCCGAACGGCCCCTCCCAGCAGG
81	TCCAGAACCGTGAACAGGTCCCAGTCCACGAACTCGCGCAACGCG
82	ATTGCTCGGCGGGCCGCTGCCGCCGGTGGAGCGCG
83	GGTCAGCACCACACCATTGGAGTCC
84	ACCGGTCGGCACCCAGCTCGACGAACACCGCA
85	GTGTTCGTCGAGCTGGGTGCCGACCGGTCACTGGCCCGCCT
86	GTCACCGCCCGGCGTCCGGAATCATCGGACTCGGCGACCGCG
87	CGCGTGATCAGCCAGCCACGGGTGC
88	CGTGGCCACCAGCCCAGGCC
89	CCACCGGCGGCAGCGGCCCGCCGAGCAATC
90	ATTGCTCGGCGGGCCGCTGCCGCCGGTGGA
91	GCCCGAACAGAGCCACCTGCATT
92	AATGCAGGTGGCTCTGTTCGGGCTGCTGGAATCGTGGGGTGTACGA
93	TGCGGCGACCAGAATCGGGTTG
94	CAACCCGATTCTGGTCGCCGCA
95	TGGAAGGCGTAGGTCGGAAGGTCCAGTACCCGGGTTGTGGT
96	CCACAACCCGGGTACTGGACCTTCCGACCTACGCCTTCCAGCACCAGCGGTACTGGCTCAG
97	CCAGCCGGTCGTAGAAGTCGGCCACGCT
98	CACCGTGATGGCCACACCACAAAGC
99	GCGGGACCGTACTCGGTACCTGCCG
100	CCTCAGCAACTCGACGCCAA
101	CGACAGTCGCGGCCAGTTGC
102	GGTGGAATGCGACGACAACCTCACCCTCCAGCAACTGGCCGCGACTGTCGGATTGGATGA GCCGCGGCTGCGGA
103	CGATCCCCGCCAGGACACCTGAACCACCGGTGACCAGAACCGTGCCGTCCGGATCCCACA CCTTTTCTGA
104	GCTAGCCCCGCTGGATCCGCCG
105	ACCACGCCGGATCCGGTCACGGTCCGTGTCAGTCATTTCG
106	CCGTTGGCGTCGAGTTGCTG
107	CGATGAGCTGGTGATCGAAACCCCGCTGCTGCTGCCGTCGTCCGCTA
108	CCATGCCGACAGGACTAGCGGCGGCGTGGATCAGCACGGAC
109	GCCGCCGCTAGTCCTGTCGGCATGGCAGCCACCCAGATC
110	AACCACCGGTGACCAGAACCGTGCCGTTGGCGTCGAGTTGCTGAG
111	GGAATGCGACGACAACCTCACCCTCCAG
112	GTGGCTGCCATGCCGACAGGACTA
113	GGCCTGACCTATCACGCACC
114	CTACTTACGGCCAGGGCCGC
115	TGGAGGAGTTCCGGGCGGTCGCTC
116	GTCCGCACCCTCGGCCCAATACCGCTGCTGCTGGAAGGCGTACGTCGGAAGATCCAGCAC CCGGGTTA

117	CAGCAGCGGTATTGGGCCGAGGGTGCGGACCGGTCGGTTGCGGGCGGTCATCCGTTGCTG GGTGTGGCGGTGGAGTTGCCTGAG
118	TGCCAGGGCGGCCAGCCACTGCACCGACGAGCCGCCGGTA
119	TCGTCGGTGCAGTGGCTGGCCGCCCTGGCACCGGCCGAGCGGGAGAAGGCACTGCTGAAG CTGGTGTCTGACG
120	CGTTCGACTTCAGCGATCCCAGCAGCAGCGGCTCCC
121	CGGTTACGCCCAGCCGGCTTTGTT
122	TCGCCGACCGACACGGACAGGTGCACGCTAGCGGACGA
123	GGTCGATGTTCGAACACCGT
124	CTTCGGGGCCACCGCCAGTT
125	CGCGTCAACACTGGCGATGACACGGTCGATGTTCGAACACCGTGGG
126	CGGCCCGCGGCCAGTACCGCTGGTGCTGGAAGGCGTAC
127	GCGGTACTGGCCGCGGGCCGCGGCGGACCCGGCCC
128	GGGCGGCCAGCCGCTCCGCCCAGGACGGGTCGCCGCCCGGGG
129	GGCGGAGCGGCTGGCCGCCCTGGCACCGGCCGAGCGGGAGAAG
130	AAGAAGTACGACACCCGGCCGGACAGGACACTGAC
131	GGCTCACCTGTACGTGAACGGCGTGAGT
132	GACGTCGCAGGCCGCCAGGGTGGTG
133	CGGGTTCGAGCCAGTACCGCTGGTGCTGGAAGGCGTAC
134	GCGGTACTGGCTCGAACCCGCGCCCGCCCCGGCCCGCCGGACGGA
135	GGGCGGCCAGGTCGGCGAGGCCGGACGCCACGTG
136	CCTCGCCGACCTGGCCGCCCTGGCACCGGCCGAGCGGGAGAAG
137	CATGGCCGCTGGTGATCAGGTGATG
138	CAGCACGAGGTGTTCGGCGCCGTTG
139	TGTAGTGATGACCGACGTAG
140	CATCATGCTGATGGACCCTC
141	CGATCCGTACTCGCTGTAGTGATG
142	GTGGATCTCGAGTCGCTCCATTT

Supplementary Table 3. Yield of rapamycin and its derivatives produced in this study.

Compound	Yield (mg L^{-1})
Rapamycin (**1**)	8.6
**2**	1.1
**3**	0.14†
**4**	0.14†
**5**	0.52†
**6**	0.26
**7**	1.5
**8***	N.D.
**9**	4.0
**10**	0.78
**11**	1.2
**12**	2.1
**13**	0.40
**14**	1.2
**15**	1.1
**16**	1.5
**17**	0.37
**18***	N.D.
**19**	0.23†

Otherwise mentioned, yields were calculated from the amount of each isolated compound. N.D., not determined. *Only detected by MS. †Deduced from the peak area of UV absorption at 280 nm.

NMR data

Supplementary Data 1. Chemical structure and 2D NMR key correlations for compound **2**.

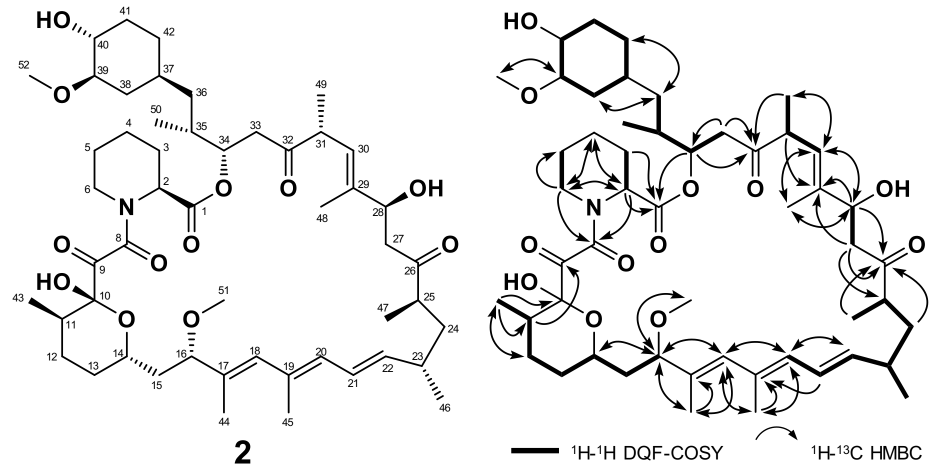

Supplementary Data 2. NMR data for compound **2** in Acetone-d_6 at 600 MHz for ^{1}H and 150 MHz for ^{13}C.

Position	δ_C	δ_H (multiplicity, J in Hz)	Position	δ_C	δ_H (multiplicity, J in Hz)
1	170.1	-	27	49.4	2.68 (m), 2.48 (m)
2	52.3	5.16 (m)	28	72.3	4.37 (m)
3	27.6	2.30 (m), 1.73 (m)	29	141.3	-
4	21.6	1.77 (m), 1.46 (m)	30	124.2	5.38 (d, 9.0)
5	25.8	1.66 (m), 1.44 (m)	31	46.9	3.39(m)
6	44.8	3.55 (m), 3.26 (m)	32	208.6	-
7	-	-	33	41.8	2.74 (m)
8	168.0	-	34	75.5	5.27 (m)
9	196.9	-	35	34.6	1.92 (m)
10	99.7	-	36	39.8	1.73 (m), 1.27 (m)
11	35.3	2.10 (m)	37	34.1	1.43 (m)
12	28.0	1.62 (m)	38	36.0	2.07 (m), 0.67 (m)
13	31.7	1.34 (m), 1.81 (m)	39	85.4	2.90 (m)
14	68.2	4.06 (m)	40	74.6	3.29 (m)
15	40.7	1.84 (m), 1.29 (m)	41	33.2	1.88 (m), 1.28 (m)
16	85.5	3.66 (m)	42	32.5	0.99 (m)
17	135.7	-	43	15.9	0.91 (d, 6.9)
18	133.5	6.01 (s)	44	12.5	1.76 (s)
19	134.0	-	45	17.2	1.97 (s)
20	131.7	5.97 (d, 10.8)	46	22.4	1.02 (d, 7.0)
21	124.6	6.39 (dd, 15.0, 10.8)	47	17.5	1.01 (d, 6.9)
22	141.0	5.32 (dd, 15.0, 9.36)	48	13.4	1.65 (s)
23	38.9	2.29 (m)	49	16.5	1.05 (d, 6.9)
24	41.4	1.78 (m), 1.30 (m)	50	16.5	0.92 (d, 6.5)
25	46.3	2.59 (m)	51	55.9	3.14 (s)
26	213.7	-	52	57.2	3.36 (s)

Supplementary Data 3. ^{1}H NMR spectrum (Acetone-d_6, 600 MHz) of compound **2**.

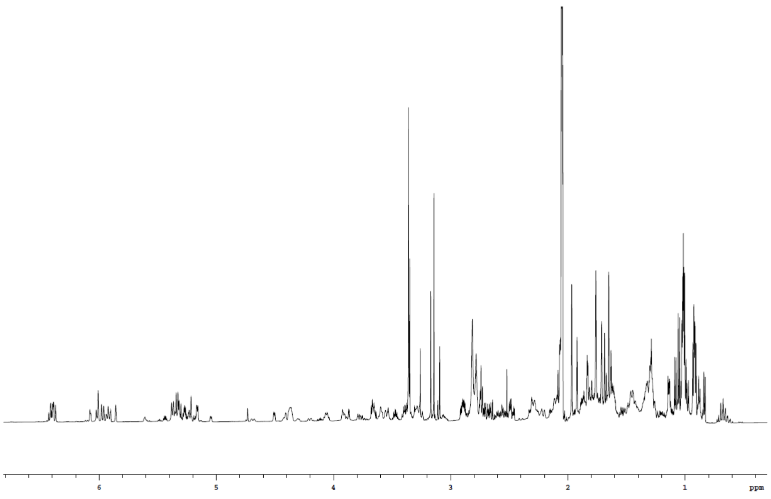

Supplementary Data 4. ^{13}C NMR spectrum (Acetone-d_6, 150 MHz) of compound **2**.

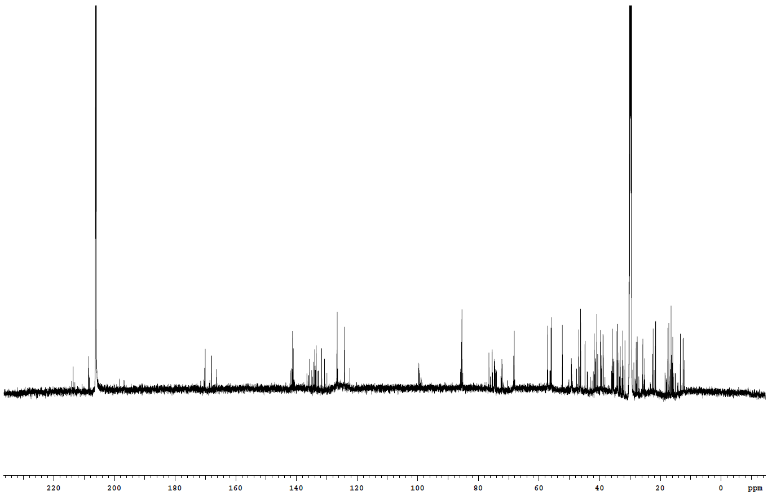

Supplementary Data 5. DQF-COSY NMR spectrum (Acetone-d_6, 600 MHz) of compound **2**.

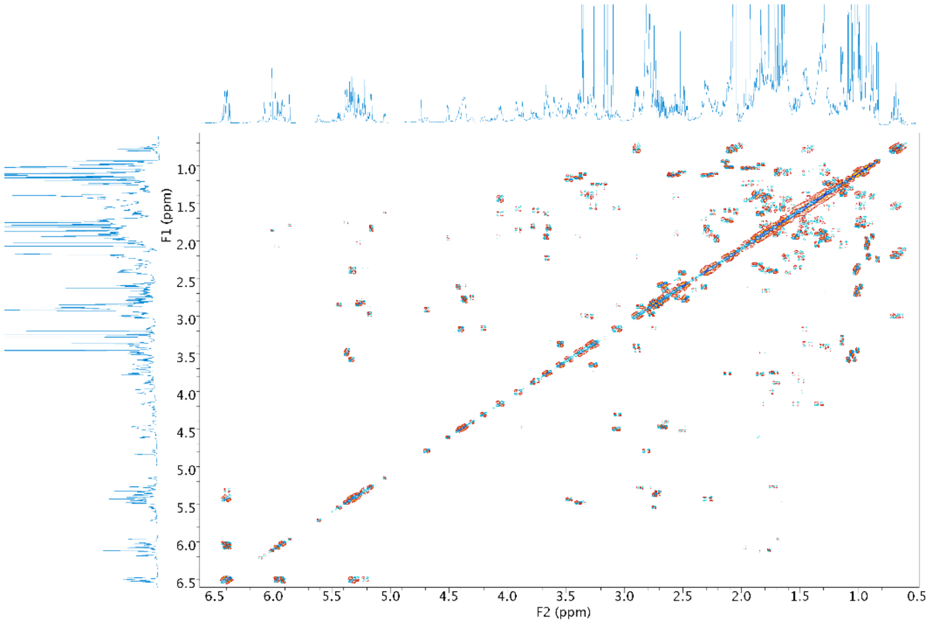

Supplementary Data 6. HSQC NMR spectrum (Acetone-d_6, 600 MHz) of compound **2**.

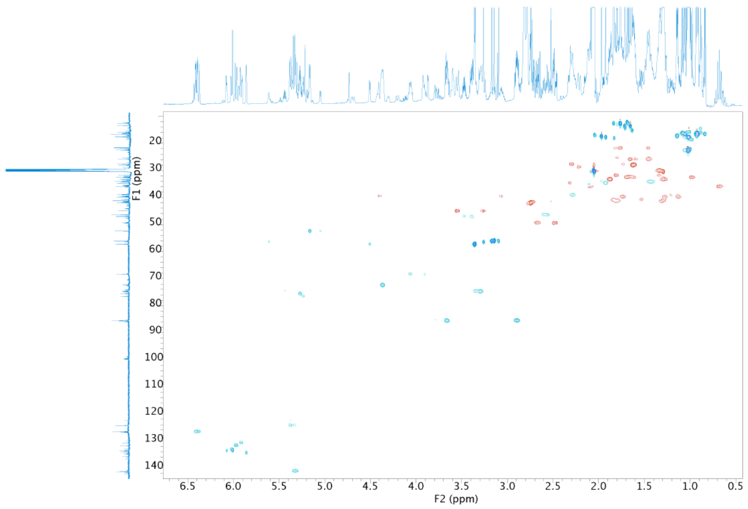

Supplementary Data 7. HMBC NMR spectrum (Acetone-d_6, 600 MHz) of compound **2**.

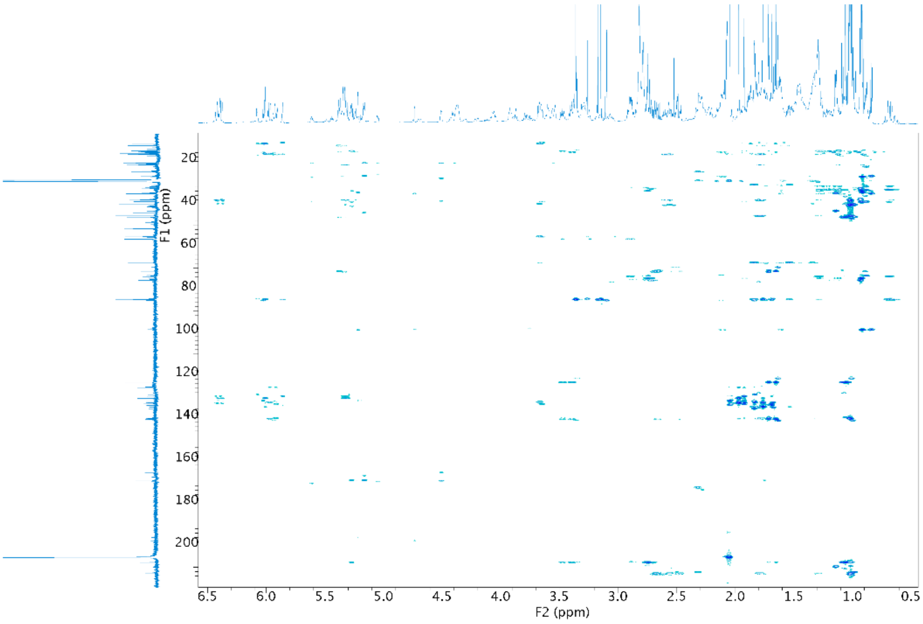

Supplementary Data 8. Chemical structure and 2D NMR key correlations for compound **6**.

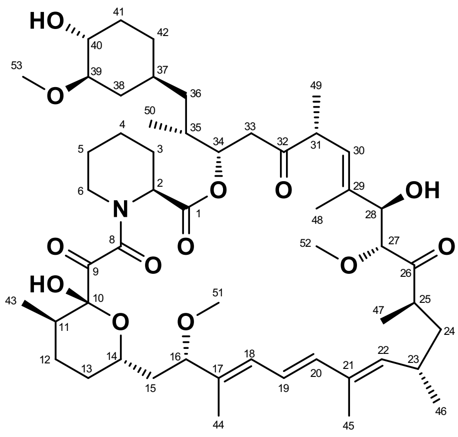

6

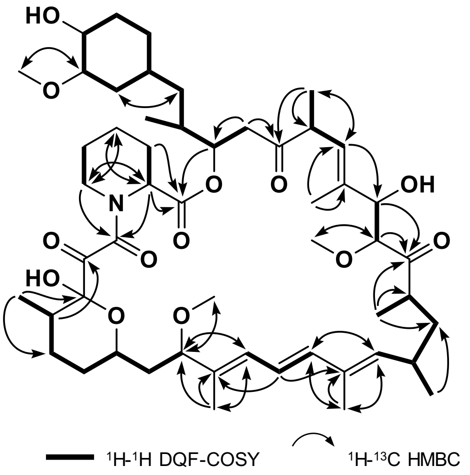

Supplementary Data 9. NMR data for compound **6** in Acetone-d_6 at 500 MHz for ^{1}H and 150 MHz for ^{13}C.

Position	δ_C	δ_H (multiplicity, J in Hz)	Position	δ_C	δ_H (multiplicity, J in Hz)
1	170.2	-	28	77.7	4.18 (m)
2	52.1	5.18 (m)	29	138.1	-
3	27.5	2.29 (m), 1.72 (m)	30	127.1	5.29 (d, 9.6)
4	21.5	1.76 (m), 1.54 (m)	31	46.8	3.42 (m)
5	25.9	1.43 (m), 1.41 (m)	32	208.2	-
6	44.9	3.51 (m), 3.35 (m)	33	41.4	2.87 (m), 2.57 (m)
7	-	-	34	75.5	5.14 (m)
8	167.9	-	35	34.6	1.90 (m)
9	197.1	-	36	39.7	1.24 (m), 1.12 (m)
10	99.7	-	37	34.0	1.42 (m)
11	35.4	2.12 (m)	38	36.0	2.08 (m), 0.68 (m)
12	27.8	1.61 (m)	39	85.4	2.90 (m)
13	31.3	1.82 (m), 1.34 (m)	40	74.8	3.29 (m)
14	68.1	4.07 (m)	41	33.2	1.86 (m), 1.27 (m)
15	40.6	1.92 (m), 1.47 (m)	42	32.4	0.98 (m)
16	84.5	3.71 (m)	43	16.5	0.88 (d, 7.0)
17	137.5	-	44	10.6	1.71 (s)
18	129.8	6.15 (d, 10.7)	45	13.0	1.87 (s)
19	123.3	6.46 (dd, 15.2, 10.7)	46	21.9	1.00 (d, 6.6)
20	138.8	6.36 (d, 15.2)	47	14.4	0.96 (d, 6.6)
21	134.8	-	48	13.7	1.86 (d, 7.0)
22	139.9	5.34 (d, 10.0)	49	15.8	0.97 (d, 6.8)
23	31.5	2.72 (m)	50	15.8	0.90 (d, 6.8)
24	41.3	1.58 (m), 1.21 (m)	51	56.0	3.11 (s)
25	41.6	2.64 (m)	52	58.4	3.29 (s)
26	212.6	-	53	57.2	3.36 (s)
27	86.6	3.97 (m)			

Supplementary Data 10 ^{1}H NMR spectrum (Acetone-d_6, 500 MHz) of compound **6**.

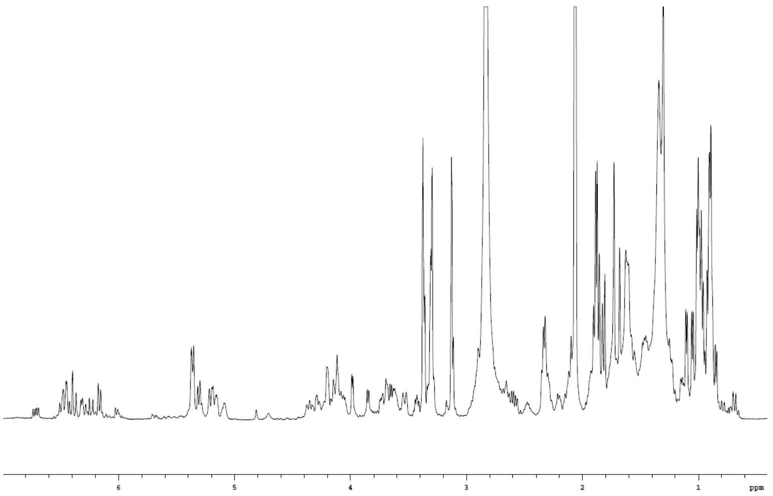

Supplementary Data 11. ^{13}C NMR spectrum (Acetone-d_6, 150 MHz) of compound **6**.

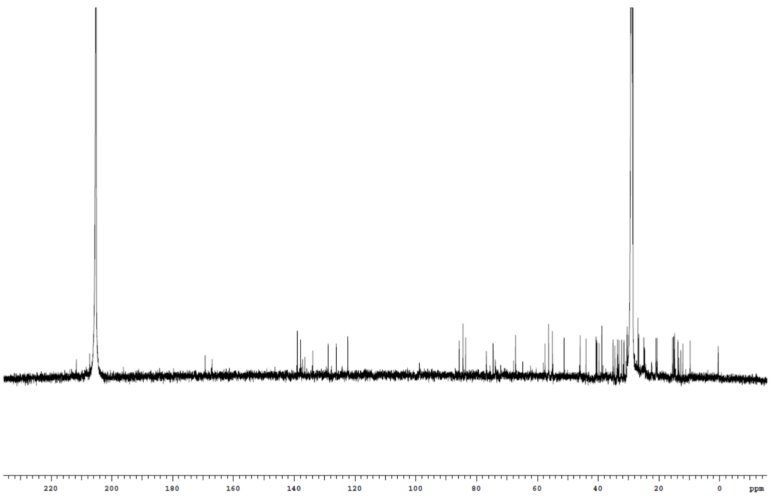

Supplementary Data 12. DQF-COSY NMR spectrum (Acetone-d_6, 500 MHz) of compound **6**.

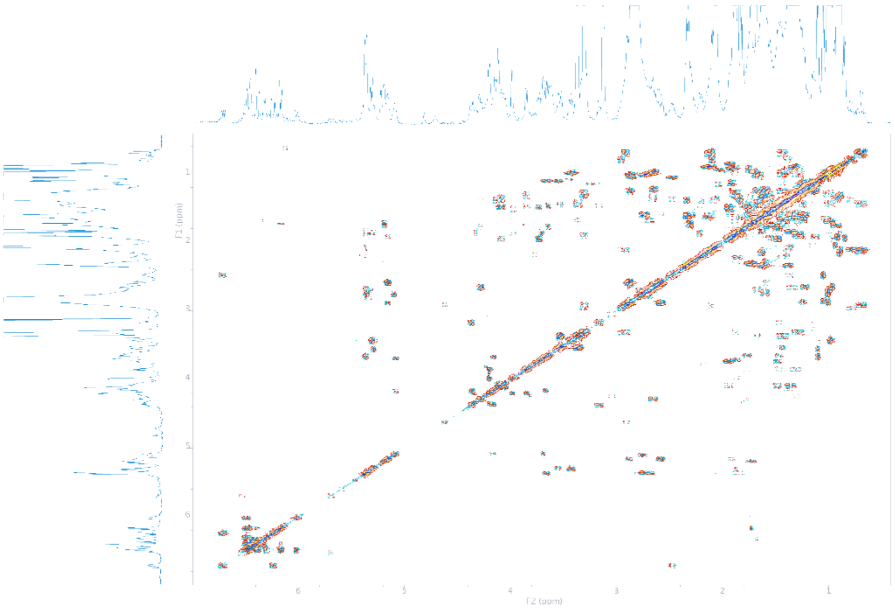

Supplementary Data 13. HSQC NMR spectrum (Acetone-d_6, 600 MHz) of compound **6**.

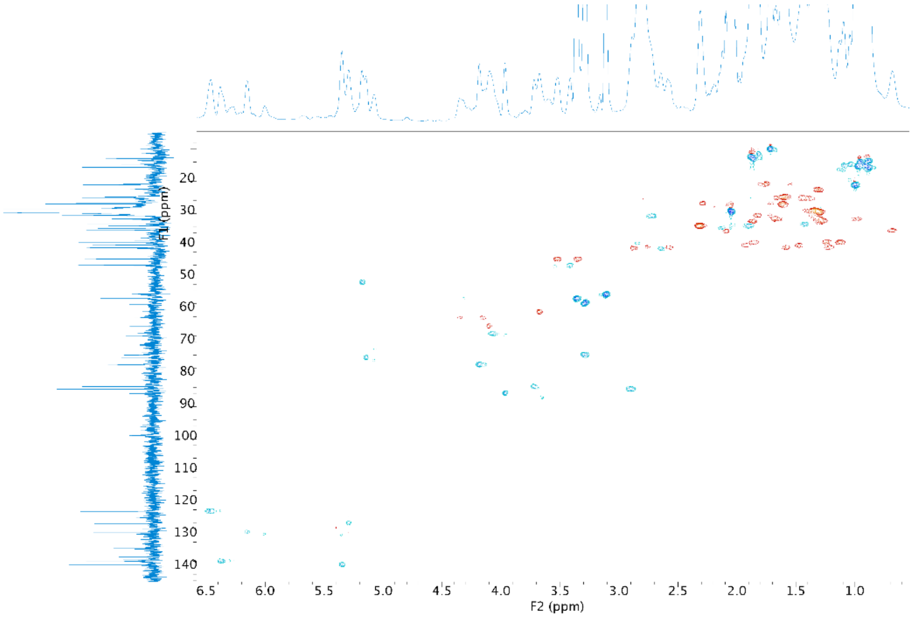

Supplementary Data 14. HMBC NMR spectrum (Acetone-d_6, 600 MHz) of compound **6**.

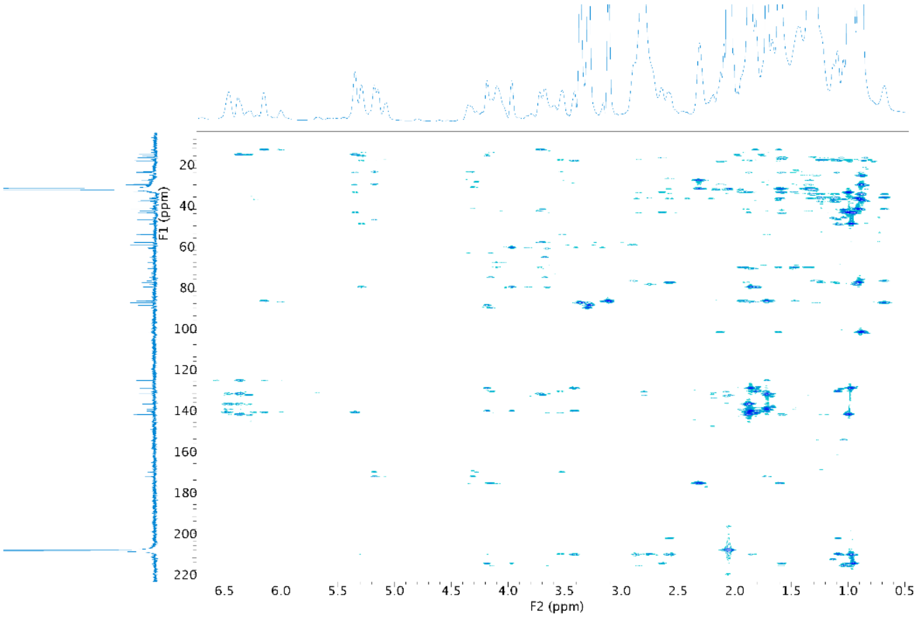

Supplementary Data 15. Chemical structure and 2D NMR key correlations for compound **7**.

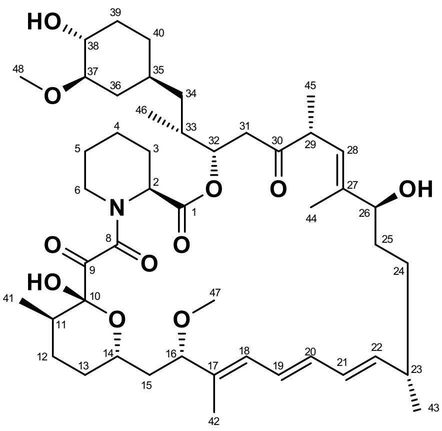

7

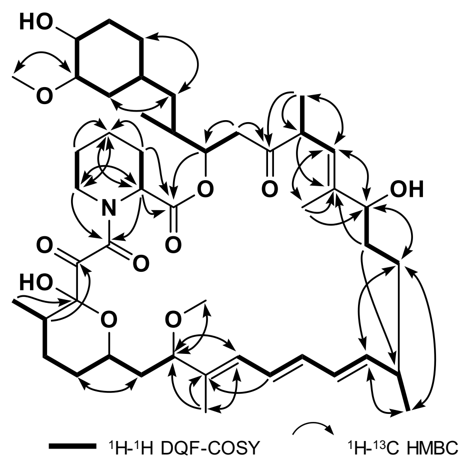

Supplementary Data 16. NMR data for compound **7** in Acetone-d_6 at 600 MHz for ^1H and 150 MHz for ^{13}C.

Position	δ_C	δ_H (multiplicity, J in Hz)	Position	δ_C	δ_H (multiplicity, J in Hz)
1	170.0	-	25	34.7	1.54 (m), 1.42 (m)
2	52.6	5.19 (m)	26	78.6	3.91 (m)
3	27.3	2.28 (m), 1.78 (m)	27	142.9	-
4	21.7	1.79 (m), 1.48 (m)	28	125.6	5.08 (d, 9.0)
5	25.7	1.71 (m), 1.48 (m)	29	46.6	3.51 (m)
6	44.9	3.59 (m), 3.33 (m)	30	208.3	-
7	-	-	31	42.0	2.86 (m), 2.69 (m)
8	167.8	-	32	75.9	5.17 (m)
9	208.6	-	33	34.3	1.96 (m)
10	99.9	-	34	39.4	1.22 (m), 1.12 (m)
11	35.1	2.09 (m)	35	34.0	1.46 (m)
12	27.7	1.59 (m)	36	35.9	2.10 (m), 0.71 (m)
13	31.6	1.72 (m), 1.34 (m)	37	85.5	2.92 (m)
14	67.9	3.92 (m)	38	74.8	3.33 (m)
15	40.6	1.84 (m), 1.60 (m)	39	33.3	1.90 (m), 1.30 (m)
16	84.7	3.71 (m)	40	32.6	1.02 (m)
17	137.1	-	41	16.3	0.87 (d, 6.5)
18	129.8	6.06 (d, 10.8)	42	10.5	1.67 (s)
19	126.9	6.41 (dd, 15.0, 11.0)	43	21.7	1.03 (d, 6.5)
20	134.8	6.31 (dd, 15.0, 10.6)	44	11.3	1.71 (s)
21	130.4	6.14 (dd, 14.5, 10.6)	45	16.3	1.07 (d, 6.7)
22	142.1	5.51 (dd, 14.5, 7.8)	46	16.0	0.92 (d, 6.7)
23	38.5	2.11 (m)	47	56.1	3.11 (s)
24	35.2	1.65 (m), 1.07 (m)	48	57.1	3.38 (s)

Supplementary Data 17. ^{1}H NMR spectrum (Acetone-d_6, 500 MHz) of compound **7**.

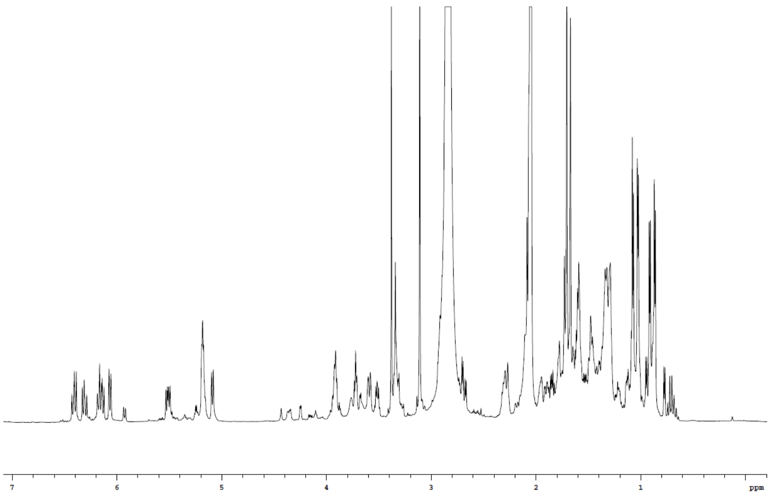

Supplementary Data 18. ^{13}C NMR spectrum (Acetone-d_6, 150 MHz) of compound **7**.

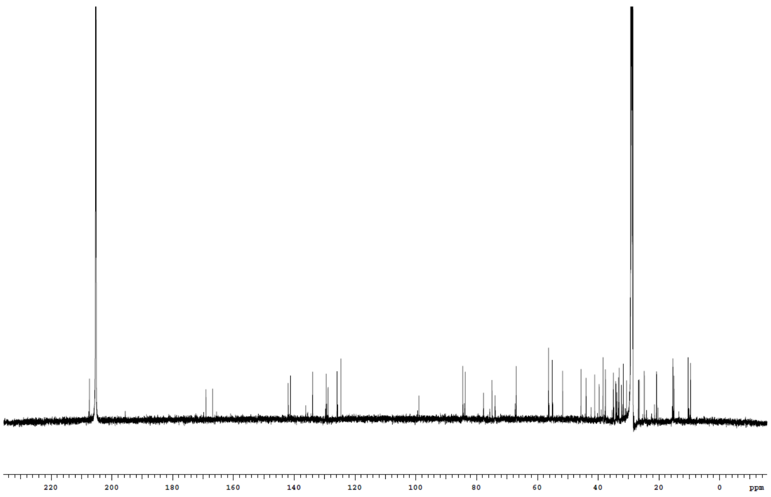

Supplementary Data 19. DQF-COSY NMR spectrum (Acetone-d_6, 600 MHz) of compound **7**.

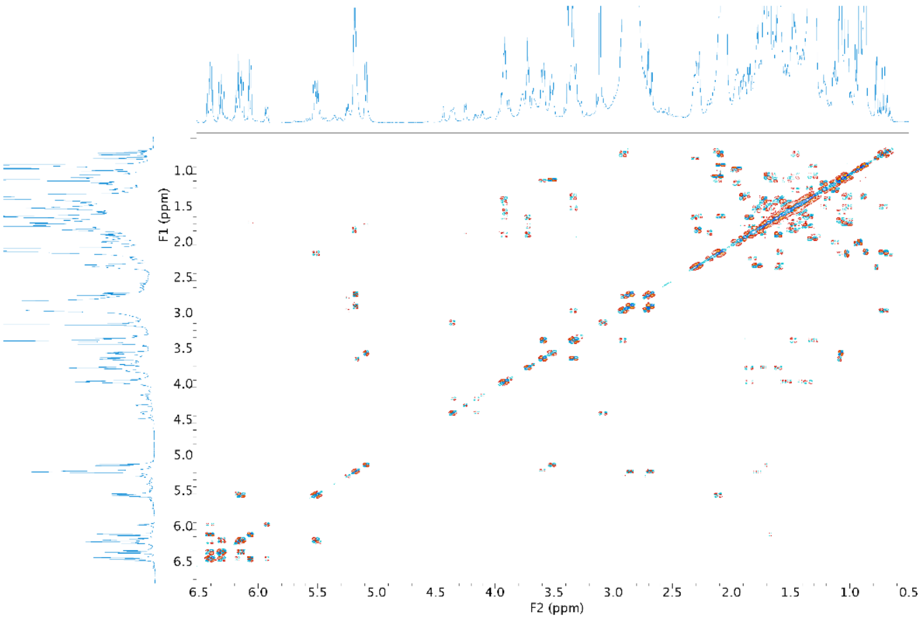

Supplementary Data 20. HSQC NMR spectrum (Acetone-d_6, 600 MHz) of compound **7**.

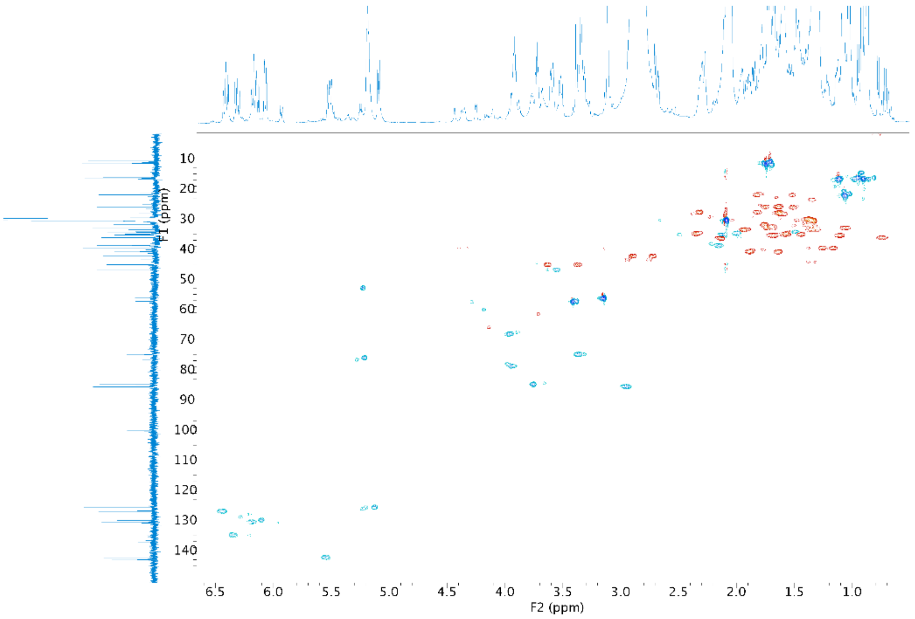

Supplementary Data 21. HMBC NMR spectrum (Acetone-d_6, 600 MHz) of compound **7**.

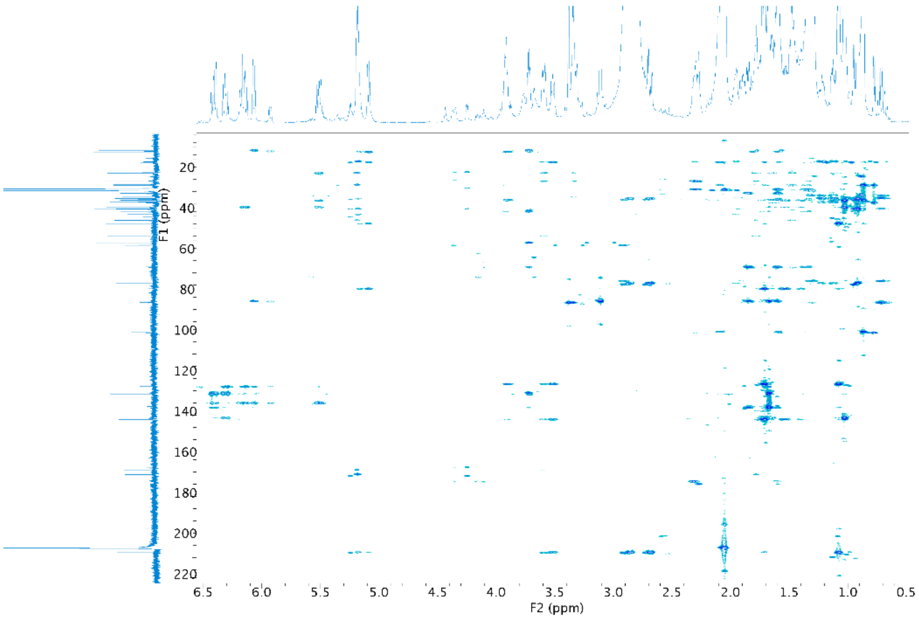

Supplementary Data 22. Chemical structure and 2D NMR key correlations for compound **9**.

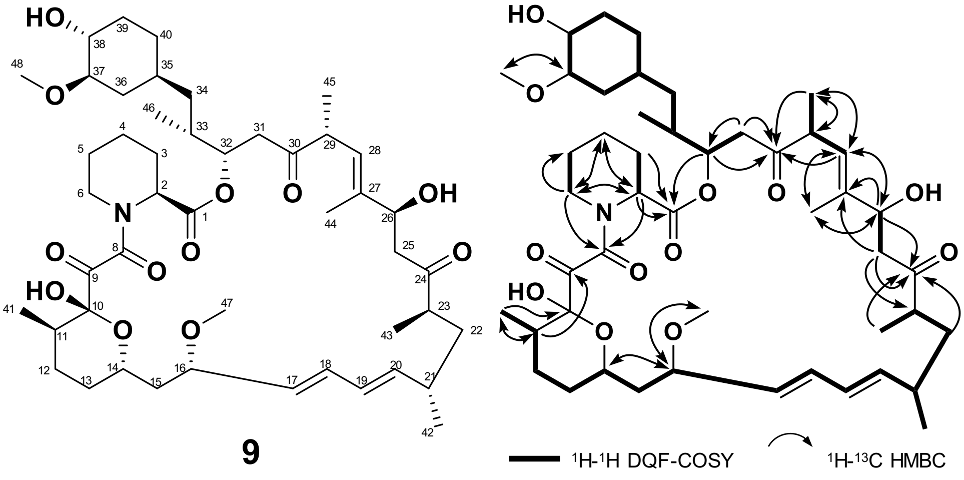

Supplementary Data 23. NMR data for compound **9** in Acetone-d_6 at 600 MHz for ^{1}H and 150 MHz for ^{13}C.

Position	δ_C	δ_H (multiplicity, J in Hz)	Position	δ_C	δ_H (multiplicity, J in Hz)
1	170.2	-	25	47.3	2.55 (m), 2.85 (m)
2	52.5	5.13 (m)	26	72.8	4.39 (m)
3	27.4	2.30 (m), 1.66 (m)	27	140.8	-
4	21.7	1.76 (m), 1.52 (m)	28	124.9	5.32 (d, 10.6)
5	25.7	1.61 (m), 1.43 (m)	29	46.4	3.43 (m)
6	45.0	3.52 (m), 3.13 (m)	30	208.3	-
7	-	-	31	42.1	2.84 (m), 2.68 (m)
8	167.8	-	32	75.1	5.25 (m)
9	197.3	-	33	34.7	1.92 (m)
10	99.8	-	34	39.6	1.27 (m), 1.16 (m)
11	35.5	2.15 (m)	35	34.0	1.44 (m)
12	27.9	1.63 (m)	36	35.8	2.09 (m), 0.68 (m)
13	31.6	1.84 (m), 1.34 (m)	37	85.4	2.90 (m)
14	67.9	4.19 (m)	38	74.7	3.29 (m)
15	42.5	1.84 (m), 1.49 (m)	39	33.2	1.88 (m), 1.29 (m)
16	79.7	3.70 (m)	40	32.5	0.99 (m)
17	132.5	5.43 (dd, 15.2, 8.6)	41	16.5	0.89 (d, 6.4)
18	134.5	6.26 (dd, 15.2, 10.8)	42	22.3	1.02 (d,6.0)
19	127.8	6.10 (dd, 14.6, 10.8)	43	15.4	0.96 (d, 6.6)
20	141.5	5.51 (dd, 14.6, 9.4)	44	13.7	1.74 (s)
21	36.9	2.27 (m)	45	16.3	1.03 (d, 6.7)
22	40.6	1.65 (m), 1.18 (m)	46	16.0	0.91 (d, 6.8)
23	45.3	2.51 (m)	47	55.9	3.17 (s)
24	212.9	-	48	57.1	3.36 (s)

Supplementary Data 24. ^{1}H NMR spectrum (Acetone-d_6, 600 MHz) of compound **9**.

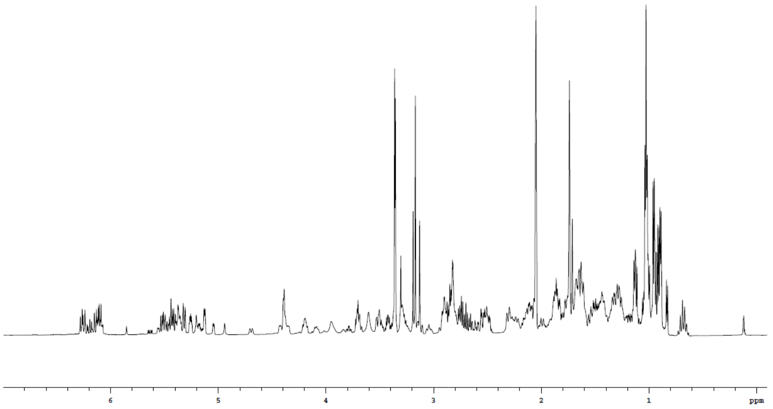

Supplementary Data 25. ^{13}C NMR spectrum (Acetone-d_6, 150 MHz) of compound **9**.

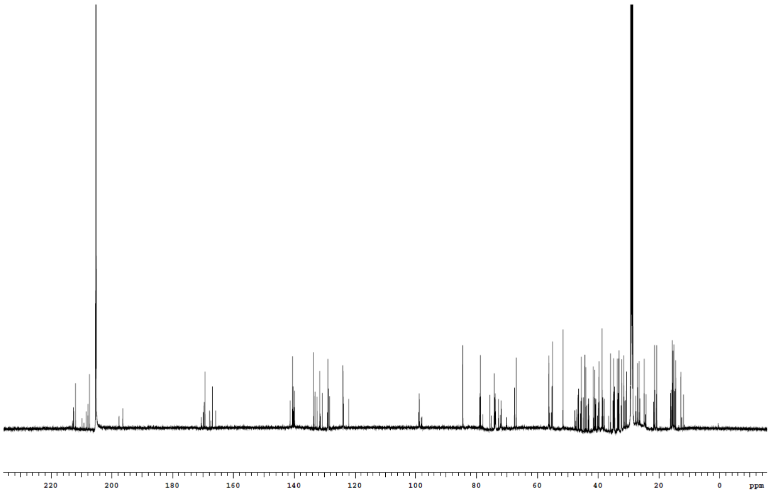

Supplementary Data 26. DQF-COSY NMR spectrum (Acetone-d_6, 600 MHz) of compound **9**.

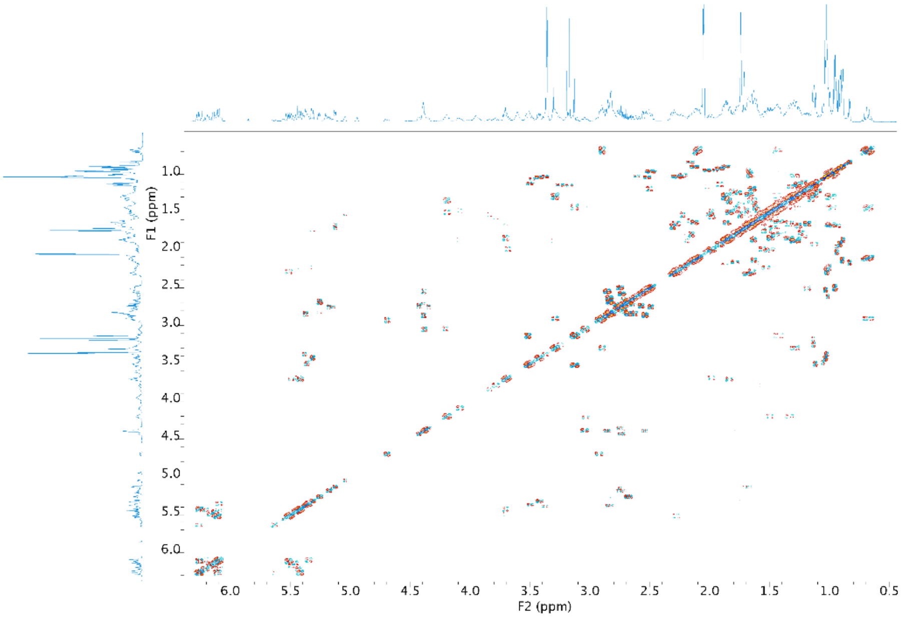

Supplementary Data 27. HSQC NMR spectrum (Acetone-d_6, 600 MHz) of compound **9**.

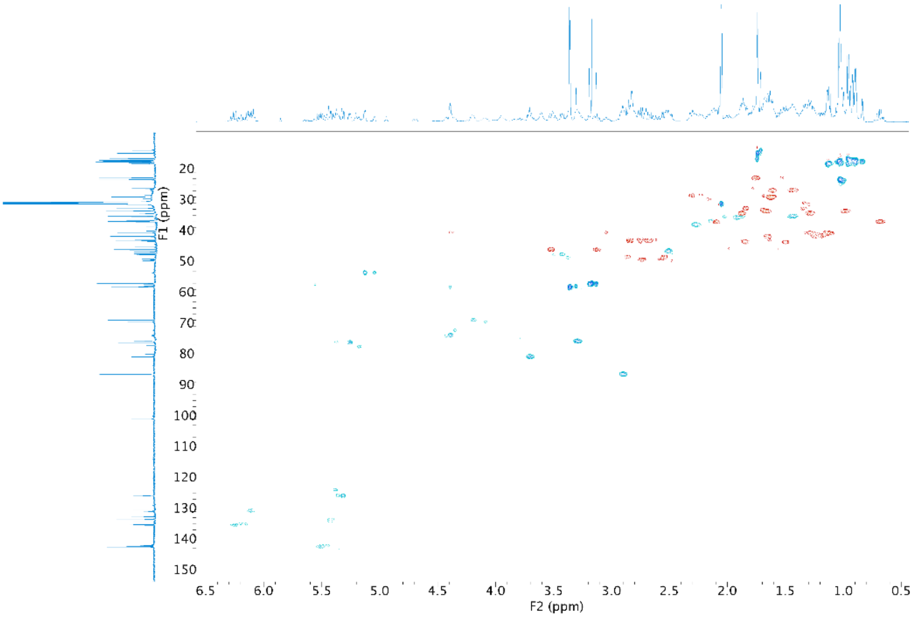

Supplementary Data 28. HMBC NMR spectrum (Acetone-d_6, 600 MHz) of compound **9**.

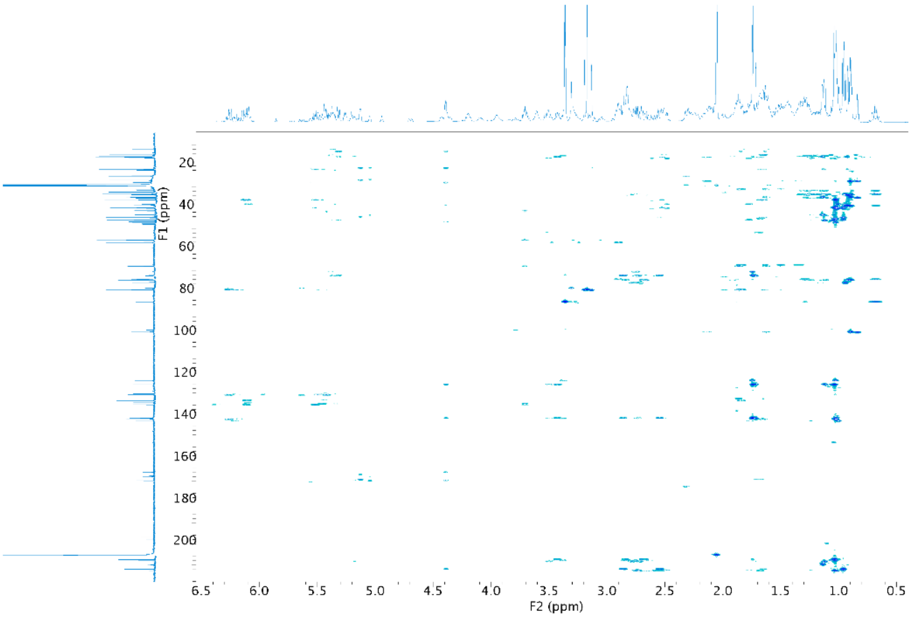

Supplementary Data 29. Chemical structure and 2D NMR key correlations for compound **10**

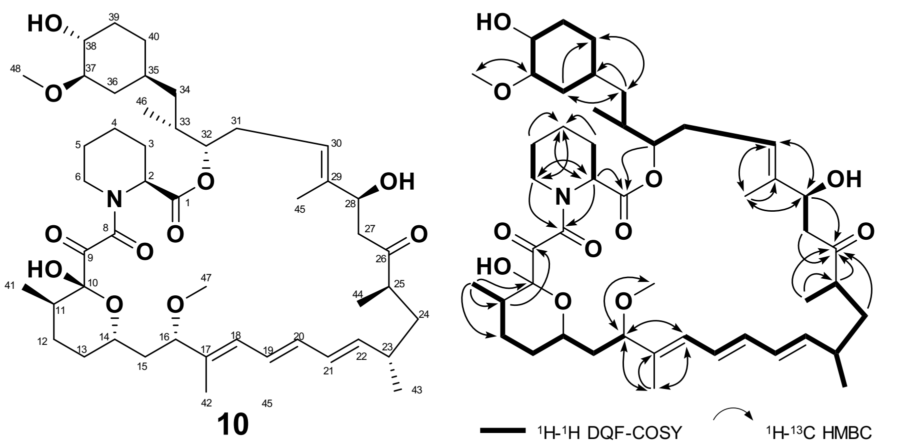

Supplementary Data 30. NMR data for compound **10** in acetone-d_6 at 500 MHz for ^{1}H and 125 MHz for ^{13}C.

Position	δ_C	δ_H (multiplicity, J in Hz)	Position	δ_C	δ_H (multiplicity, J in Hz)
1	170.2	-	25	46.6	2.50 (m)
2	52.0	5.13 (m)	26	213.1	-
3	27.2	2.25 (m), 1.66 (m)	27	49.8	2.56 (m), 2.31 (m)
4	21.8	1.75 (m), 1.41 (m)	28	71.9	4.40 (m)
5	25.7	1.61 (m), 1.40 (m)	29	141.8	-
6	45.0	3.50 (m), 3.22 (m)	30	118.3	5.45 (t, 7.0)
7	-	-	31	30.1	2.28 (m)
8	168.1	-	32	79.5	4.80 (m)
9	198.7	-	33	32.6	1.84 (m)
10	10.2	-	34	37.9	1.40 (m), 1.19 (m)
11	35.8	2.18 (m)	35	34.2	1.41 (m)
12	27.4	1.61 (m)	36	35.6	2.14 (m), 0.67 (m)
13	31.2	1.88 (m), 1.28 (m)	37	85.4	2.90 (m)
14	67.9	4.10 (m)	38	74.7	3.31 (m)
15	41.2	1.98 (m), 1.47 (m)	39	33.3	1.88 (m), 1.27 (m)
16	84.0	3.65 (m)	40	32.9	1.63 (m), 1.02 (m)
17	138.8	-	41	16.1	0.84 (d, 6.5)
18	128.6	6.04 (d, 11.0)	42	10.7	1.67 (s)
19	128.2	6.42 (dd, 14.0, 11.0)	43	21.9	0.98 (d, 6.0)
20	133.6	6.13 (dd, 14.0, 10.5)	44	18.1	0.97 (d, 6.0)
21	130.9	6.13 (dd, 14.0, 10.5)	45	13.6	1.56 (s)
22	141.2	5.24 (dd, 14.0, 9.5)	46	17.5	0.92 (d, 6.6)
23	39.8	2.16 (m)	47	56.0	3.09 (s)
24	41.0	1.81 (m), 1.31 (m)	48	57.1	3.37 (s)

Supplementary Data 31. ^{1}H NMR spectrum (Acetone-d_6, 500 MHz) of compound **10**.

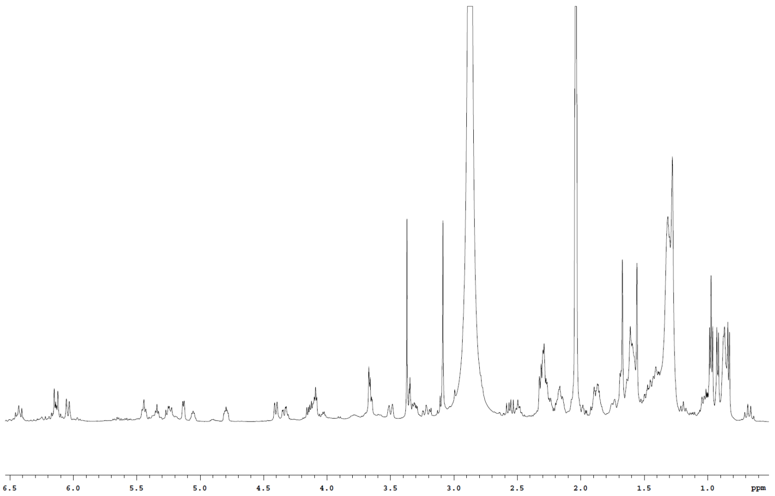

Supplementary Data X32. ^{13}C NMR spectrum (Acetone-d_6, 125 MHz) of compound **10**.

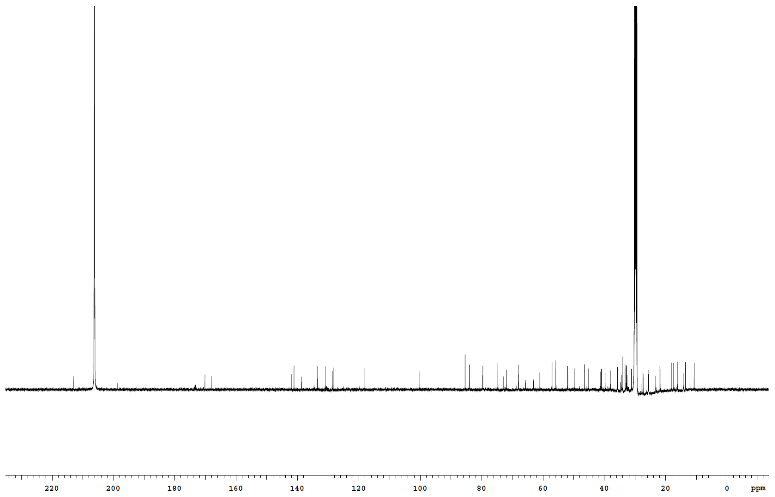

Supplementary Data 33. DQF-COSY NMR spectrum (Acetone-d_6, 500 MHz) of compound **10**.

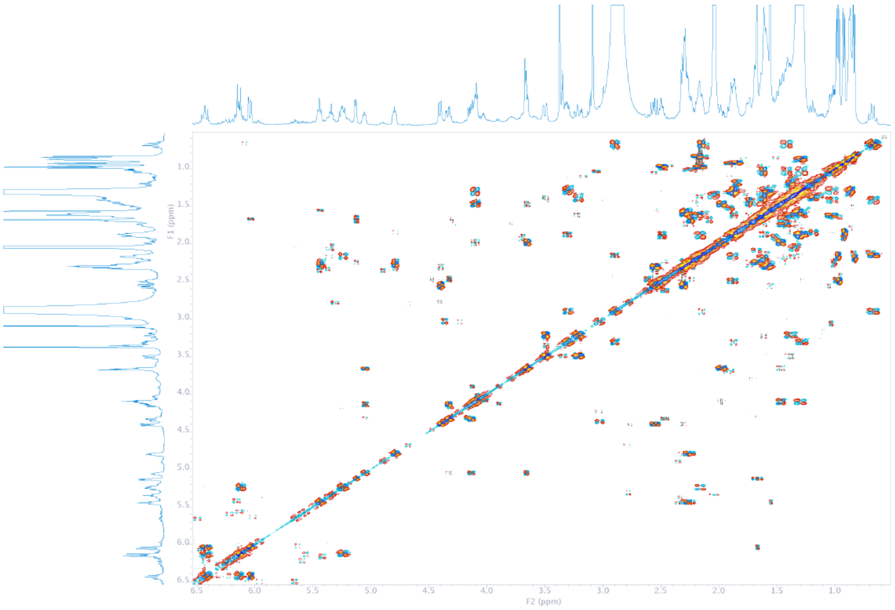

Supplementary Data 34. HSQC NMR spectrum (Acetone-d_6, 500 MHz) of compound **10**.

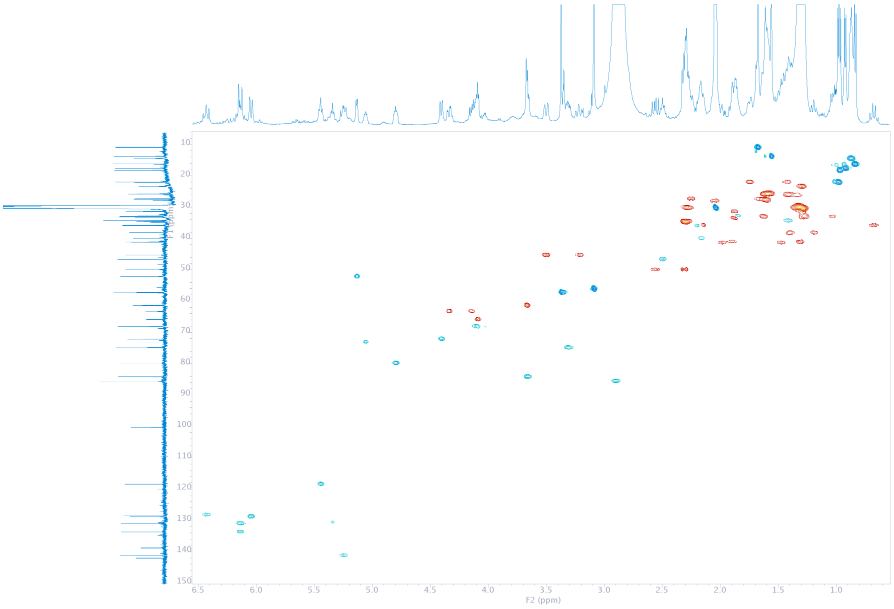

Supplementary Data 35. HMBC NMR spectrum (Acetone-d_6, 500 MHz) of compound **10**.

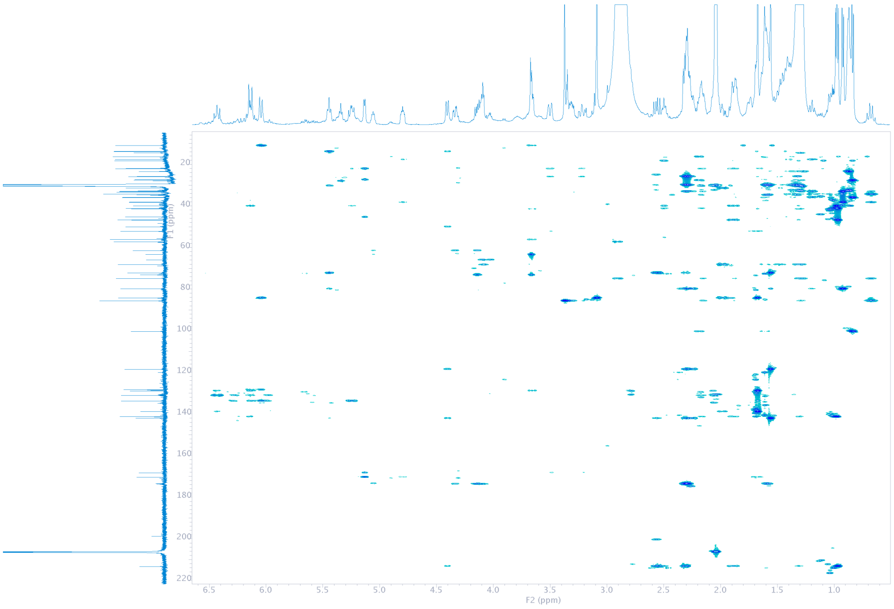

Supplementary Data 36. Chemical structure and 2D NMR key correlations for compound **11**

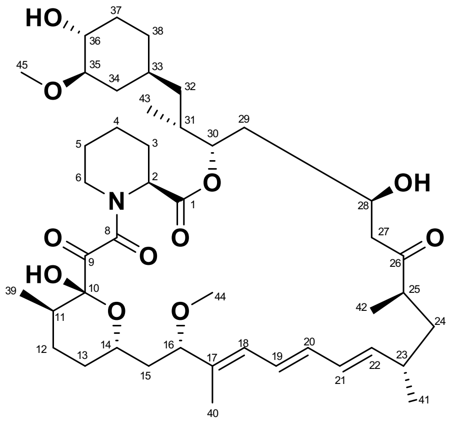

11

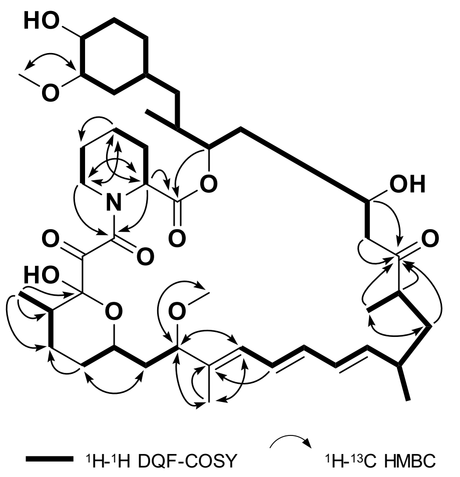

Supplementary Data 37. NMR data for compound **11** in acetone-d_6 at 600 MHz for ^{1}H and 150 MHz for ^{13}C.

Position	δ_C	δ_H (multiplicity, J in Hz)	Position	δ_C	δ_H (multiplicity, J in Hz)
1	170.7	-	24	41.1	1.89 (m), 1.43 (m)
2	53.0	5.17 (m)	25	46.4	2.50 (m)
3	27.7	2.32 (m), 1.66 (m)	26	213.4	-
4	21.7	1.68 (m), 1.46 (m)	27	50.2	2.70 (m), 2.43 (m)
5	25.9	1.66 (m), 1.41 (m)	28	67.1	4.05 (m)
6	44.9	3.54 (m), 3.34 (m)	29	38.1	1.73 (m), 1.53 (m)
7	-	-	30	79.4	5.18 (m)
8	167.4	-	31	34.5	1.92 (m)
9	196.3	-	32	39.9	1.36 (m), 1.12 (m)
10	99.7	-	33	34.5	1.55 (m)
11	35.5	2.15 (m)	34	35.5	2.20 (m), 0.76(m)
12	27.4	1.62 (m)	35	85.4	2.93 (m)
13	30.9	1.76 (m), 1.35 (m)	36	74.8	3.31 (m)
14	67.8	3.99 (m)	37	33.2	1.87 (m), 1.30 (m)
15	41.1	1.88 (m), 1.43 (m)	38	32.5	1.72 (m), 1.02 (m)
16	84.0	3.75 (m)	39	16.3	0.85 (d, 6.0)
17	139.0	-	40	10.5	1.69 (s)
18	128.5	6.06 (d, 11.0)	41	21.6	0.98 (d, 6.6)
19	127.9	6.45 (dd, 14.0, 11.0)	42	18.5	0.99 (d, 6.0)
20	133.7	6.15 (dd, 14.0, 10.2)	43	16.7	0.98 (d, 6.6)
21	130.7	6.13 (dd, 14.0, 10.2)	44	55.4	3.12 (s)
22	140.9	5.27 (dd, 14.0, 8.4)	45	56.3	3.36 (s)
23	39.0	2.19 (m)			

Supplementary Data 38. ^{1}H NMR spectrum (Acetone-d_6, 600 MHz) of compound **11**.

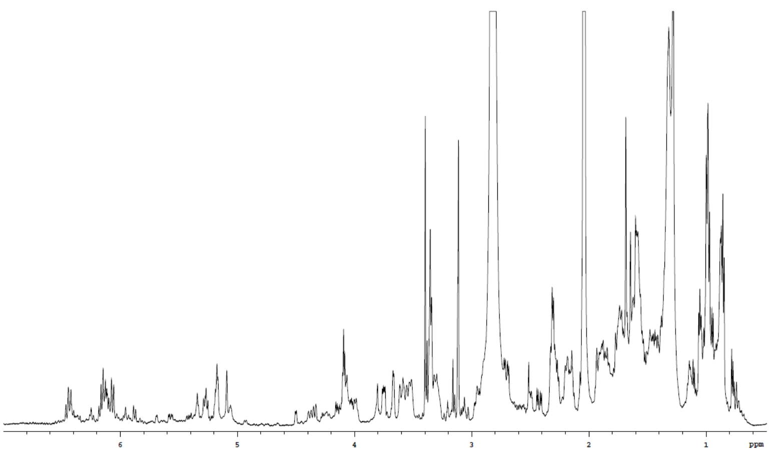

Supplementary Data 39. ^{13}C NMR spectrum (Acetone-d_6, 150 MHz) of compound **11**.

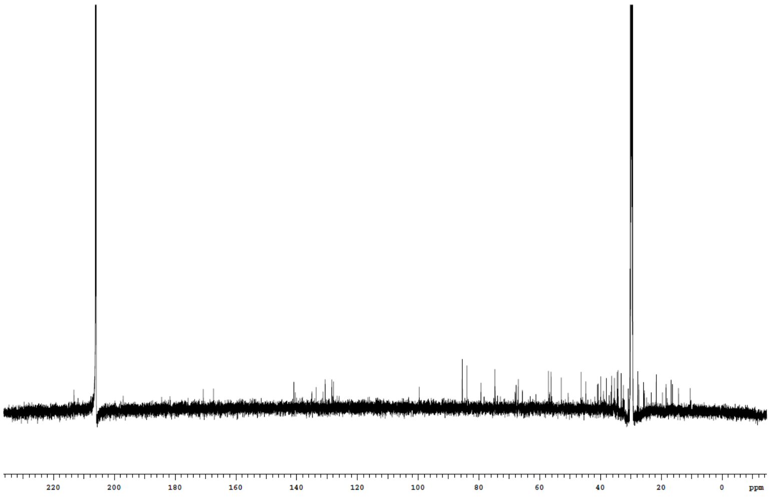

Supplementary Data 40. DQF-COSY NMR spectrum (Acetone-d_6, 600 MHz) of compound 11.

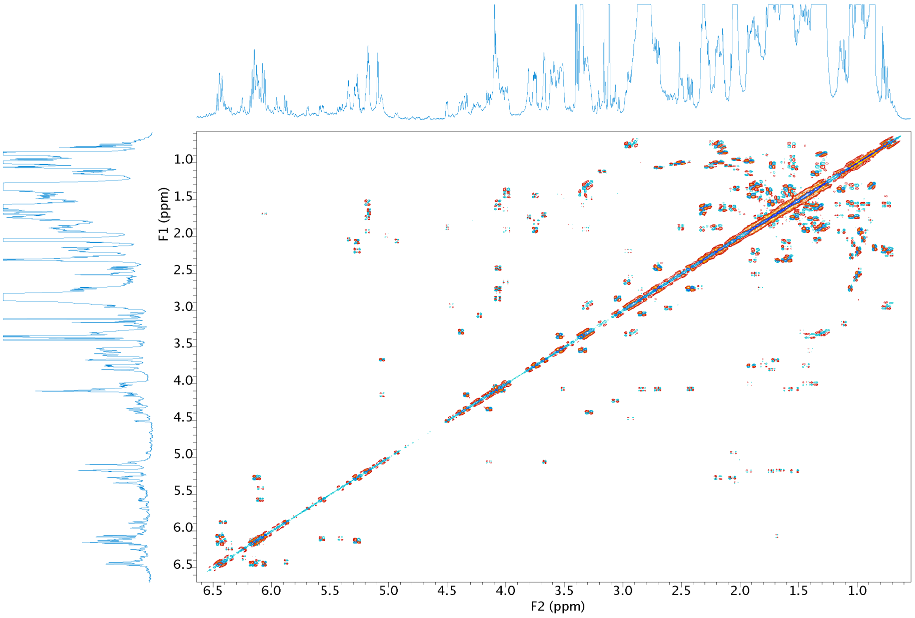

Supplementary Data 41. HSQC NMR spectrum (Acetone-d_6, 600 MHz) of compound **11**.

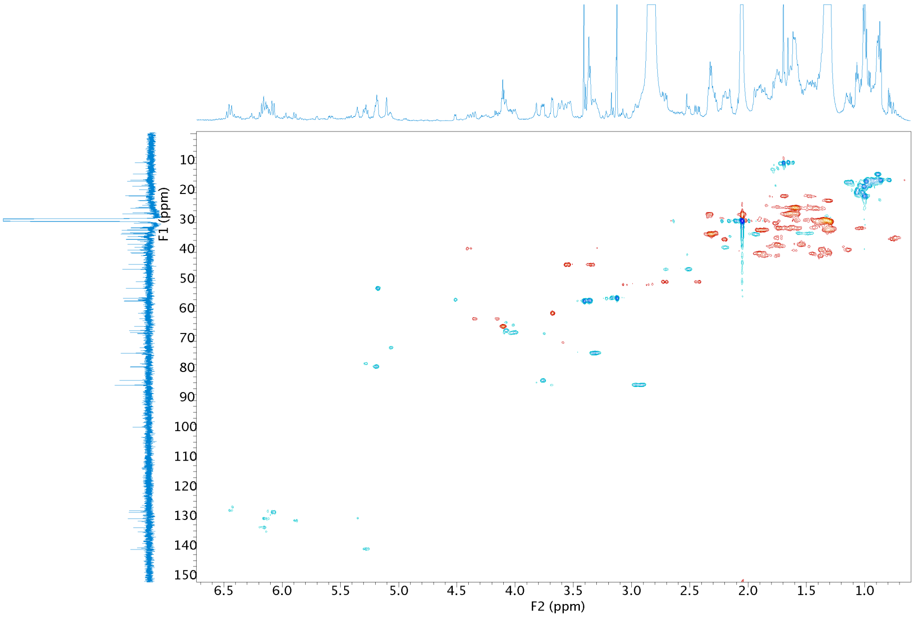

Supplementary Data 42. HMBC NMR spectrum (Acetone-d_6, 600 MHz) of compound **11**.

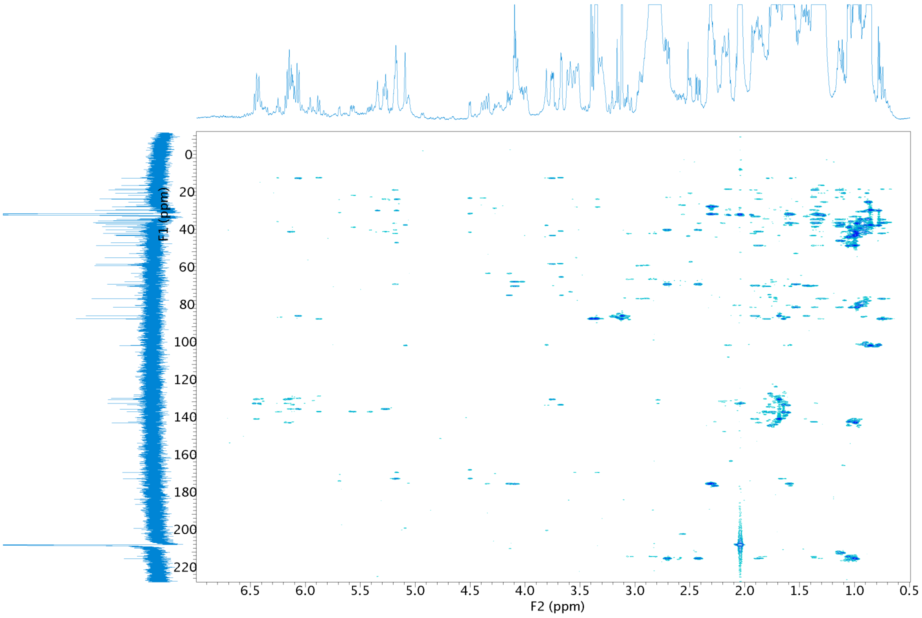

Supplementary Data 43. Chemical structure and 2D NMR key correlations for compound **12**

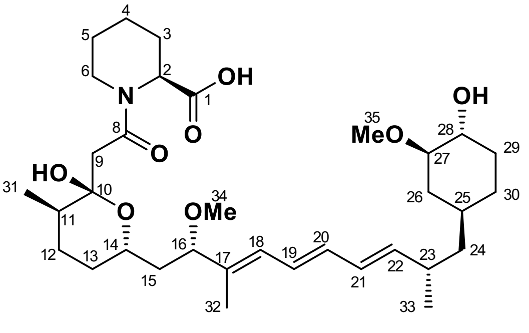

12

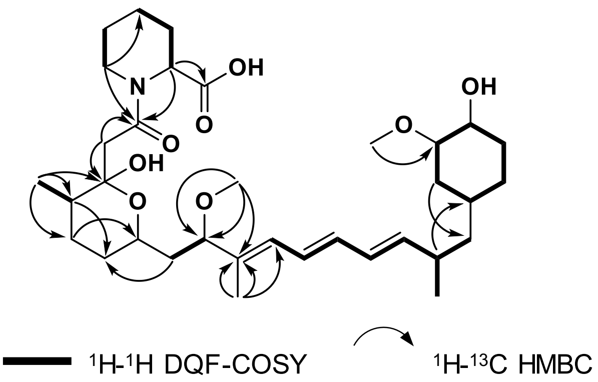

Supplementary Data 44. NMR data for compound **12** in chloroform-*d* at 600 MHz for ^{1}H and 150 MHz for ^{13}C.

Position	δ_C	δ_H (multiplicity, *J* in Hz)	Position	δ_C	δ_H (multiplicity, *J* in Hz)
1	166.9		19	133.3	6.34 (m)
2	66.2	3.69 (d, 11.2)	20	126.3	6.35 (m)
3	20.8	1.75 (m), 1.49 (m)	21	129.3	6.07 (dd, 15.2, 9.3)
4	32.2	1.48 (m)	22	140.6	5.58 (dd, 15.2, 8.3)
5	25.2	1.75 (m), 1.58 (m)	23	34.4	2.28 (m)
6	44.1	3.93 (d, 12.7), 3.27 (dt, 12.7, 2.4)	24	44	1.21 (m)
7	-	-	25	33.4	1.37 (m)
8	173.3	-	26	35.4	2.06 (d, 10.9), 0.81 (m)
9	39.4	2.81 (dd, 14.4, 7.4), 2.56 (dd, 14.4, 9.1)	27	84.5	2.96 (m)
10	98.3	-	28	74.1	3.39 (m)
11	39.4	1.48 (m)	29	31.3	1.98 (m), 1.30 (m)
12	27.5	1.61 (m), 1.48 (m)	30	30.2	1.76 (m), 0.86 (m)
13	26.5	2.36 (d, 13.9), 1.73 (m)	31	16.9	0.93 (d, 7.6)
14	84.4	3.73 (m)	32	10.4	1.62 (s)
15	39.2	1.67 (m)	33	21.3	0.98 (d, 6.73)
16	84.3	3.73 (dd, 10.4, 4.8)	34	55.7	3.13 (s)
17	135.2	-	35	56.4	3.39 (s)
18	129.5	6.04 (d, 10.2)			

Supplementary Data 45. ^{1}H NMR spectrum (chloroform-*d*, 600 MHz) of compound **12**.

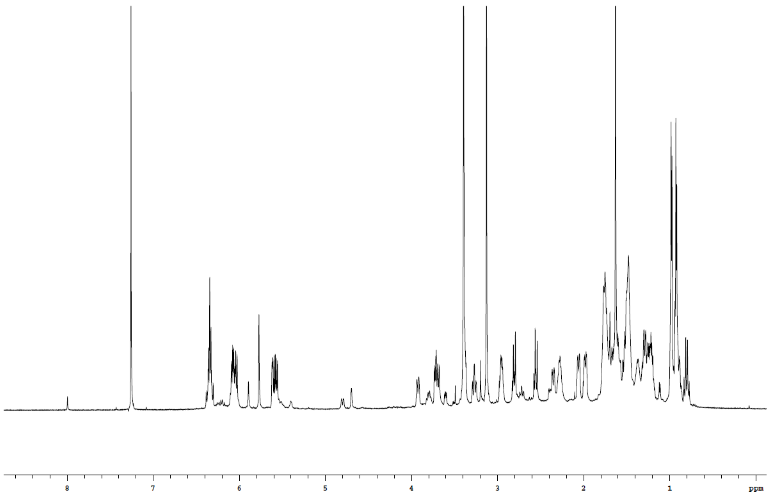

Supplementary Data 46. ^{13}C NMR spectrum (chloroform-*d*, 150 MHz) of compound **12**.

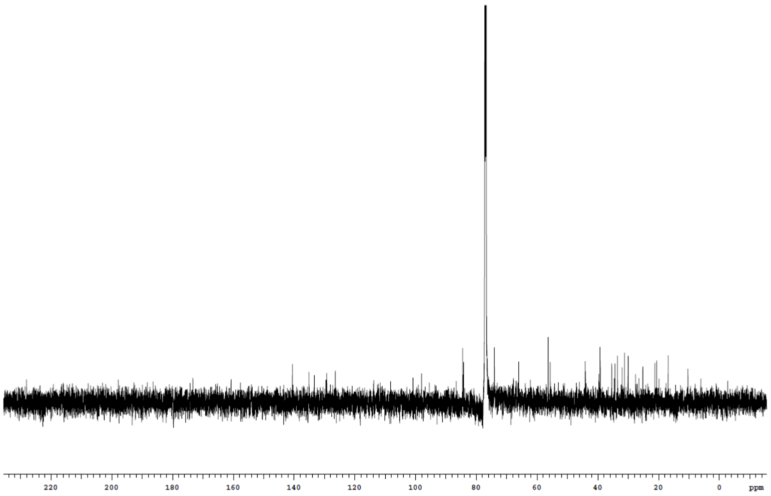

Supplementary Data 47. DQF-COSY NMR spectrum (chloroform-*d*, 600 MHz) of compound **12**.

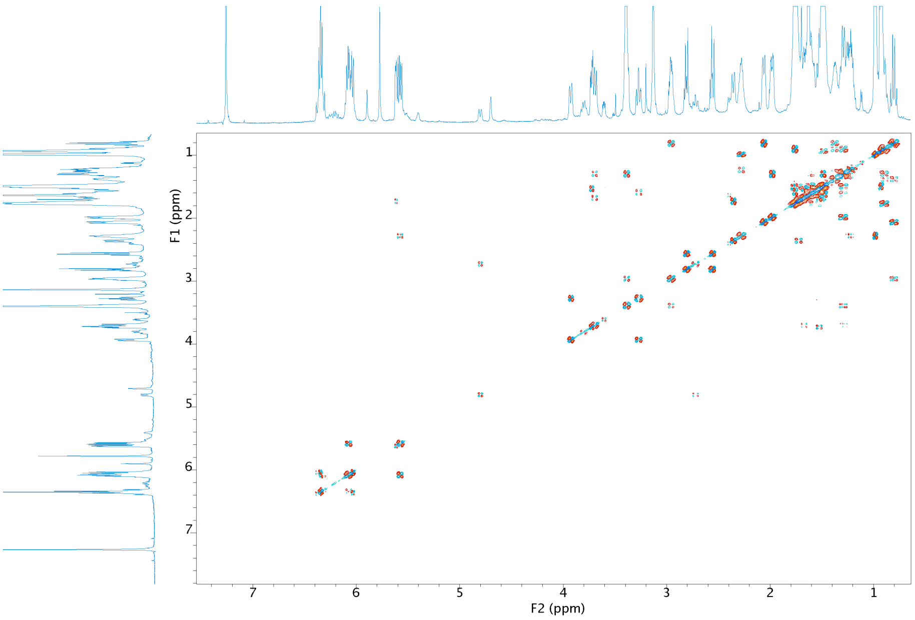

Supplementary Data 48. HSQC NMR spectrum (chloroform-*d*, 600 MHz) of compound **12**.

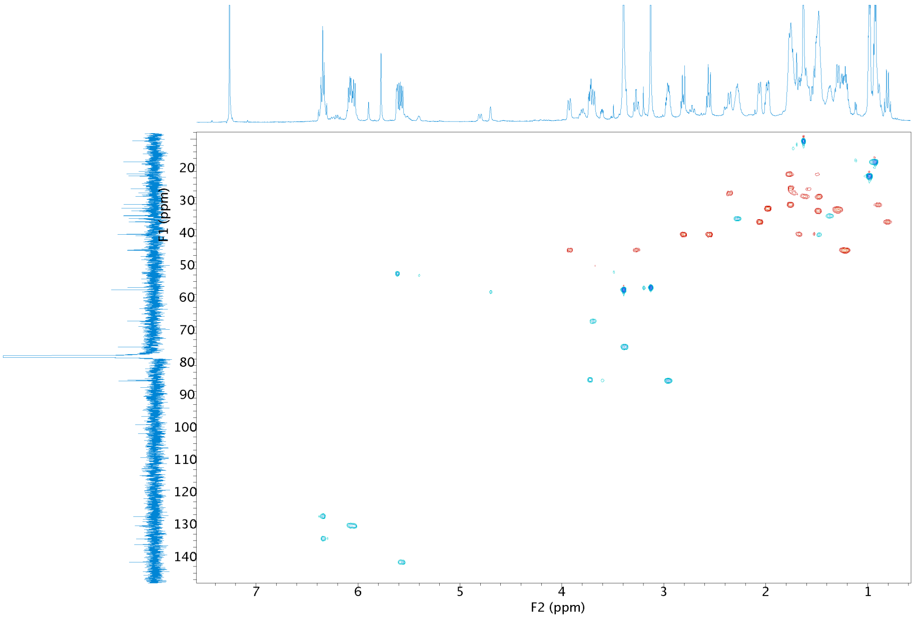

Supplementary Data 49. HMBC NMR spectrum (chloroform-*d*, 600 MHz) of compound **12**.

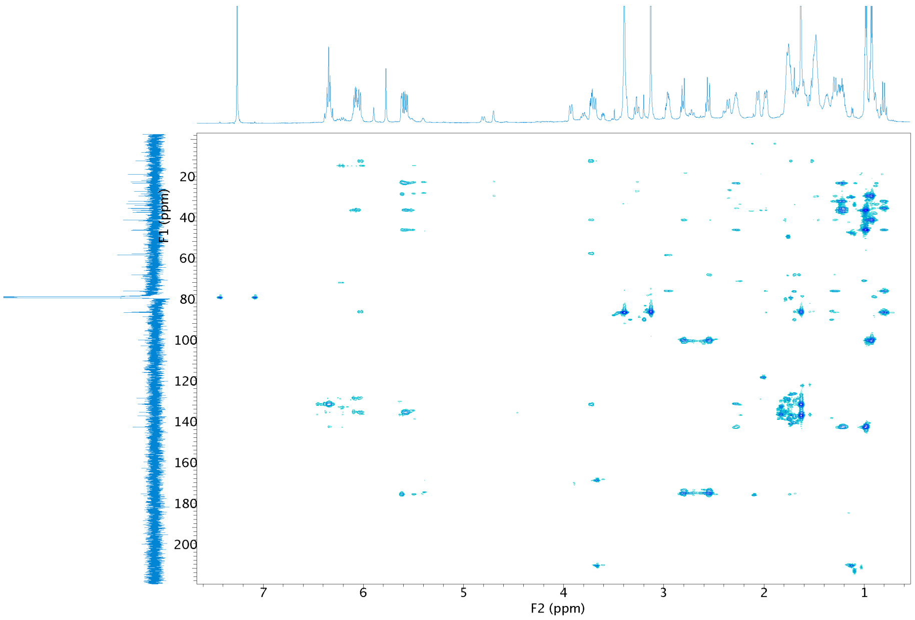

Supplementary Data 50. Chemical structure and 2D NMR key correlations for compound **13**

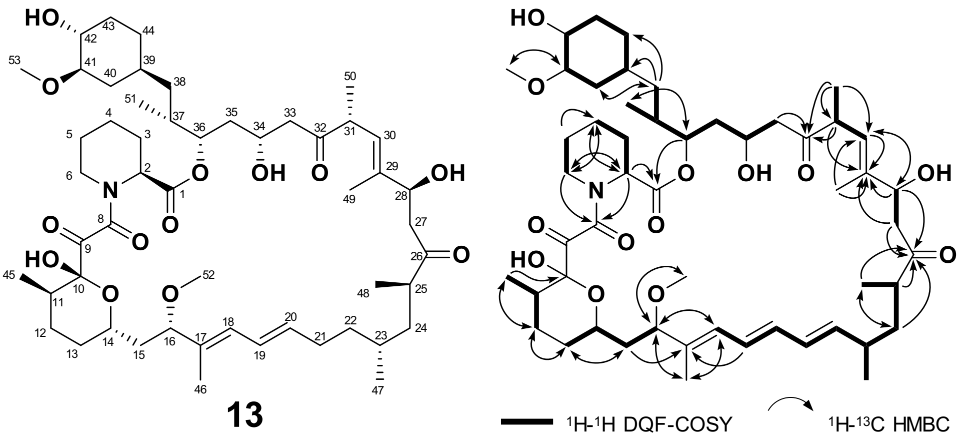

Supplementary Data 51. NMR data for compound **13** in acetone-d_6 at 500 MHz for ^{1}H and 125 MHz for ^{13}C.

Position	δ_C	δ_H (multiplicity, J in Hz)	Position	δ_C	δ_H (multiplicity, J in Hz)
1	170.5	-	28	74.3	4.35 (m)
2	52.3	5.26 (m)	29	140.0	-
3	27.5	2.34 (m), 1.79 (m)	30	125.6	5.32 (d, 10.6)
4	21.5	1.80 (m), 1.44 (m)	31	46.5	3.46 (m)
5	25.8	1.74 (m), 1.45 (m)	32	210.8	-
6	45.0	3.56 (m), 3.48 (m)	33	47.9	2.65 (m), 2.52 (m)
7	-	-	34	66.0	4.14 (m)
8	168.2	-	35	38.5	1.76 (m)
9	198.9	-	36	77.1	5.14 (m)
10	99.4	-	37	34.6	1.90 (m)
11	34.7	2.03 (m)	38	39.1	1.34 (m), 1.13 (m)
12	28.0	1.58 (m)	39	34.1	1.44 (m)
13	31.7	1.70 (m), 1.36 (m)	40	35.8	2.12 (m), 0.68 (m)
14	68.0	3.92 (m)	41	85.4	2.89 (m)
15	39.9	1.83 (m), 1.58 (m)	42	74.8	3.28 (m)
16	84.9	3.71 (m)	43	33.2	1.86 (m), 1.26 (m)
17	136.8	-	44	32.6	1.63 (m), 0.97 (m)
18	130.0	6.05 (d, 11.0)	45	16.2	0.92 (d, 6.0)
19	127.4	6.42 (dd, 14.0, 11.0)	46	10.5	1.66 (s)
20	134.3	6.31 (dd, 14.0, 10.5)	47	22.2	0.98 (d, 6.0)
21	130.9	6.18 (dd, 15.0, 10.5)	48	15.8	1.01 (d, 6.6)
22	140.9	5.38 (dd, 15.0, 9.0)	49	12.1	1.68 (s)
23	36.3	2.23 (m)	50	16.8	1.02 (d, 6.0)
24	39.8	1.56 (m), 1.24 (m)	51	16.2	0.92 (d, 6.6)
25	45.3	2.47 (m)	52	55.9	3.10 (s)
26	213.2	-	53	57.0	3.36 (s)
27	47.9	2.67 (m), 2.60 (m)			

Supplementary Data 52. ^{1}H NMR spectrum (Acetone-d_6, 500 MHz) of compound **13**.

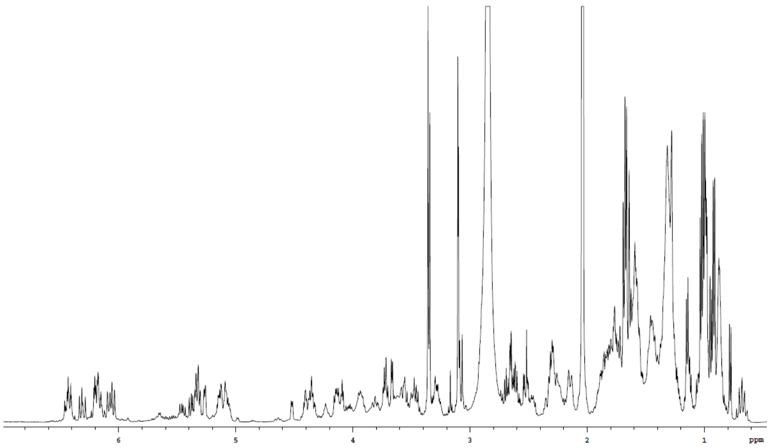

Supplementary Data 53. ^{13}C NMR spectrum (Acetone-d_6, 125 MHz) of compound **13**.

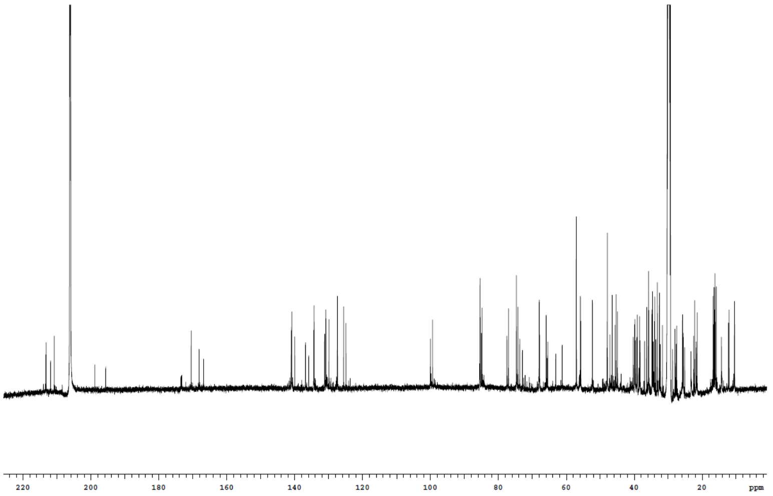

Supplementary Data 54. DQF-COSY NMR spectrum (Acetone-d_6, 500 MHz) of compound **13**.

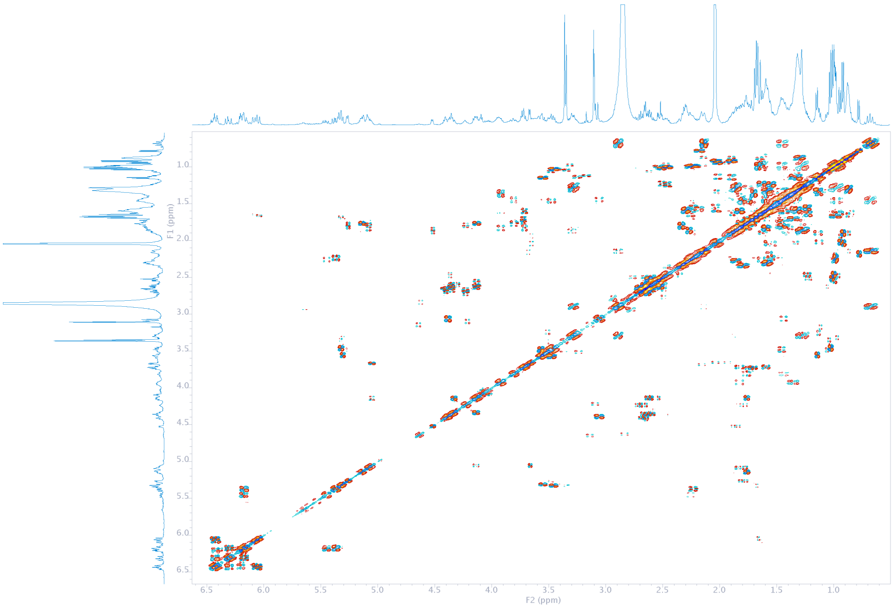

Supplementary Data 55. HSQC NMR spectrum (Acetone-d_6, 500 MHz) of compound **13**.

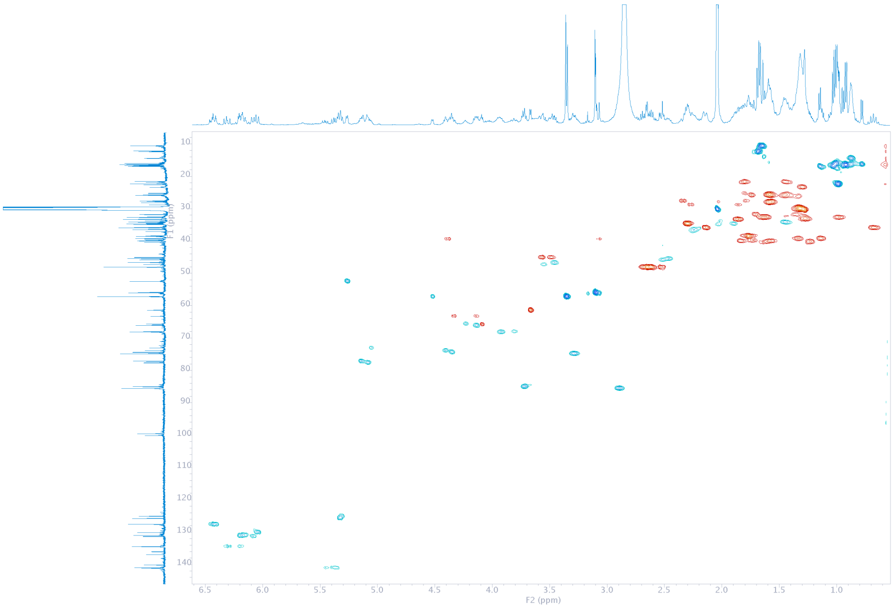

Supplementary Data 56. HMBC NMR spectrum (Acetone-d_6, 500 MHz) of compound **13**.

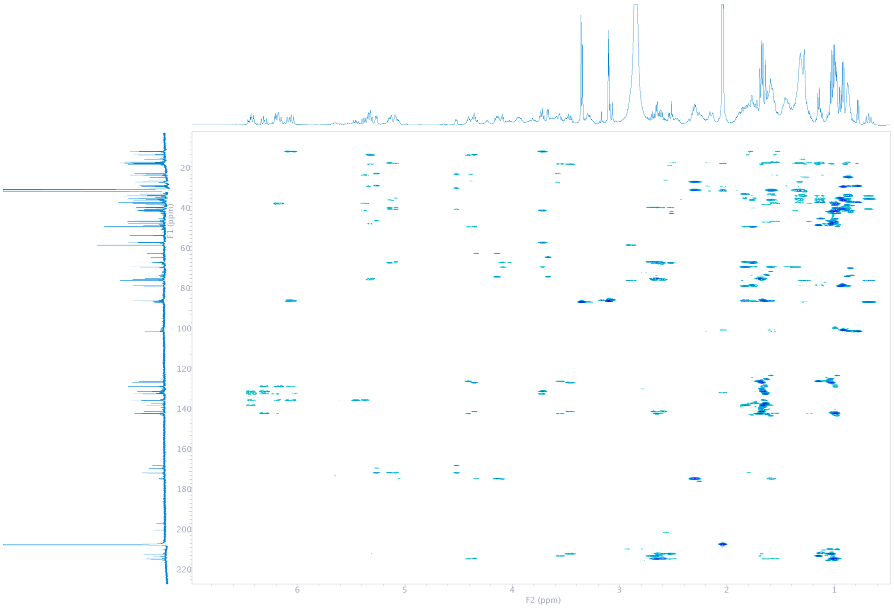

Supplementary Data 57. Chemical structure and 2D NMR key correlations for compound **14**.

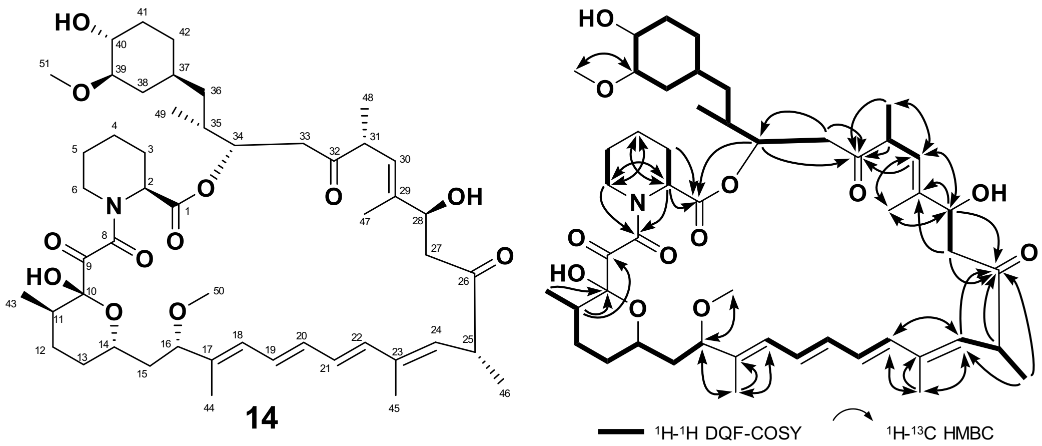

Supplementary Data 58. NMR data for compound **14** in Acetone-d_6 at 600 MHz for ^{1}H and 150 MHz for ^{13}C.

Position	δ_C	δ_H (multiplicity, J in Hz)	Position	δ_C	δ_H (multiplicity, J in Hz)
1	170.2	-	27	47.4	2.66 (m), 2.96 (m)
2	52.7	5.30 (m)	28	73.2	4.54 (m)
3	27.4	1.86 (m), 2.32 (m)	29	141.0	-
4	21.2	1.60 (m), 1.82 (m)	30	126.0	5.15 (m)
5	25.7	1.61 (m), 1.81 (m)	31	47.1	3.47 (m)
6	44.7	3.46 (m), 3.71 (m)	32	208.1	-
7	-	-	33	43.1	2.62 (m), 2.75 (m)
8	167.2	-	34	76.4	5.22 (m)
9	195.3	-	35	34.8	1.91 (m)
10	99.8	-	36	39.1	1.22 (m), 1.37 (m)
11	34.7	2.10 (m)	37	34.1	1.48 (m)
12	27.8	1.62 (m)	38	35.5	0.74 (m), 2.15 (m)
13	31.9	1.41 (m), 1.69 (m)	39	85.5	2.94 (m)
14	67.7	3.87 (m)	40	74.7	3.31 (m)
15	39.7	1.71 (m), 1.83 (m)	41	33.2	1.87 (m), 1.30 (m)
16	85.0	3.78 (m)	42	32.7	1.02 (m)
17	137.1	-	43	16.4	0.88 (d, 6.5)
18	130.8	6.25 (d, 10.8)	44	10.4	1.72 (s)
19	128.6	6.61 (dd, 14.0, 10.8)	45	12.9	2.02 (s)
20	135.5	6.53 (dd, 14.0, 10.5)	46	15.6	1.12 (d, 6.5)
21	129.3	6.49 (dd, 14.4, 10.5)	47	11.6	1.70 (s)
22	138.3	6.34 (d, 14.4)	48	15.9	1.04 (d, 6.5)
23	137.6	-	49	16.1	0.94 (d, 6.5)
24	132.6	5.26 (d, 10.3)	50	57.2	3.37 (s)
25	47.7	3.72 (m)	51	56.0	3.15 (s)
26	208.6	-			

Supplementary Data 59. ^{1}H NMR spectrum (Acetone-d_6, 600 MHz) of compound **14**.

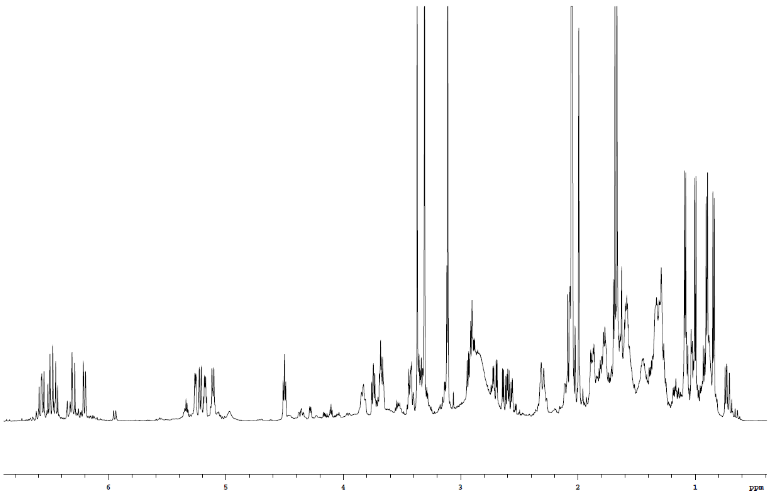

Supplementary Data 60. ^{13}C NMR spectrum (Acetone-d_6, 150 MHz) of compound **14**.

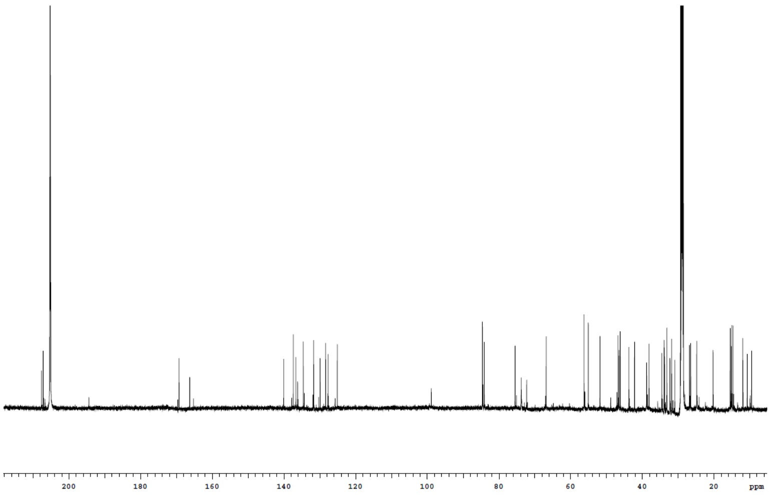

Supplementary Data 61. DQF-COSY NMR spectrum (Acetone-d_6, 600 MHz) of compound **14**.

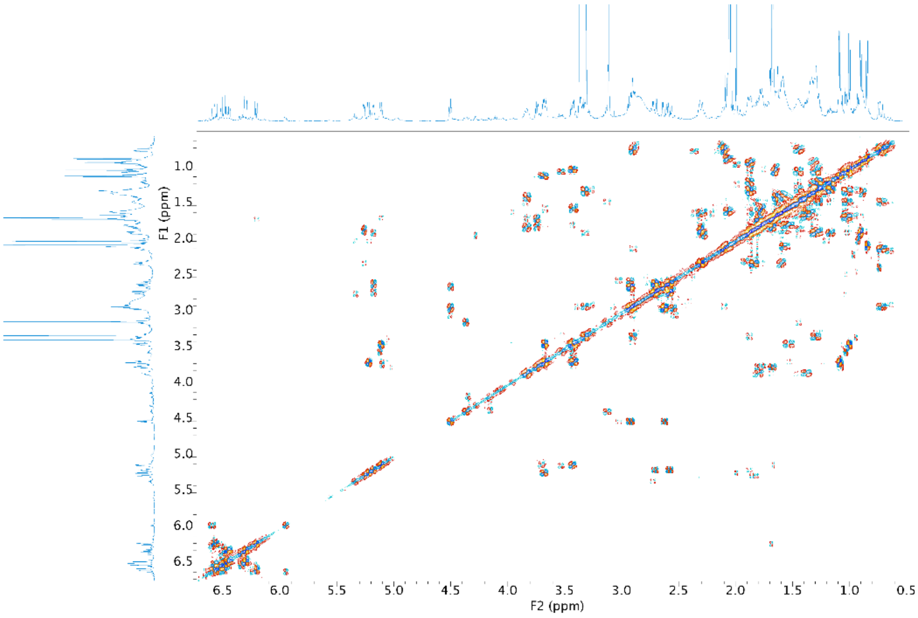

Supplementary Data 62. HSQC NMR spectrum (Acetone-d_6, 600 MHz) of compound **14**.

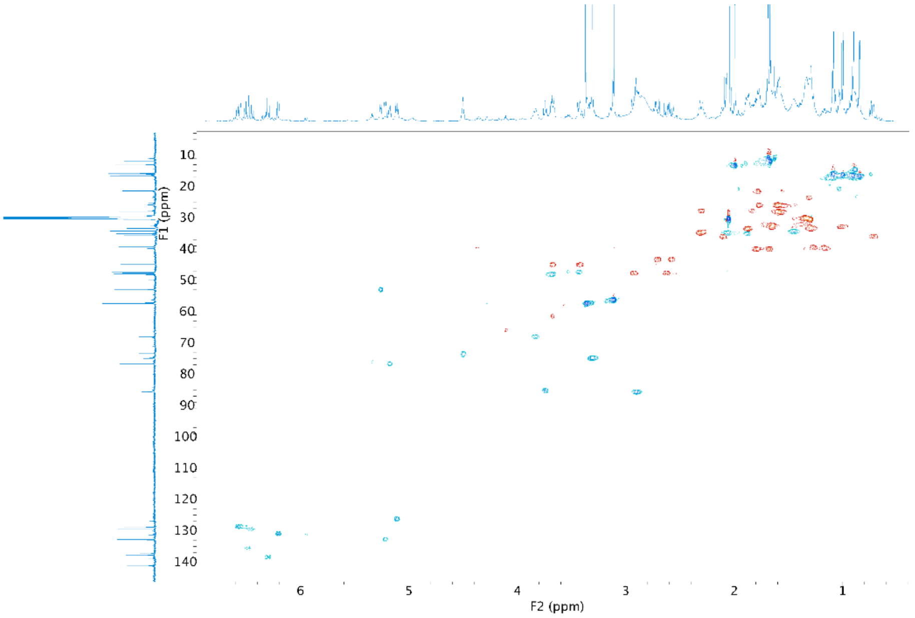

Supplementary Data 63. HMBC NMR spectrum (Acetone-d_6, 600 MHz) of compound **14**.

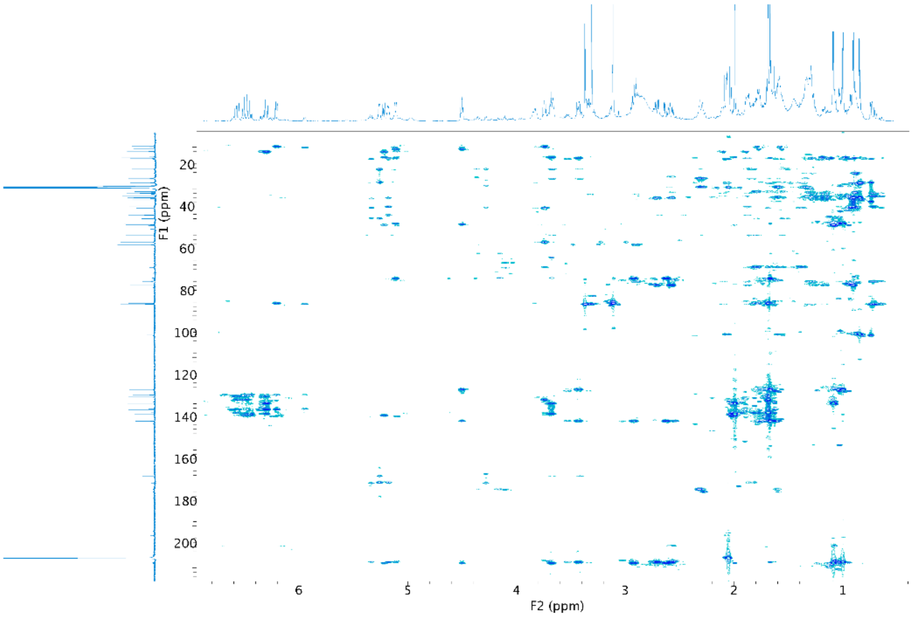

Supplementary Data 64. Chemical structure and 2D NMR key correlations for compound **15**

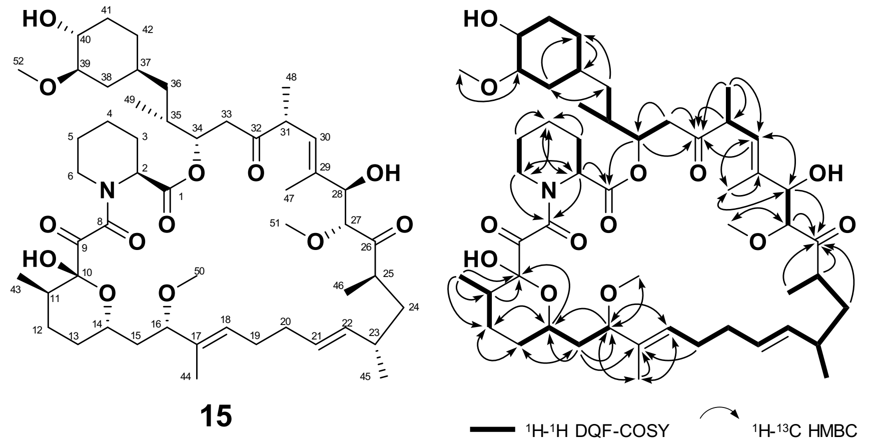

Supplementary Data 65. NMR data for compound **15** in acetone-d_6 at 600 MHz for ^{1}H and 150 MHz for ^{13}C.

Position	δ_C	δ_H (multiplicity, J in Hz)	Position	δ_C	δ_H (multiplicity, J in Hz)
1	169.4	-	27	86.0	4.02 (d, 5.2)
2	51.5	5.19 (m)	28	77.1	4.23 (d, 5.2)
3	27.2	2.32 (m), 1.79 (m)	29	137.7	-
4	20.9	1.78 (m), 1.45 (m)	30	126.0	5.38 (d, 10.2)
5	25.2	1.71 (m), 1.44 (m)	31	46.9	3.46 (m)
6	44.3	3.52 (m), 3.39 (m)	32	208.2	-
7	-	-	33	40.9	2.79 (m), 2.63 (m)
8	167.6	-	34	75.9	5.23 (m)
9	195.0	-	35	34.1	1.94 (m)
10	98.9	-	36	39.2	1.27 (m), 1.13 (m)
11	34.3	2.01 (m)	37	33.5	1.43 (m)
12	27.8	1.58 (m)	38	35.6	2.08 (m), 0.68 (m)
13	31.3	1.32 (m)	39	84.4	2.88 (m)
14	67.5	3.92 (m)	40	74.2	3.28 (m)
15	39.6	1.75 (m), 1.57 (m)	41	32.6	1.86 (m), 1.26 (m)
16	84.2	3.65 (m)	42	31.8	1.67 (m), 0.97 (m)
17	134.0	-	43	16.1	0.91 (d, 6.0)
18	129.4	5.41 (m)	44	9.8	1.52 (s)
19	28.5	2.13 (m)	45	22.1	0.98 (d, 6.6)
20	33.2	2.13 (m)	46	13.9	0.95 (d, 6.6)
21	130.3	5.57 (ddd,15.0, 6.0, 5.7)	47	13.2	1.84 (s)
22	135.8	5.30 (dd, 15.0, 9.2)	48	15.7	1.05 (d, 6.6)
23	35.2	2.20 (m)	49	15.3	0.92 (d, 6.6)
24	40.0	1.51 (m), 1.09 (m)	50	55.1	3.10 (s)
25	41.4	2.72 (m)	51	57.7	3.28 (s)
26	211.8	-	52	56.6	3.35 (s)

Supplementary Data 66. ^{1}H NMR spectrum (Acetone-d_6, 600 MHz) of compound **15**.

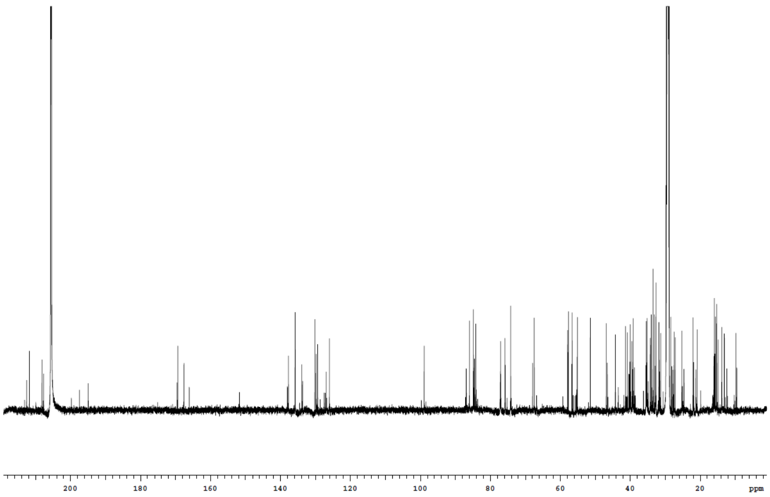

Supplementary Data 67. ^{13}C NMR spectrum (Acetone-d_6, 150 MHz) of compound **15**.

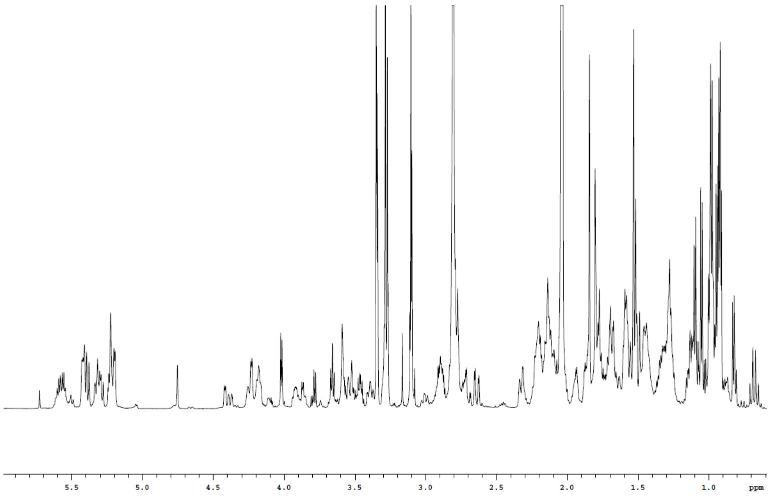

Supplementary Data 68. DQF-COSY NMR spectrum (Acetone-d_6, 600 MHz) of compound **15**.

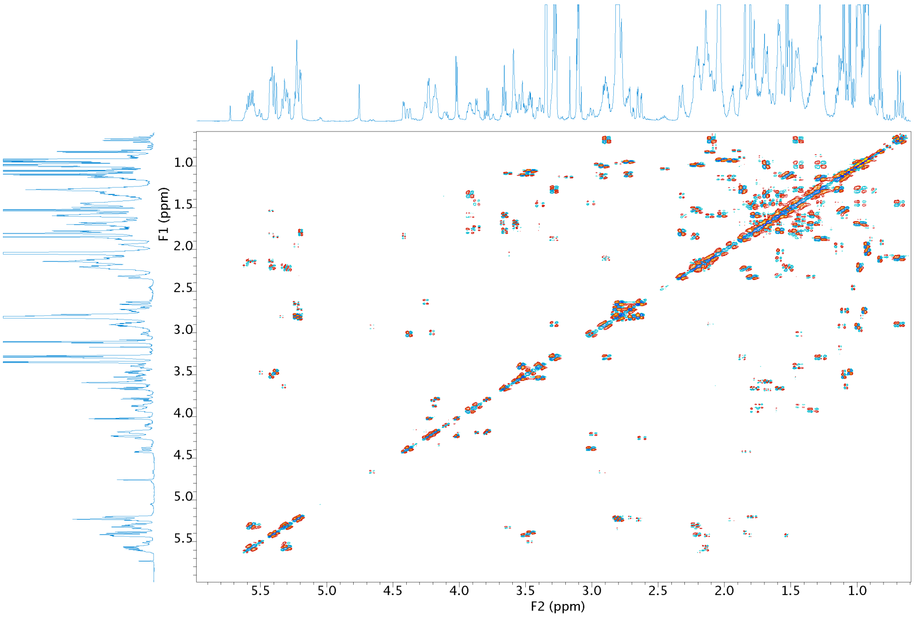

Supplementary Data 69. HSQC NMR spectrum (Acetone-d_6, 600 MHz) of compound **15**.

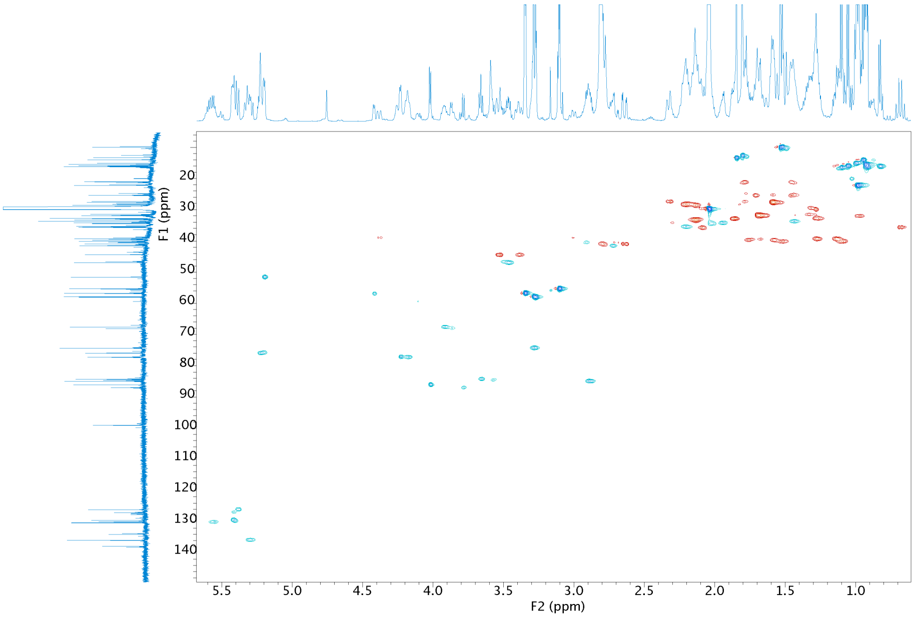

Supplementary Data 70. HMBC NMR spectrum (Acetone-d_6, 600 MHz) of compound **15**.

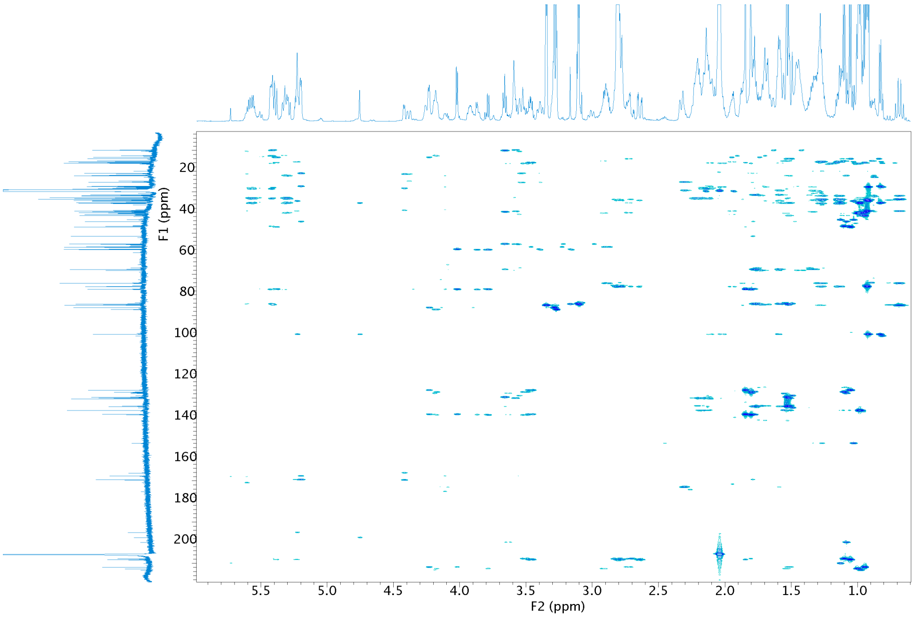

Supplementary Data 71. Chemical structure and 2D NMR key correlations for compound **16**

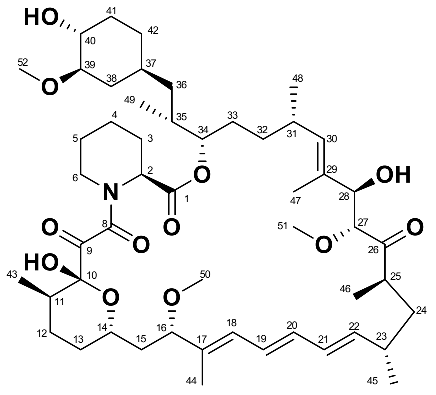

16

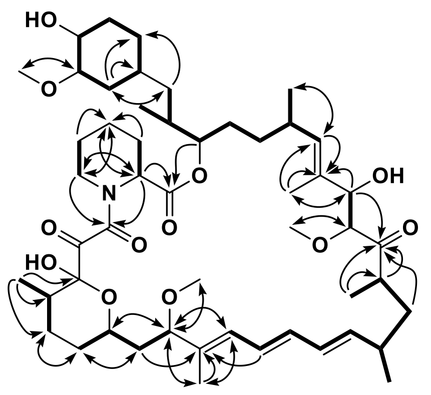

Supplementary Data 72. NMR data for compound **16** in acetone-d_6 at 600 MHz for ^{1}H and 150 MHz for ^{13}C.

Position	δ_C	δ_H (multiplicity, J in Hz)	Position	δ_C	δ_H (multiplicity, J in Hz)
1	170.5	-	27	87.7	3.49 (d, 9.0)
2	50.6	5.12 (m)	28	77.3	4.10 (m)
3	27.3	2.12 (m), 1.75 (m)	29	133.5	-
4	20.9	1.74 (m), 1.44 (m)	30	135.7	5.03 (d, 9.0)
5	25.2	1.65 (m), 1.41 (m)	31	32.6	2.30 (m)
6	43.9	3.64 (m), 3.49 (m)	32	35.3	1.33 (m), 0.90 (m)
7	-	-	33	29.5	1.35 (m)
8	168.0	-	34	79.4	4.76 (m)
9	199.5	-	35	34.6	1.75 (m)
10	99.6	-	36	39.0	1.30 (m), 1.12 (m)
11	35.8	2.23 (m)	37	33.6	1.42 (m)
12	26.7	1.60 (m), 1.58 (m)	38	35.5	0.68 (m), 2.11 (m)
13	30.8	1.87 (m), 1.23 (m)	39	85.0	2.92 (m)
14	67.3	4.07 (m)	40	74.3	3.30 (m)
15	41.2	2.07 (m), 1.32 (m)	41	32.8	1.87 (m), 1.27 (m)
16	83.8	3.62 (m)	42	32.0	1.64 (m), 0.98 (m)
17	138.3	-	43	15.3	0.85 (d, 6.8)
18	128.5	6.08 (d,10.8)	44	10.3	1.78 (s)
19	127.1	6.51 (dd, 14.0, 10.8)	45	21.7	1.01 (d, 6.0)
20	133.6	6.27 (dd, 14.0, 11.4)	46	15.9	1.03 (d, 6.0)
21	130.1	6.25 (dd, 14.0, 11.4)	47	11.1	1.60 (s)
22	140.9	5.51 (dd, 14.0, 9.6)	48	21.7	0.88 (d, 6.0)
23	36.5	2.23 (m)	49	15.9	0.89 (d, 6.0)
24	41.0	1.56 (m), 1.20 (m)	50	55.4	3.10 (s)
25	37.3	2.93 (m)	51	58.2	3.24 (s)
26	212.9	-	52	56.6	3.37 (s)

Supplementary Data 73. ^{1}H NMR spectrum (Acetone-d_6, 600 MHz) of compound **16**.

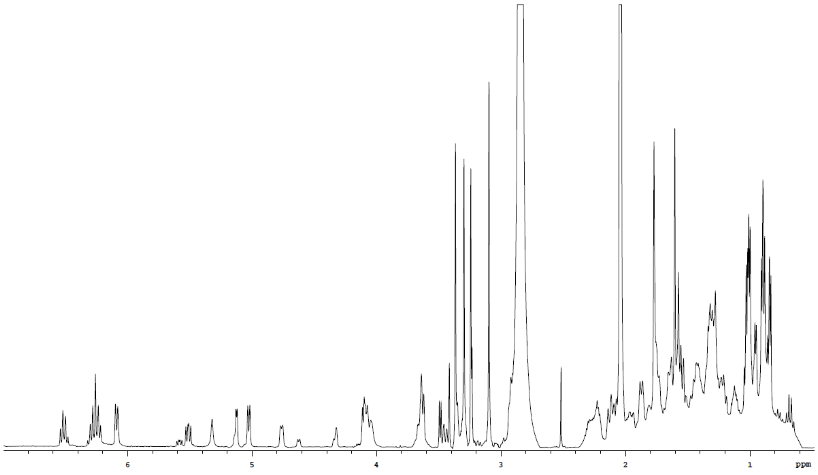

Supplementary Data 74. ^{13}C NMR spectrum (Acetone-d_6, 150 MHz) of compound **16**.

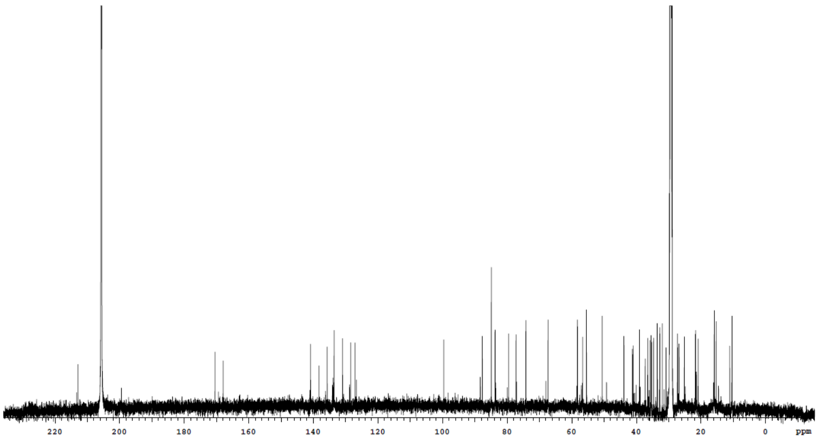

Supplementary Data 75. DQF-COSY NMR spectrum (Acetone-d_6, 600 MHz) of compound **16**.

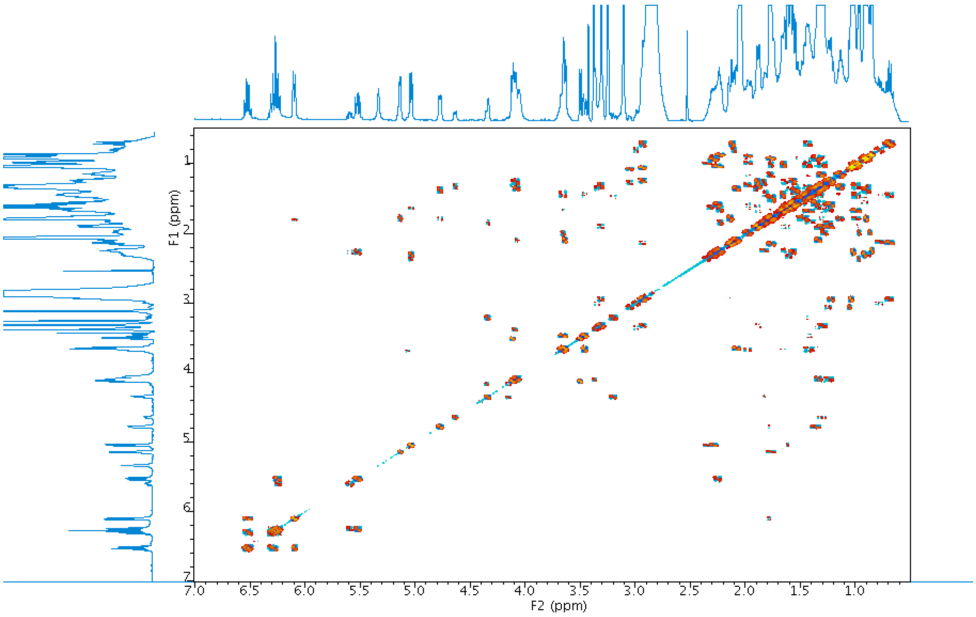

Supplementary Data 76. HSQC NMR spectrum (Acetone-d_6, 600 MHz) of compound **16**.

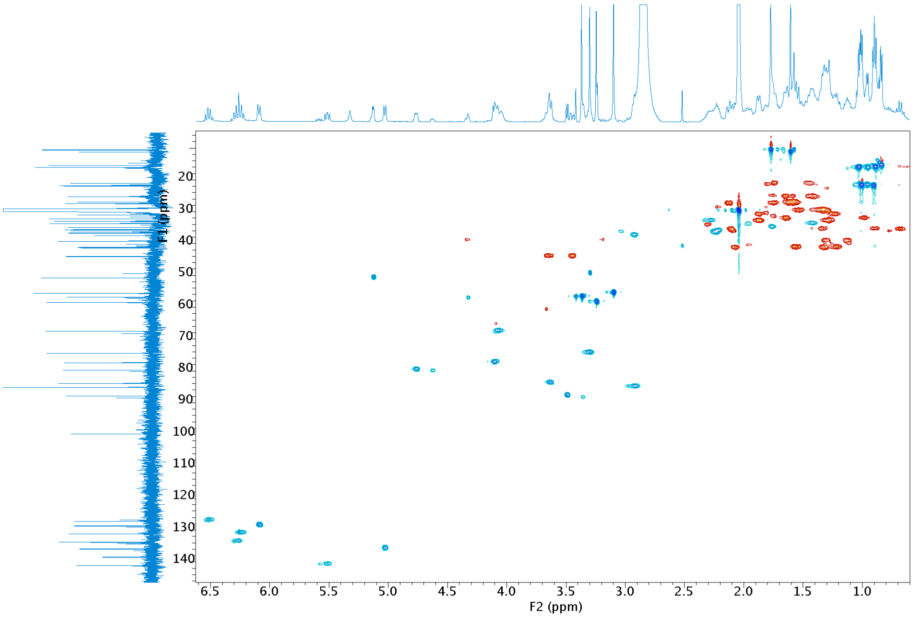

Supplementary Data 77. HMBC NMR spectrum (Acetone-d_6, 600 MHz) of compound **16**.

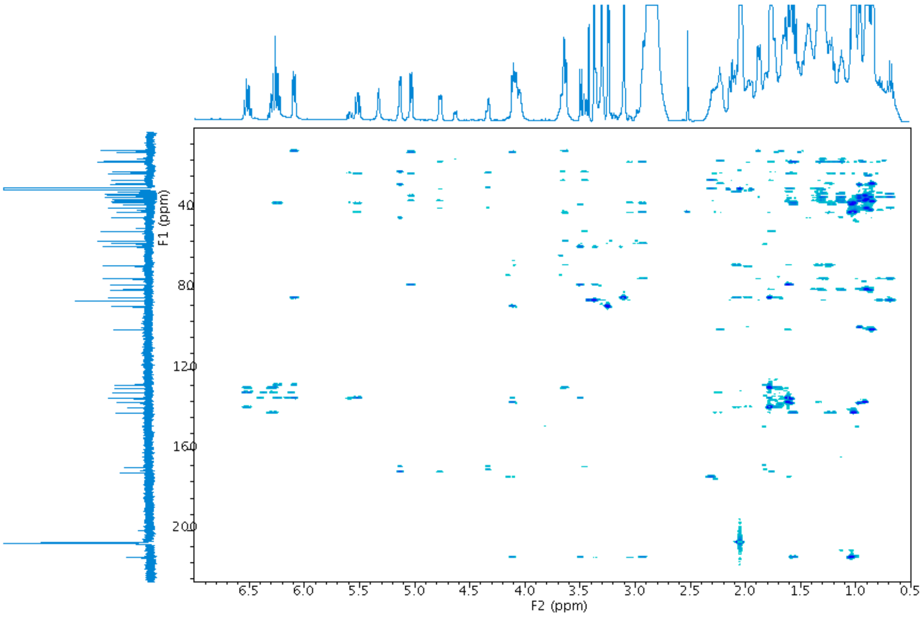

Supplementary Data 78. Chemical structure and 2D NMR key correlations for compound **17**.

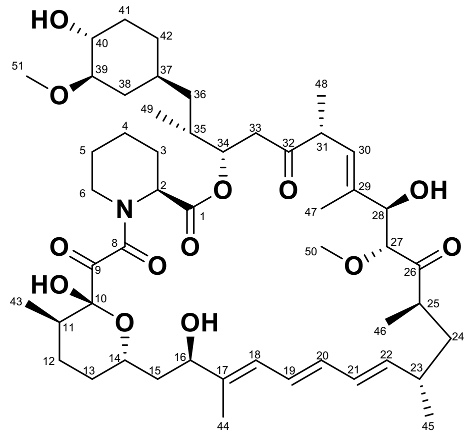

17

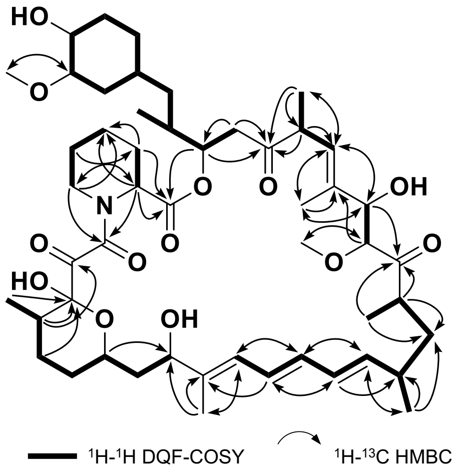

Supplementary Data 79. NMR data for compound **17** in Acetone-d_6 at 600 MHz for ^{1}H and 150 MHz for ^{13}C.

Position	δ_C	δ_H (multiplicity, J in Hz)	Position	δ_C	δ_H (multiplicity, J in Hz)
1	170.5	-	27	86.2	4.17 (m)
2	52.7	5.09 (m)	28	77.5	4.28 (m)
3	27.2	2.29 (m), 1.71 (m)	29	137.3	-
4	21.8	1.73 (m), 1.55 (m)	30	126.4	5.37 (d, 9.9)
5	25.9	1.58 (m), 1.44 (m)	31	46.3	3.40 (m)
6	45.1	3.56 (m), 3.19 (m)	32	208.4	-
7	-	-	33	41.1	2.92 (m), 2.56 (m)
8	168.0	-	34	74.7	5.24 (m)
9	198.8	-	35	34.8	1.86 (m)
10	100.2	-	36	39.9	1.23 (m), 1.10 (m)
11	35.9	2.22 (m)	37	34.3	1.39 (m)
12	28.0	1.64 (m)	38	36.3	2.06 (m), 0.67 (m)
13	33.1	1.74 (m), 1.46 (m)	39	85.5	2.90 (m)
14	68.8	4.25 (m)	40	74.8	3.28 (m)
15	43.5	1.69 (m), 1.65 (m)	41	33.4	1.87 (m), 1.28 (m)
16	72.4	4.17 (m)	42	30.5	1.67 (m), 0.92 (m)
17	143.0	-	43	16.4	0.86 (d, 6.6)
18	124.2	6.22 (d, 11.0)	44	15.2	1.78 (s)
19	128.9	6.49 (dd, 14.6, 11.0)	45	22.1	1.03 (d, 6.9)
20	133.0	6.28 (dd, 14.6, 10.4)	46	13.7	0.91 (d, 6.0)
21	131.7	6.22 (dd, 15.0, 10.4)	47	14.8	1.87 (s)
22	138.2	5.54 (dd, 15.0, 9.2)	48	16.7	0.99 (d, 6.6)
23	36.0	2.33 (m)	49	16.0	0.90 (d, 6.2)
24	39.9	1.55 (m), 1.16 (m)	50	57.9	3.26 (s)
25	41.7	2.55 (m)	51	57.3	3.36 (s)
26	210.9	-			

Supplementary Data 80. ^{1}H NMR spectrum (Acetone-d_6, 600 MHz) of compound **17**.

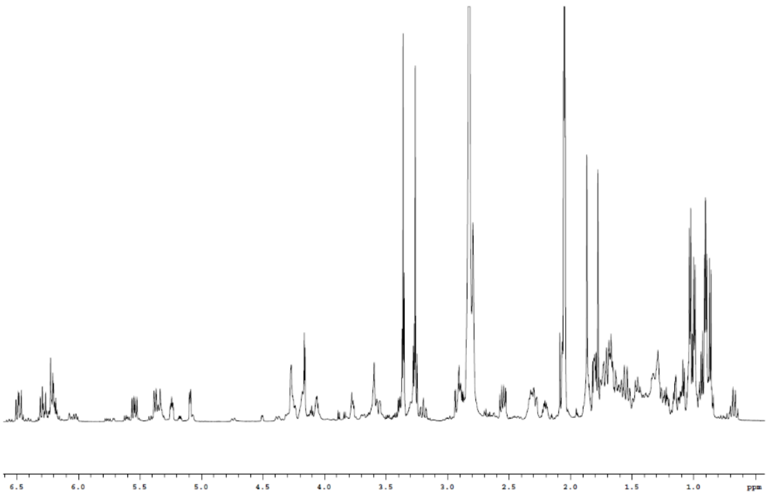

Supplementary Data 81. ^{13}C NMR spectrum (Acetone-d_6, 150 MHz) of compound **17**.

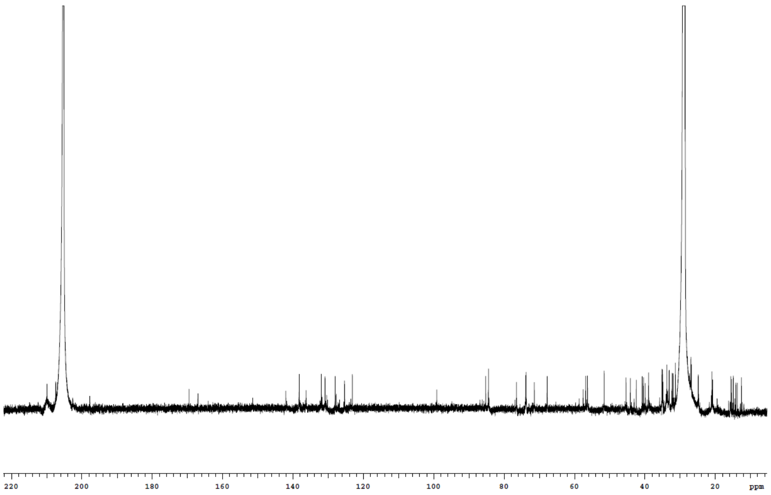

Supplementary Data 82. DQF-COSY NMR spectrum (Acetone-d_6, 600 MHz) of compound **17**.

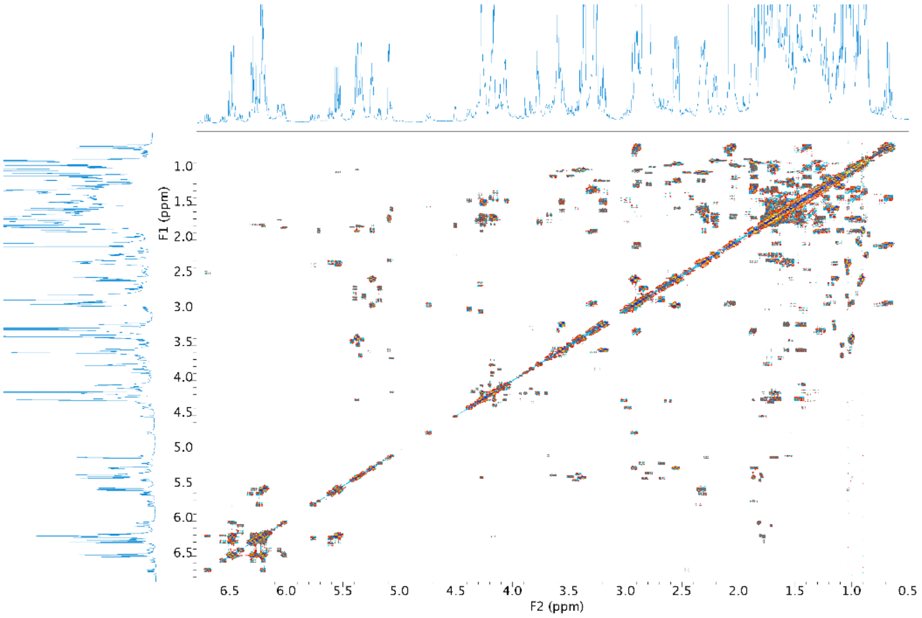

Supplementary Data 83. HSQC NMR spectrum (Acetone-d_6, 600 MHz) of compound **17**.

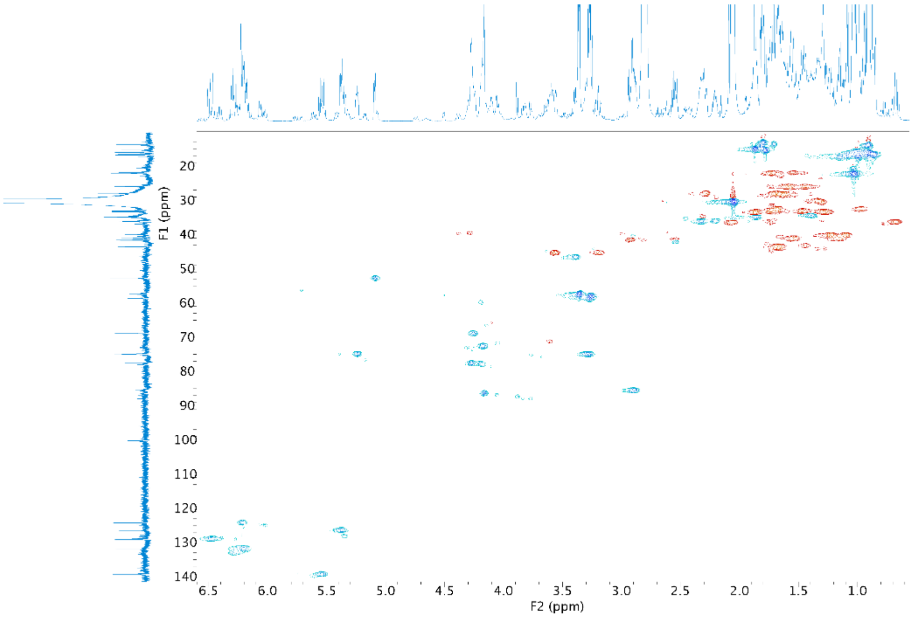

Supplementary Data 84. HMBC NMR spectrum (Acetone-d_6, 600 MHz) of compound **17**.

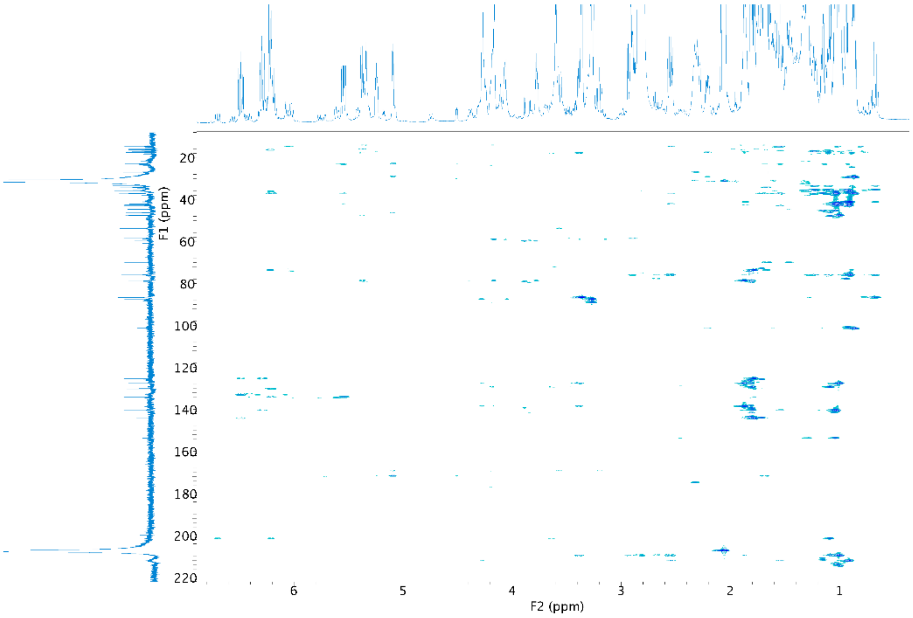

Supplementary Data 85. Chemical structure and 2D NMR key correlations for compound **20**.

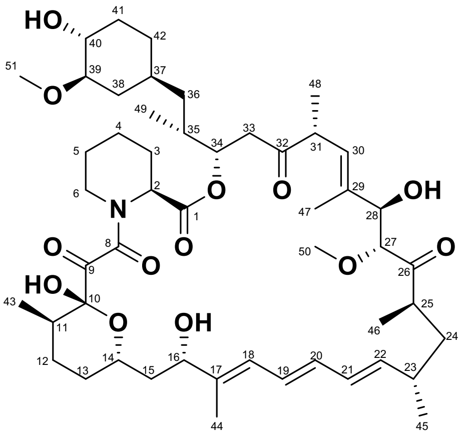

20

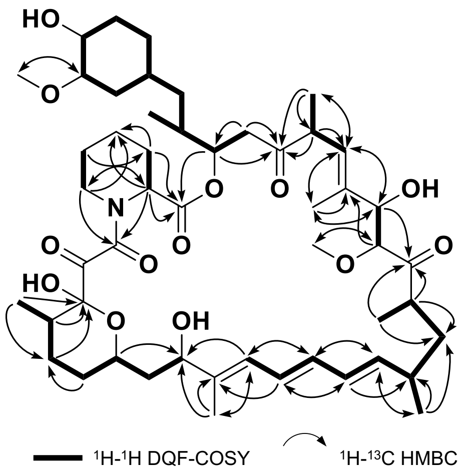

Supplementary Data 86. NMR data for compound **20** in Acetone-d_6 at 600 MHz for ^{1}H and 150 MHz for ^{13}C.

Position	δ_C	δ_H (multiplicity, J in Hz)	Position	δ_C	δ_H (multiplicity, J in Hz)
1	169.9	-	27	85.5	4.11 (m)
2	51.7	5.12 (m)	28	77.0	4.23 (m)
3	27.0	2.27 (m), 1.68 (m)	29	137.1	-
4	21.1	1.72 (m), 1.52 (m)	30	125.9	5.30 (d, 10.6)
5	25.2	1.61 (m), 1.41 (m)	31	46.3	3.38 (m)
6	44.4	3.51 (m), 3.31 (m)	32	207.8	-
7	-	-	33	40.5	2.82 (m), 2.54 (m)
8	167.4	-	34	74.7	5.14 (m)
9	197.2	-	35	34.0	1.85 (m)
10	99.3	-	36	39.3	1.19 (m), 1.08 (m)
11	35.1	2.16 (m)	37	33.4	1.39 (m)
12	27.2	1.61 (m)	38	35.6	2.06 (m), 0.67 (m)
13	30.9	1.88 (m), 1.37 (m)	39	84.8	2.89 (m)
14	68.2	4.14 (m)	40	74.3	3.28 (m)
15	41.9	1.85 (m), 1.43 (m)	41	32.6	1.86 (m), 1.27 (m)
16	73.9	4.21 (m)	42	31.7	1.67 (m), 0.95 (m)
17	141.8	-	43	15.8	0.86 (d, 6.6)
18	124.9	6.09 (d, 10.8)	44	11.1	1.76 (s)
19	128.2	6.45 (dd, 14.4, 10.8)	45	21.3	1.01 (d, 6.6)
20	132.5	6.26 (dd, 14.4, 10.8)	46	13.2	0.91 (d, 6.6)
21	131.2	6.18 (dd, 14.4, 10.8)	47	13.8	1.86 (s)
22	139.0	5.51 (dd, 14.4, 9.0)	48	15.5	0.96 (d, 6.6)
23	35.6	2.30 (m)	49	15.1	0.88 (d, 6.6)
24	40.4	1.51 (m), 1.13 (m)	50	57.3	3.25 (s)
25	41.1	2.56 (m)	51	56.7	3.36 (s)
26	210.7	-			

Supplementary Data 87. ^{1}H NMR spectrum (Acetone-d_6, 600 MHz) of compound **20**.

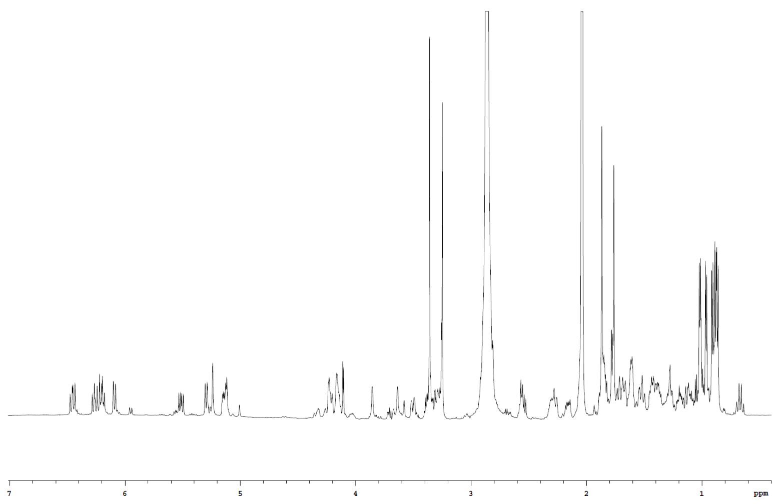

Supplementary Data 88. ^{13}C NMR spectrum (Acetone-d_6, 150 MHz) of compound **20**.

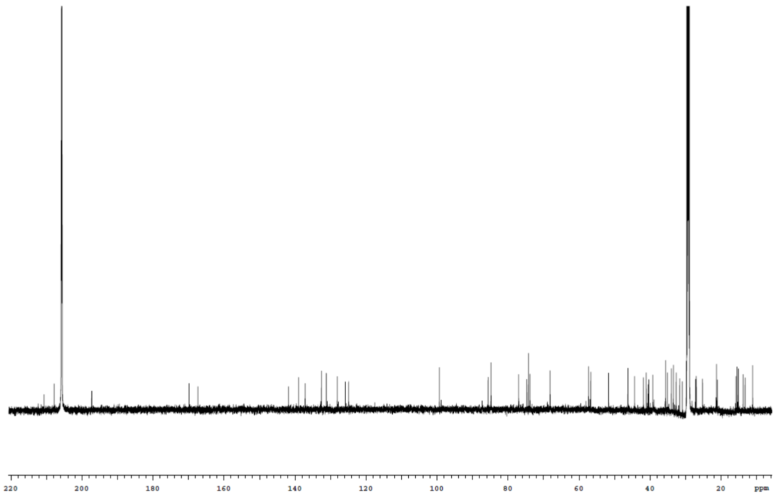

Supplementary Data 89. DQF-COSY NMR spectrum (Acetone-d_6, 600 MHz) of compound **20**.

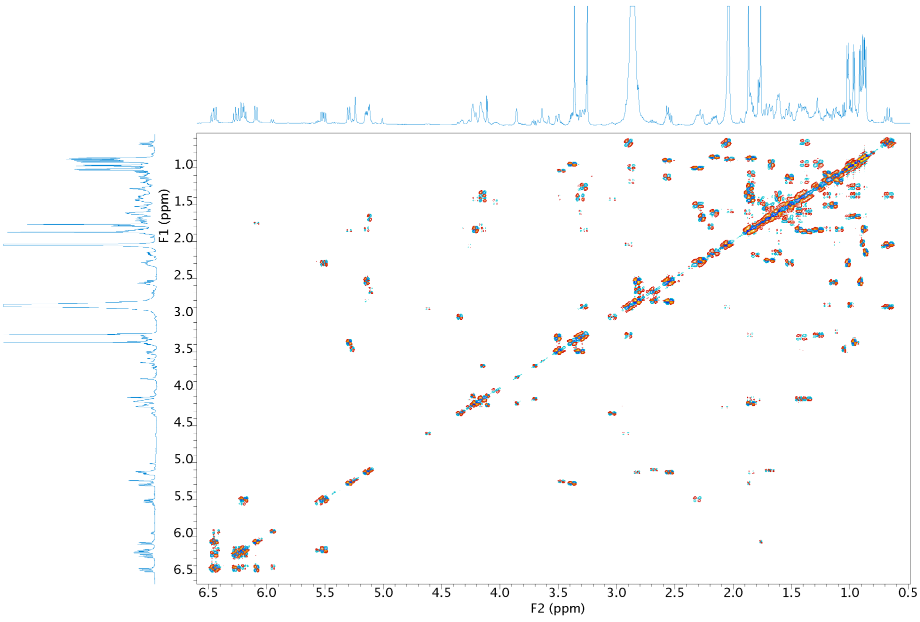

Supplementary Data 90. HSQC NMR spectrum (Acetone-d_6, 600 MHz) of compound **20**.

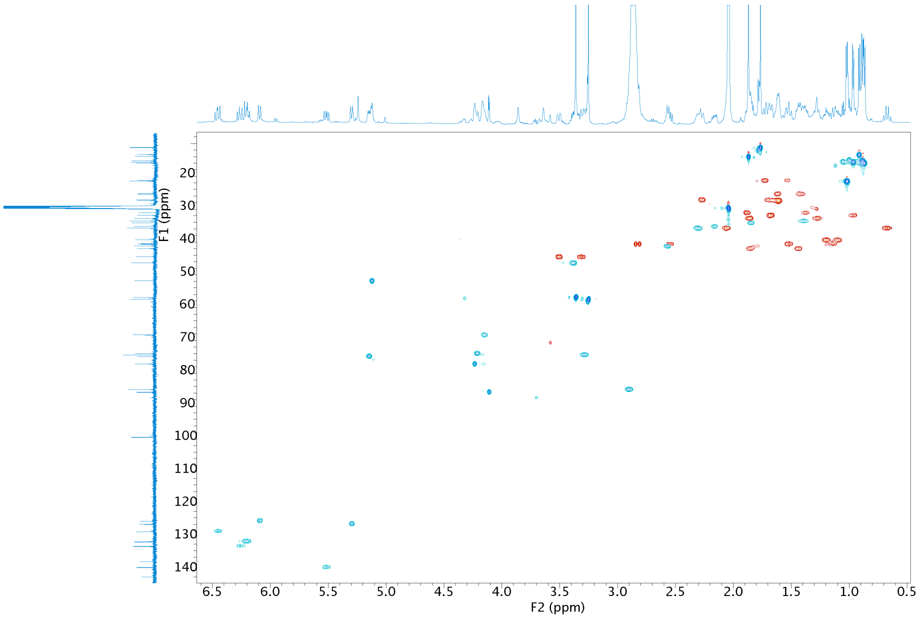

Supplementary Data 91. HMBC NMR spectrum (Acetone-d_6, 600 MHz) of compound **20**.

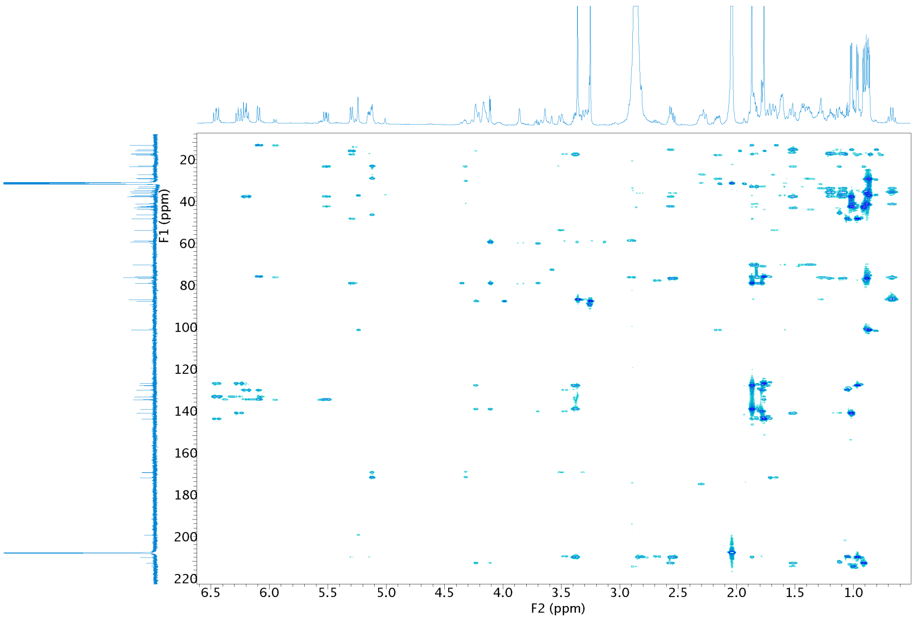

Supplementary Data 92. ^{1}H NMR spectrum (Acetone-d_6, 600 MHz) of Rapamycin (**1**).

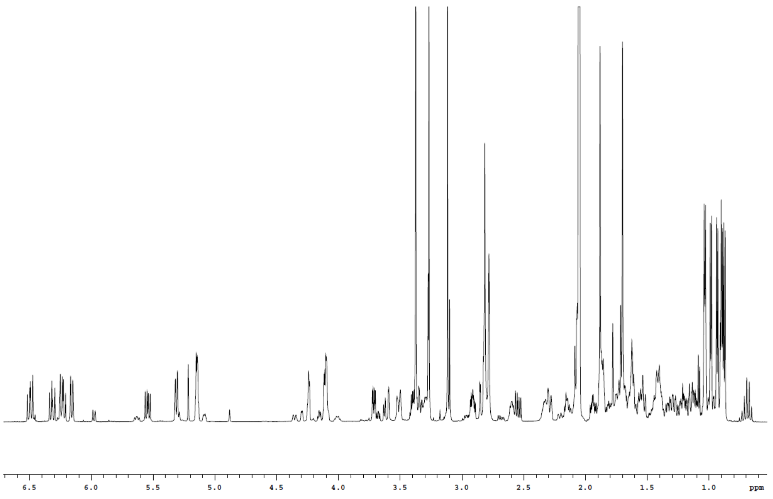

Supplementary Data 93. ^{13}C NMR spectrum (Acetone-d_6, 150 MHz) of Rapamycin (**1**).

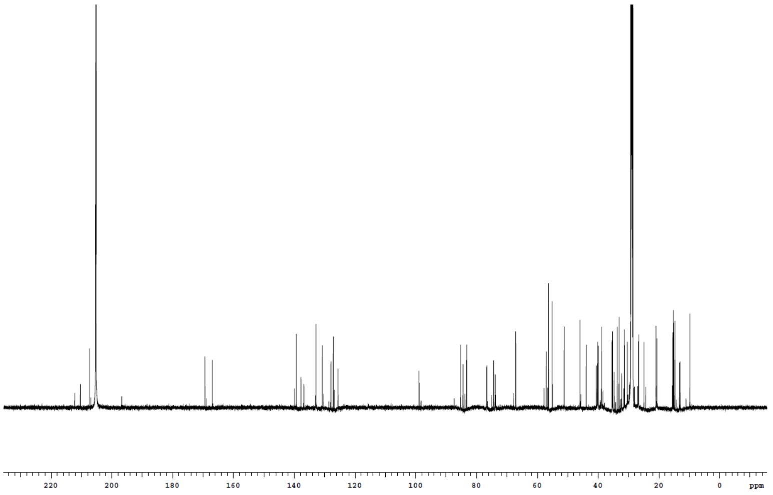

References

1 Vidal, C., Kirchner, G. I. & Sewing, K.-F. Structural elucidation by electrospray mass spectrometry: An approach to the in vitro metabolism of the macrolide immunosuppressant SDZ RAD. *Journal of the American Society for Mass Spectrometry* **9**, 1267-1274 (1998).

2 Luengo, J. I., Konialian-Beck, A., Rozamus, L. W. & Holt, D. A. Manipulation of the Rapamycin Effector Domain. Selective Nucleophilic Substitution of the C7 Methoxy Group. *The Journal of Organic Chemistry* **59**, 6512-6513 (1994).
