# Supplementary material for: In vitro Cas9-assisted editing of modular polyketide synthase genes to produce desired natural product derivatives
Source: Nat Commun. 2020 Aug 11;11:4022. doi: 10.1038/s41467-020-17769-2 (PMC7419507; doi:10.1038/s41467-020-17769-2)
Supplement: Supplementary file 3 — Reporting Summary [file 41467_2020_17769_MOESM3_ESM.pdf]

nature research

Corresponding author(s): Kazuo Shin-ya

Last updated by author(s): Jul 7, 2020

Reporting Summary

Nature Research wishes to improve the reproducibility of the work that we publish. This form provides structure for consistency and transparency in reporting. For further information on Nature Research policies, see Authors & Referees and the Editorial Policy Checklist .

Statistics

For all statistical analyses, confirm that the following items are present in the figure legend, table legend, main text, or Methods section.

n/a	Confirmed	
☐	☒	The exact sample size (n) for each experimental group/condition, given as a discrete number and unit of measurement
☐	☒	A statement on whether measurements were taken from distinct samples or whether the same sample was measured repeatedly
☒	☐	The statistical test(s) used AND whether they are one- or two-sided<br>*Only common tests should be described solely by name; describe more complex techniques in the Methods section.*
☒	☐	A description of all covariates tested
☒	☐	A description of any assumptions or corrections, such as tests of normality and adjustment for multiple comparisons
☐	☒	A full description of the statistical parameters including central tendency (e.g. means) or other basic estimates (e.g. regression coefficient) AND variation (e.g. standard deviation) or associated estimates of uncertainty (e.g. confidence intervals)
☒	☐	For null hypothesis testing, the test statistic (e.g. F, t, r) with confidence intervals, effect sizes, degrees of freedom and P value noted<br>*Give P values as exact values whenever suitable.*
☒	☐	For Bayesian analysis, information on the choice of priors and Markov chain Monte Carlo settings
☒	☐	For hierarchical and complex designs, identification of the appropriate level for tests and full reporting of outcomes
☒	☐	Estimates of effect sizes (e.g. Cohen's d, Pearson's r), indicating how they were calculated

Our web collection on statistics for biologists contains articles on many of the points above.

Software and code

Policy information about availability of computer code

Data collection	MassLynX V4.1 was used for acquisition of mass spectra on an XevoG2 ToF (Waters). Xcalibur V4.0.27.42 was used for acquisition of MS/MS spectra on an Orbitrap Fusion Lumos (Thermo). NMR spectra were acquired on Varian 500 or 600 MHz instrument and collected with VnmrJ V4.2. EnVision Multi label Plate Reader (Perkin Elmer) and its accompanied software was used to collect luminescence data.
Data analysis	Geneious prime V2019.2.1 was used to analyse genetic data. MassLynx V4.1 was used for analysis of chromatograms. Xcalibur V4.0.27.42 was used for analysis of MS/MS spectra. NMR spectra were analysed using VnmrJ V4.2 and Mnova V14.1.2 (Mestrelab). Harmony4.9 software (PerkinElmer) was used for analysis of cell images. TIBCO Spotfire 10.3 software (PerkinElmer) was used for calculation of EC50 values.

For manuscripts utilizing custom algorithms or software that are central to the research but not yet described in published literature, software must be made available to editors/reviewers. We strongly encourage code deposition in a community repository (e.g. GitHub). See the Nature Research guidelines for submitting code & software for further information.

Data

Policy information about availability of data

All manuscripts must include a data availability statement. This statement should provide the following information, where applicable:

- Accession codes, unique identifiers, or web links for publicly available datasets
- A list of figures that have associated raw data
- A description of any restrictions on data availability

Sequence data that support the findings of this study have been deposited in GenBank with the accession code LC566301. Source data underlying Table 1 and Supplementary Fig. 11 are provided with this paper. The other data that support the findings of this study are included in this paper. Raw data for spectroscopic analyses are available from the corresponding author upon reasonable request.

Field-specific reporting

Please select the one below that is the best fit for your research. If you are not sure, read the appropriate sections before making your selection.

- [x] Life sciences
- [ ] Behavioural & social sciences
- [ ] Ecological, evolutionary & environmental sciences

For a reference copy of the document with all sections, see nature.com/documents/nr-reporting-summary-flat.pdf

Life sciences study design

All studies must disclose on these points even when the disclosure is negative.

Sample size	Sample-size calculation was not performed. All plots in "Biological activities" section indicate mean ± s.d. calculated from three biological replicates.
Data exclusions	No data were excluded from the analyses.
Replication	In the Figure legend, we stated how many number of replications were performed. All attempts at replication were successful.
Randomization	Not relevant due to in vitro nature of our study.
Blinding	Not relevant due to in vitro nature of our study.

Reporting for specific materials, systems and methods

We require information from authors about some types of materials, experimental systems and methods used in many studies. Here, indicate whether each material, system or method listed is relevant to your study. If you are not sure if a list item applies to your research, read the appropriate section before selecting a response.

Materials & experimental systems

n/a	Involved in the study	
[x]	[ ]	Antibodies
[ ]	[x]	Eukaryotic cell lines
[x]	[ ]	Palaeontology
[x]	[ ]	Animals and other organisms
[x]	[ ]	Human research participants
[x]	[ ]	Clinical data

Methods

n/a	Involved in the study	
[x]	[ ]	ChIP-seq
[x]	[ ]	Flow cytometry
[x]	[ ]	MRI-based neuroimaging

Eukaryotic cell lines

Policy information about cell lines

Cell line source(s)	T lymphoma Jurkat cells was purchased from RIKEN BRC. Human ovarian adenocarcinoma SKOV-3 cells was purchased from ATCC. Malignant pleural mesothelioma MESO-1 cells were provided by Dr. Yoshitaka Sekido (Aichi Cancer Center), who deposited the cell line to RIKEN BRC. HeLa cells were provided by JCRB.
Authentication	None of the cell lines used were authenticated.
Mycoplasma contamination	None of the cell lines used were tested for mycoplasma contamination.
Commonly misidentified lines (See ICLAC register)	No commonly misidentified cell lines were used.
